# Structural requirements of blood factors binding to soluble hexon trimers with implications for adenovirus cell targeting and immune evasion

**DOI:** 10.1371/journal.ppat.1014389

**Published:** 2026-07-13

**Authors:** Olivia X. Ma, Shao-Chia Lu, Haley E. Mudrick, Mary E. Barry, Jarrod B. French, Michael A. Barry, Vijay S. Reddy

**Affiliations:** 1 The Hormel Institute, University of Minnesota, Austin, Minnesota, United States of America; 2 Department of Internal Medicine, Division of Infectious Diseases, Mayo Clinic, Rochester, Minnesota, United States of America; 3 Molecular Pharmacology and Experimental Therapeutics Graduate Program, Mayo Clinic, Rochester, Minnesota, United States of America; 4 Department of Immunology, Department of Molecular Medicine, Mayo Clinic, Rochester, Minnesota, United States of America; University of Wisconsin-Madison School of Medicine and Public Health, UNITED STATES OF AMERICA

## Abstract

Human adenovirus serotype 5 (HAdV-C5) is widely used as a gene delivery vector in both experimental and clinical settings. Upon intravenous administration, HAdV-C5 exhibits strong liver tropism, largely mediated by interactions between its major capsid protein, hexon (Hx), and coagulation factor X (FX). In contrast, the closely related species C adenovirus 6 (HAdV-C6) also targets the liver but shows reduced dependency on coagulation factors, whereas species D adenovirus 26 (HAdV-D26) does not bind coagulation factors altogether. To define the structural basis of this serotype-specific host factor recognition, we determined high-resolution cryo-electron microscopy structures of isolated hexon trimers from HAdV-C5 and HAdV-C6 in complex with coagulation factors FX and prothrombin (factor II, FII). The resulting atomic models reveal conserved binding interfaces involving the γ-carboxyglutamic acid (Gla) domains of both coagulation factors and the hypervariable regions HVR5 and HVR7 lining the surface cavities of HAdV-C5 and HAdV-C6 hexons. Structures of hexon complexes formed by co-incubation with both FX and FII further reveal serotype-specific binding preferences under physiologically relevant conditions, showing that HAdV-C5 hexon preferentially engages FX, whereas HAdV-C6 hexon favors FII. By contrast, HAdV-D26 hexon does not bind either factor, likely due to an insertion constrained by proline residues in the HVR5 loop that restricts the factor access to the hexon cavity. Together, these findings provide a detailed structural framework for adenovirus-coagulation factor interactions and support the rational engineering of adenovirus vectors with improved targeting and safety profiles.

## Author summary

Viruses have evolved ingenious ways of masking themselves to evade detection and clearance by the host immune system. In the bloodstream, species-C human adenoviruses (HAdVs) coat themselves with the coagulation factors to avoid identification by the antibodies and subsequent opsonization by macrophages. Additionally, binding of the factors target the HAdV derived vectors to the liver. These blood factors are known to bind in the cavity of hexons, the most abundant major capsid protein of adenoviruses. To understand the structural basis of hexon-coagulation factor interactions in greater detail, we determined the cryo-EM structures of purified hexons from two species-C adenoviruses in complex with the blood factors: Factor X and Factor II at near-atomic (~3.2Å) resolution. The results highlight the similarities in the Gla-domains of the factors binding in the hexon cavities. Additionally, the variance in the factor binding appears due to differences in the entrances to the hexon cavities and the allosteric effects associated with the factor binding. Furthermore, we suggested a likely structural basis why the species-D HAdVs (e.g., HAdV-D26) do not bind the same blood factors. We believe that these results provide new knowledge in using the HAdV vectors intravenously as gene and vaccine delivery vehicles.

## Introduction

Human adenoviruses (HAdVs or simply Ads) are non-enveloped, double-stranded DNA viruses that exhibit significant genetic diversity, with over 200 genotypes classified into 7 species (A-G), according to the International Committee on Taxonomy of Viruses [1–5] and the human adenovirus working group (http://hadvwg.gmu.edu/). Due to their high transduction efficiency and greater genetic payload capacity (7–12 kb), Ads have become prominent vectors in clinical gene therapy and vaccine research [6–11]. Human Adenovirus serotype 5 (HAdV-C5), belonging to species C Ads, has been used extensively due to its well-characterized genome, robust gene expression, and established production processes [6–8]. Despite these advantages, HAdV-C5 vectors face challenges including unintended liver targeting and immune activation following intravenous administration, primarily driven by interactions between the adenovirus capsid, especially its hexon protein, and circulating host proteins like blood coagulation factors and antibodies, respectively [6,12–16].

**Fig 1:**
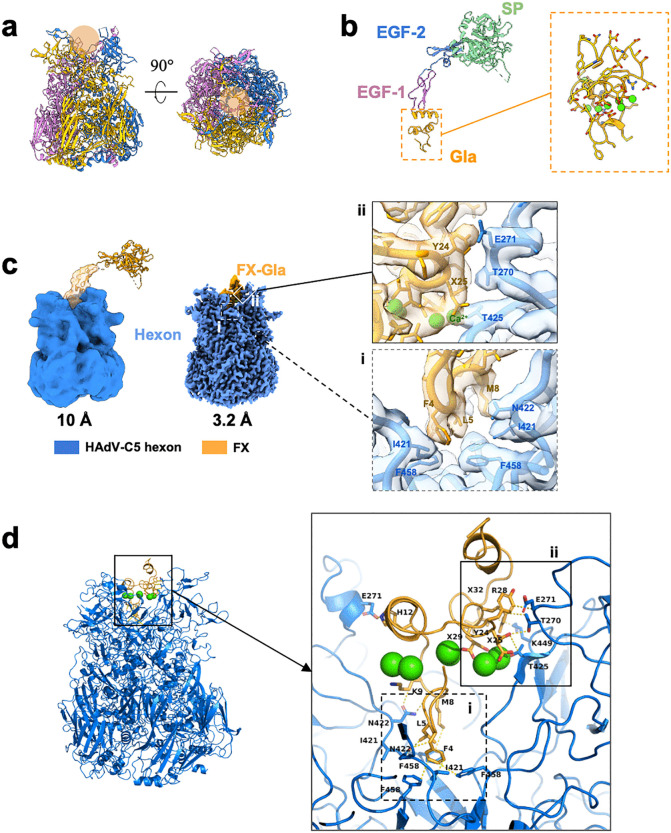
Cryo-EM structure of HAdV-C5 hexon-FX complex. **a**) atomic model of the HAdV-C5 hexon trimer (PDB: 1P30), with the three monomers shown in different colors. The location of the hexon cavity, where coagulation factor binds, is indicated by a transparent sphere. **b**) AlphaFold2-predicted model of FX, with different domains - Gla (orange), EGF-1 (purple), EGF-2 (blue), and serine protease domain (green) - identified. Inset shows the modified Gla domain with post-translational modifications (γ-carboxylation of glutamic acids) and coordinating Ca² ⁺ ions modeled based on the FX-snake venom binding protein complex (PDB: 1IOD). **c**) Cryo-EM density map of Ad5Hx-FX complex and interacting regions. Left: the density map of a selected class of FX-bound complex after focused 3D classification, showing clear density for the FX (orange) bound within the hexon (blue) cavity. The AlphaFold2-predicted FX model is fitted into the corresponding factor density. Middle: density map of the Ad5Hx-FX complex at 3.2 Å resolution. Right: the enlarged boxed panels highlighting the two Gla domain interacting regions; (**i**) hydrophobic region formed by the HVR7 residues at the bottom of the hexon cavity and (**ii**) contacts at the cavity entrance mediated by the residues in the HVR5 loop. **d**) Ribbon diagram of the Ad5Hx-FX complex derived from the 3.2 Å density map. The enlarged view shows all contacting residues from HVR7 (region **i**) and the interacting residues from HVR5 loop (region **ii**). The FX domains other than the interacting Gla domain are disordered at high resolution.

Hexon, the major building block of the adenovirus capsid, assembles into stable trimers (**Fig 1a**) [17]. A total of 240 copies of hexon trimers, along with 60 copies of penton base, 36 copies of fiber and multiple copies of various minor proteins, form an icosahedral capsid with pseudo-T = 25 icosahedral symmetry [18,19]. Each hexon monomer contains seven outward-facing hypervariable regions (HVRs), which differ substantially among adenovirus serotypes [20]. These HVRs play critical roles in vector targeting, biodistribution and immune recognition [6,21–23].

Previous studies have shown that circulating host proteins, particularly blood coagulation factors, significantly influence adenovirus vector biodistribution and immunogenicity [6,12,14–16,24–26]. Coagulation factor X (FX), in particular, binds to HAdV-C5 hexon trimers with high affinity via its gamma-carboxyglutamic acid (Gla) domain (**Fig 1b**) [16,27,28], promoting robust hepatic tropism upon systemic administration of HAdV-C5 vectors [6,24,29]. Mutagenesis studies have identified specific residues in HVR5 and HVR7 involved in FX binding [29]. Although FX-mediated hepatic targeting benefits liver-directed therapies, it limits applications targeting other tissues and may induce liver toxicity at higher vector doses [6,24].

Prothrombin (FII), another critical coagulation factor present in the blood at plasma concentrations approximately tenfold higher than FX, can also bind and shield adenovirus vectors [14,15]. Both FII and FX exhibit variable binding affinities to species C adenoviruses, influencing the vector biodistribution and immune clearance [14,15]. However, the structural basis underlying these serotype-specific differences in factor binding remains incompletely understood.

In this study, we employed single-particle cryo-electron microscopy (cryo-EM) to define the interactions between species C adenovirus hexon proteins from HAdV-C5 and HAdV-C6 and the human coagulation factors FX and FII at near-atomic resolution. In contrast to previous studies using intact virions, where factor density was ambiguous due to insufficient local resolution, we utilized isolated hexon trimers to resolve the interaction interface at residue-level detail.

This study provided several advances beyond prior work. First, we identified conserved factor-specific interactions within the hypervariable regions HVR5 and HVR7 that govern binding of both FX and FII. Second, by co-incubating hexons with both factors at a 1:10 molar ratio reflecting their relative physiological abundance, we found distinct binding preferences between the closely related serotypes, with HAdV-C5 hexon preferentially engaging FX, while HAdV-C6 hexon favoring FII. Third, we provide the structural reasons for the lack of coagulation factor binding in the species D adenovirus HAdV-D26, resulting from steric constraints within the HVR5 loop. Together, these findings establish a detailed structural basis for adenovirus–coagulation factor interactions and provide new insights into how serotype-specific differences may influence vector tropism and immune evasion with implications for the rational design of improved adenovirus-based therapeutics.

## Results

To resolve adenovirus–coagulation factor interactions at residue-level detail, we analyzed purified hexon trimers in complex with individual coagulation factors using single-particle cryo-EM. This approach reduces structural heterogeneity observed in virion-based studies and enables improved definition of the interaction interface. Using this strategy, we determined high-resolution structures of HAdV-C5 and HAdV-C6 hexons in complex with FX and FII.

### Cryo-EM structure of the Ad5Hx-FX complex at 3.2 Å

2D class averages of the HAdV-C5 hexon-FX complex (Ad5Hx-FX) revealed well-defined structural features of the hexon trimer, along with a tail-like density emanating from the central cavity (S1 Fig). After removal of low-quality particles through 2D classification and heterogeneous refinement in CryoSPARC [30], the initial reconstruction reached a global resolution of 3.0 Å, as estimated by the FSC 0.143 criterion (S2A-S2C Fig). Local resolution analysis showed relatively uniform resolution across the hexon trimer, with lower resolution at the FX-associated density (S2A Fig), suggesting the presence of both factor-bound and factor-free hexons in the dataset.

To separate different populations and improve the local density of the bound factor, we performed focused 3D classification using a spherical mask placed over the hexon cavity, where FX density was observed (S1 Fig). All of the resulting classes displayed clear density corresponding to the bound factor, sufficient to accommodate the AlphaFold2-predicted FX model at low resolution (10 Å) (Figs 1b-1c, S3A). The AlphaFold2 model was modified as described previously [31] (Fig 1b) to account for the post-translational modification (γ-carboxylation) of glutamic acid residues and to include coordinating Ca² ⁺ ions, based on structural alignment with the FX-snake venom binding protein complex (PDB: 1IOD) [32].

Following additional refinement, the Ad5Hx-FX complex was resolved to an overall resolution of 3.2 Å, allowing clear visualization of side-chain features in the FX Gla domain (Figs 1c and S2D-S2F). The atomic model of the complex was generated by fitting the HAdV-C5 hexon crystal structure (PDB: 1P30) and the modified FX-Gla model into the corresponding density, followed by real-space refinement in Phenix [33] and manual adjustments in Coot [34] (Fig 1c-1d and Table 2). The contacting residues were identified using the CONTACT program in CCP4 [35] with a distance cutoff of 4 Å (Fig 1d and S1 Table).

Analysis of the refined model revealed two distinct binding regions on the HAdV-C5 hexon: one at the bottom of the hexon cavity and another at its entrance (Fig 1c). At the bottom of the hexon cavity, residues I421 and F458 in the HVR7 loops from all three monomers form a hydrophobic “greasy patch” that engages residues F4 and L5 of the FX Gla domain through π-stacking and hydrophobic interactions (Fig 1c–1d). At the cavity entrance, residues T270 and E271 in HVR5 loop of one of the hexon subunits form additional contacts that help stabilize the bound FX Gla domain (Fig 1c-1d). In addition, a calcium-mediated interaction involving hexon residue T425, together with a salt bridge between FX-Gla residue H12 and hexon residue E271, further strengthens the interface (Fig 1d and S1 Table). Our results confirm that HVR5 and HVR7 constitute the major binding regions for FX on the HAdV-C5 hexon. These findings are consistent with previous mutagenesis studies showing that substituting HVR5 and HVR7 of HAdV-C5 with the corresponding regions from HAdV-D26, which does not bind FX, markedly reduces factor binding [29].

With a few differences, the overall refined model of Ad5Hx-FX from this study is similar to the recently published model of Ad5-FX derived from the virus-factor complexes (PDB: 9CLI; RMSD: 0.51 Å) [31]. The primary difference lies in the positioning of FX, with the FX-Gla domain in the new model slightly shifted toward one of the hexon monomers, whereas in the previous model (PDB: 9CLI), it is positioned within the center of the hexon cavity [31] (S4 Fig). Although the density for FX-Gla in this study is strong and well-defined, the densities for the rest of the FX molecule - epidermal growth factor-like (EGF) and serine protease (SP) domains remain unresolvable even after applying a 10-Å resolution low-pass filter (Fig 1c). This is likely due to the high flexibility of these domains and is consistent with our previously published results [31].

### Cryo-EM structure of the Ad5Hx-FII complex at 3.2 Å

Unlike the Ad5Hx-FX complex, the Ad5Hx-FII particles exhibited strong preferred orientations, with a high frequency of top and bottom views in 2D classification (S5A Fig). This phenomenon was also seen in the hexon-alone dataset. To address this issue, we collected two additional datasets, one with a 30° stage tilt [36] and another using graphene-coated grids [37]. Merging these datasets together improved angular distribution of the Ad5Hx-FII complex (S5B Fig), resulting in a 2.8 Å reconstruction. (S6A-S6C Fig).

**Fig 2:**
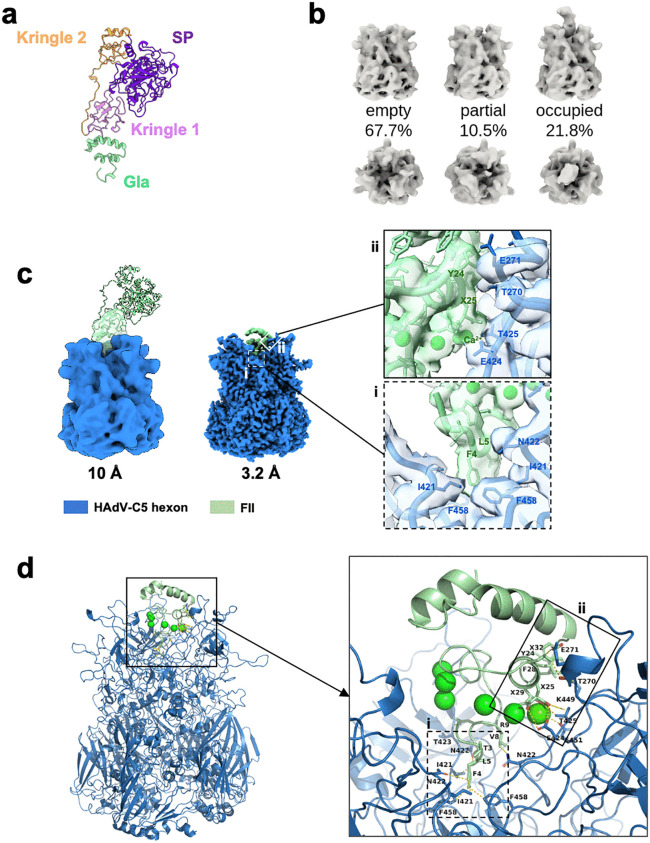
Cryo-EM structure of HAdV-C5 hexon-FII complex. **a**) AlphaFold2-predicted model of human FII, with different domains - Gla (green), Kringle 1 (light purple), Kringle 2 (orange), and serine protease domain (dark purple) - identified. **b**) Results from focused 3D classification of the HAdV-C5 hexon-FII dataset showing (top) the side views of three major classes corresponding to empty (67.7%), partially occupied (10.5%), and fully factor-occupied (21.8%) hexon cavities. Bottom: the top views, 90° rotated from the above side views. **c**) Left: the occupied class (21.8%) from the focused 3D classification showing clear density for the FII (light green) Gla domain bound within the hexon (blue) cavity. The AlphaFold2-predicted FII model is fitted into the corresponding factor density. Middle: density map of the Ad5Hx-FII complex at 3.2 Å resolution. Right: the enlarged boxed panels highlighting the two Gla domain interacting regions; (**i)** hydrophobic region formed by the HVR7 residues at the bottom of the cavity and (**ii)** contacts at the cavity entrance mediated by the residues in HVR5 loop. **d**) Ribbon diagram of the Ad5Hx-FII complex derived from the 3.2 Å map. The enlarged view shows all contacting residues from HVR7 (region **i**) and the interacting from HVR5 loop (region **ii**). The FII domains other than the interacting Gla domain are disordered at high resolution. Ca² ⁺ ions are shown based on expected coordination within the Gla domain; however, not all ions are clearly resolved in the density maps, and their placement is supported by prior structural knowledge and modeling.

The focused 3D classification revealed that only 21.8% of the particles have clear FII density (Fig 2b and S2 Table), and this subset of the particles were selected and refined to a 3.2 Å density map (Figs 2c and S6D-S6F). An AlphaFold2-predicted and γ-carboxylation-incorporated model of FII was fitted into the corresponding density (Figs 2a and 2c, S3b), and the contacting residues are depicted in Fig 2d (S3 Table).

The binding mode of FII closely resembles that of FX. The FII Gla domain also inserts into the hydrophobic pocket formed by the three HVR7 loops at the base of the hexon cavity, while a single HVR5 loop at the cavity entrance provides stabilizing contacts that help orient the bound factor (Fig 2c-2d). Sequence alignment and structural comparison demonstrate that the Gla domains of FX and FII are highly conserved (S7 Fig), supporting a shared binding mechanism with the HAdV-C5 hexon. The factor-free HAdV-C5 hexon (reconstructed from one of the 3D-classes) was refined to 3.1 Å resolution (S8a-S8c Fig). Structural comparison revealed no significant conformational differences between the factor-free hexon and the HAdV-C5 hexon in complex with either FX or FII (S9 Fig). Compared with the previously reported Ad5-FII structure (PDB: 9CLN), the FII Gla domain in the current study shifts slightly towards one of the hexon monomers, with an overall RMSD of 0.78 Å between the structures (S10 Fig) [31].

### Cryo-EM structure of HAdV-C6 hexon-factor complexes

**Fig 3:**
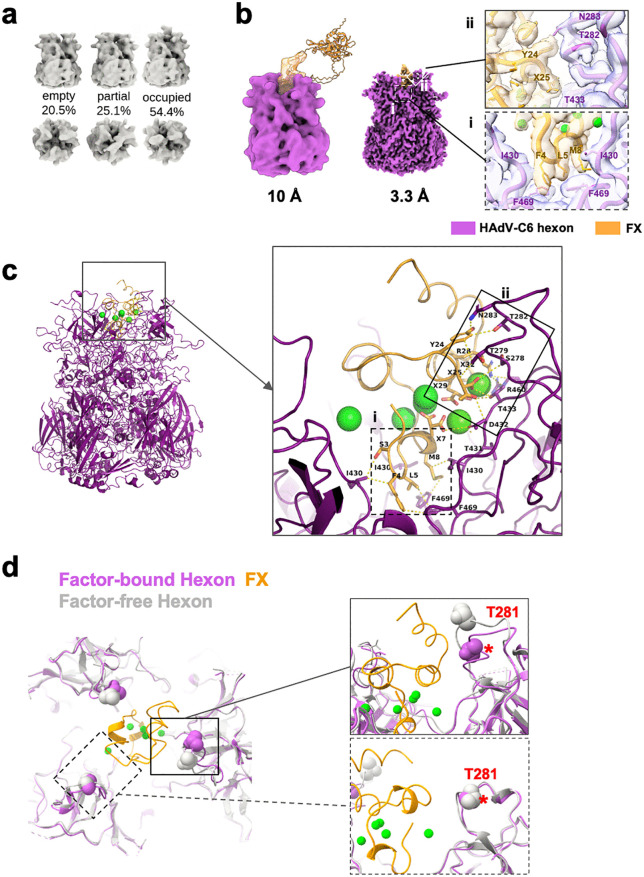
Cryo-EM structure of HAdV-C6 hexon in complex with FX. **a**) Results from focused 3D classification of the Ad6Hx-FX dataset showing (top) the side views of three major classes consisting of empty (20.5%), partially occupied (25.1%), and FX-bound (54.4%) hexons. Bottom: the top views, 90° rotated from the above side views. **b**) Left: the FX-bound class (54.4%) showing clear density for the FX (orange) Gla domain within the HAdV-C6 hexon (purple) cavity at ~10 Å resolution. Middle: density map of the Ad6Hx-FX complex at 3.3 Å resolution. Right: the enlarged boxed panels highlight the two Gla domain interacting regions: (**i)** hydrophobic region formed by HVR7 residues at the bottom of the cavity and (**ii)** contacts at the cavity entrance mediated by residues from HVR5 loop. **c**) Ribbon diagram of the Ad6Hx-FX complex derived from the 3.3 Å map. The enlarged view shows all contacting residues from HVR7 (region **i**) and the interacting residues from HVR5 loop (region **ii**). The FX domains other than the interacting Gla domain are disordered at high resolution. **d**) Structural comparison of the factor-bound and factor-free HAdV-C6 hexons. The factor-free hexon is shown in grey, the FX-bound hexon in purple, FX in orange and the Ca² ⁺ ions are shown in green. Upon FX binding, the interacting HVR5 loop refolds into a short α-helix and thereby expands the entrance to the cavity to accommodate binding of the Gla domain. Insets show the enlarged side views of the interacting HVR5 loop (top) and a non-interacting HVR5 loop (bottom), illustrating that the conformational change is localized only to the HVR5 loop interacting with FX.

**Fig 4:**
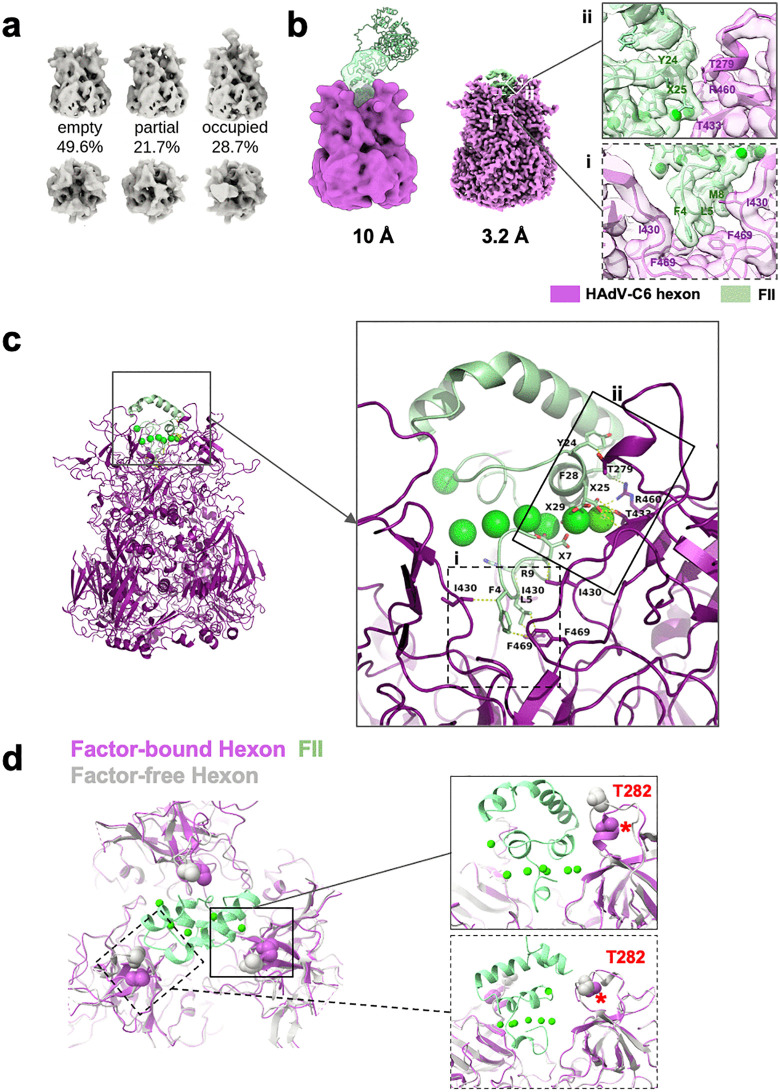
Cryo-EM structure of HAdV-C6 hexon in complex with FII. **a**) Results from focused 3D classification of the Ad6Hx-FII dataset, showing (top) the side views of three major classes consisting of empty (49.6%), partially occupied (21.7%), and FII-bound (28.7%) hexons. Bottom: the top views, 90° rotated from the above side views. **b**) Left: the FII-bound class (28.7%) showing clear density for the FII (light green) Gla domain within the HAdV-C6 hexon (purple) cavity at ~10 Å resolution. Middle: density map of the Ad6Hx-FII complex at 3.2 Å resolution. Right: the enlarged boxed panels highlight the two Gla domain interacting regions: (**i)** hydrophobic region formed by HVR7 residues at the bottom of the cavity and (**ii)** contacts at the cavity entrance mediated by residues in HVR5 loop. **c**) Ribbon diagram of the Ad6Hx-FII complex derived from the 3.2 Å map. The enlarged view shows all contacting residues from HVR7 (region **i**) and the interacting residues from HVR5 loop (region **ii**). The FII domains other than the interacting Gla domain are disordered at high resolution. **d**) Structural comparison of the factor-bound and factor-free HAdV-C6 hexons. The factor-free hexon is shown in grey, the FII-bound hexon in purple, FII in light green and the Ca² ⁺ ions are shown in green. Upon FII binding, the interacting HVR5 loop refolds into a short α-helix, similar to the Ad6Hx-FX model. Insets show the enlarged side views of the interacting HVR5 loop (top) and a non-interacting HVR5 loop (bottom), demonstrating that this conformational change occurs only in the HVR5 loop of the protomer that interacts with FII.

HAdV-C6 hexons were purified similarly as described above and incubated with either FX or FII to form the respective complexes. Both HAdV-C6 datasets contained mixtures of factor-bound and factor-free hexons. Focused 3D classification showed that, in the Ad6Hx-FX dataset, 20.5% of particles were FX-free, whereas in the Ad6Hx–FII dataset, 49.6% were FII-free (Figs 3a, 4a and S2 Table). Subsequent refinement yielded reconstructions at 3.3 Å for the Ad6Hx-FX complex (Figs 3b and S11) and 3.2 Å for the Ad6Hx-FII complex (Figs 4b and S12).

Similar to the Ad5-factor complexes, the Gla domains of FX and FII insert into a hydrophobic pocket of HAdV-C6 hexon created by the three HVR7 loops at the base of the hexon cavity, and a single HVR5 loop at the entrance of the cavity provides additional stabilizing contacts (Figs 3b-3c and 4b-4c, S4-S5 Tables). To assess whether factor binding induces structural changes in the hexon, we refined the factor-free HAdV-C6 hexon (separated by 3D classification from the HAdV-C6/FX dataset) to 3.5 Å resolution (S13 Fig). In contrast, the factor-bound complexes revealed a distinct remodeling of the HVR5 loop upon the binding of FX or FII to HAdV-C6 hexon (Figs 3d and 4d). While the HVR5 loop in the factor-free hexon adopts a flexible conformation that extends into the hexon cavity, it folds into a short α-helix in the factor-bound complexes widening the cavity entrance (Figs 3d and 4d). This conformational change is restricted to one of the HVR5 loops that directly contacts the bound factor, while the remaining two HVR5 loops are unchanged (Figs 3d and 4d). Aside from this localized rearrangement in HVR5, the rest of the hexon remains structurally conserved between the factor-free and factor-bound states (S14 Fig).

### Co-incubation of hexons with both FX and FII at their relative physiological abundance

In the bloodstream, multiple coagulation factors are present simultaneously, with FII occurring at approximately tenfold higher concentrations than FX. To assess factor binding preferences under these conditions, we incubated HAdV-C5 and HAdV-C6 hexon trimers with both FX and FII at a molar ratio of 1:2:20 (hexon: FX: FII). This experimental design maintains the same hexon: FX ratio used in the single-factor experiments while introducing FII in excess to reflect its higher physiological abundance.

**Fig 5:**
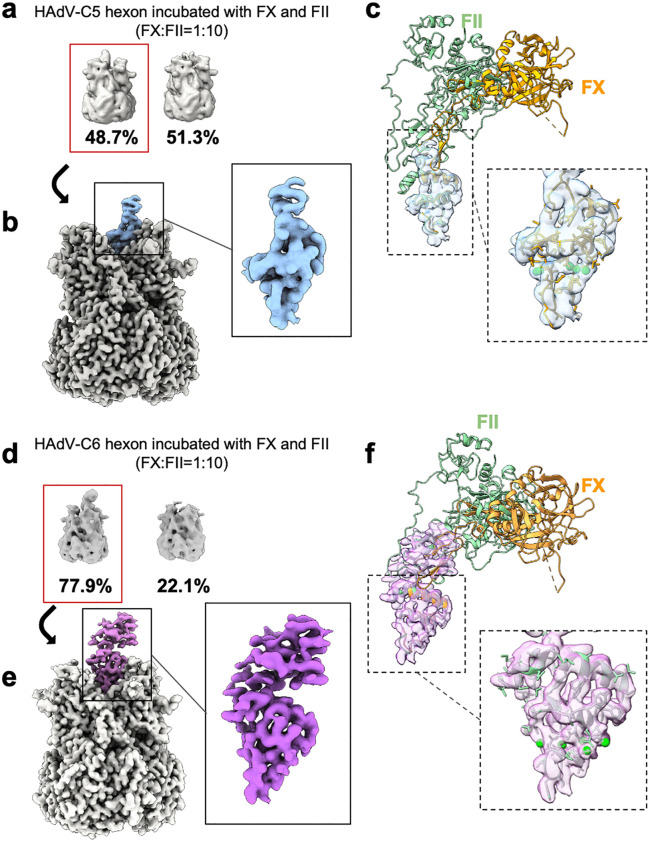
Cryo-EM structures of HAdV-C5 and HAdV-C6 hexons co-incubated with FX and FII reveals serotype-specific factor preference. **a)** Results from the focused 3D classification of the dataset of HAdV-C5 hexons co-incubated with FX and FII at a 1:10 molar ratio (FX: FII). The two major cavity occupied classes contain 48.7% and 51.3% of particles, respectively. **b)** The density map of the HAdV-C5 hexon-factor complex at 2.9 Å resolution. The inset shows the density of the bound factor cropped from the density map of the complex. **c**) The fit of both FX (orange) and FII (green) models into the cropped factor density. Inset shows that FX provides a superior fit, with the side chains aligning cleanly into the density, suggesting that HAdV-C5 hexon preferentially engages FX over FII (Q-score: 0.38 vs 0.18; map correlation: 0.57 vs 0.45). **d)** Results from the focused 3D classification of the dataset of HAdV-C6 hexons co-incubated with FX and FII at a 1:10 molar ratio (FX: FII). The major class (77.9%) shows strong factor density in the hexon cavity. **e)** The density map of the HAdV-C6 hexon-factor complex also at 2.9 Å resolution. The inset shows the density of the bound factor cropped from the density map of the complex. **f)** The fit of both FX (orange) and FII (green) models into the cropped factor density. In contrast to HAdV-C5, the cropped density from the Ad6-factor complex aligns more closely with FII, implying that HAdV-C6 hexon preferentially binds FII under conditions approximating relative abundance (Q-score: 0.21 vs 0.18; map correlation: 0.54 vs 0.53). Model assignment is supported by both visual agreement and quantitative model-map metrics (Q-score and map correlation; S6 Table).

For HAdV-C5, 3D classification revealed two major particle populations of comparable abundance (Fig 5a). The subset displaying the most well-defined factor density was refined to 2.9 Å resolution (Figs 5b and S15). Visual inspection of the density within the hexon cavity showed a closer correspondence to the FX Gla domain than to that of FII (Fig 5c). To further quantify this observation, we evaluated model-map agreement using Q-score and map-correlation metrics. The FX Gla domain exhibited substantially higher agreement with the density compared to the FII Gla domain (Q-score: 0.38 vs 0.18; map correlation: 0.57 vs 0.45), supporting preferential engagement of FX despite the excess of FII (Fig 5c and S6 Table).

In contrast, the HAdV-C6 dataset was dominated by a single major class (~78%) with clear factor density (Fig 5d), which was also refined to 2.9 Å resolution (Figs 5e and S16). In this case, the density was more consistent with the FII Gla domain than with FX. However, the quantitative analysis showed a slightly better correlation metrics for FII compared to FX (Q-score: 0.21 vs 0.18; map correlation: 0.54 vs 0.53), supporting preferential engagement of FII under the same conditions (Fig 5f and S6 Table). Together, these results indicate that factor selectivity is governed by different determinants in HAdV-C5 and HAdV-C6 hexons. For HAdV-C6, where the difference in binding affinities between FX and FII appears limited, increased ligand availability may tilt the factor occupancy towards FII; by contrast, HAdV-C5 retains a strong preference for FX even in the presence of excess FII, indicating that intrinsic binding affinity is the dominant determinant of factor selectivity.

### Structural basis for the lack of coagulation-factor binding to HAdV-D26 hexon

Previous studies have shown that HAdV-D26 does not bind FX [29], and our previous pull-down assays confirmed this result while also suggesting a weak association with FII [31]. To investigate the structural basis for this minimal engagement, we purified HAdV-D26 hexons using the same procedure applied to HAdV-C5 and HAdV-C6 and incubated them with FII at a 1:2 hexon-factor ratio. In contrast to HAdV-C5 and HAdV-C6, HAdV-D26 hexons exhibited severe aggregation and strong preferred orientation, preventing high-resolution reconstruction (S17a Fig). However, the resulting low-resolution maps showed no detectable density corresponding to bound FII in the hexon cavity (S17b Fig).

**Fig 6:**
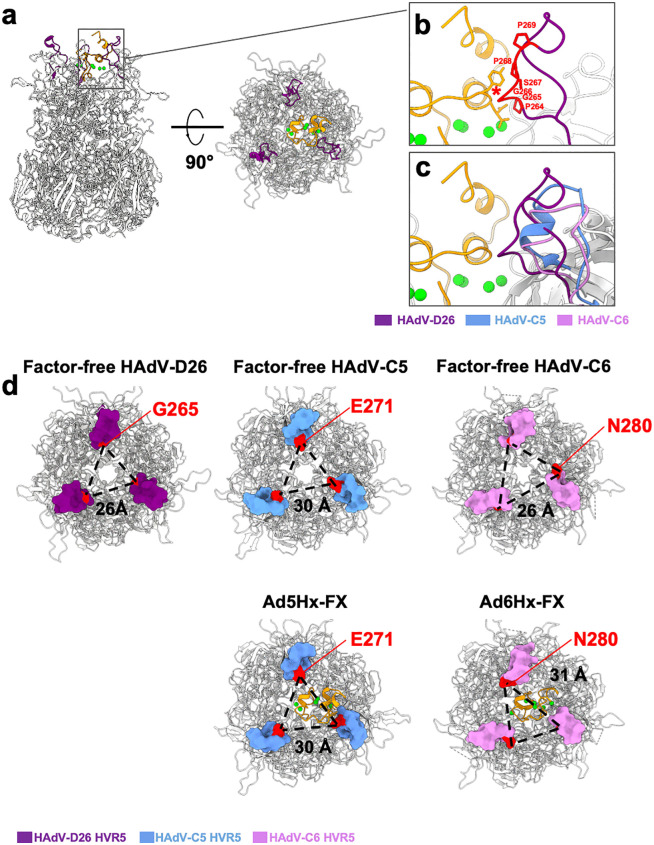
Structural basis for the inability of HAdV-D26 hexon to bind coagulation factors. **a)** Potential atomic model of Ad26Hx-FX complex generated by superimposing the HAdV-D26 hexon (PDB: 7TAU) onto the Ad5Hx-FX structure from the current study. HAdV-C5 hexon is not displayed for clarity. Majority of HAdV-D26 hexon is shown in gray except for the HVR5 loops, highlighted in dark purple, while FX is shown in orange and Ca² ⁺ ions are depicted in green. Shown on the right is a 90° rotated (top) view of the complex. **b)** Zoomed-in view of the HVR5 loop of HAdV-D26 hexon (dark purple), with the 3-residue (glycine–glycine–serine) insertion flanked by the proline residues are shown in red, that clashes with the Gla-domain (orange). The site of the steric clash is identified by an asterisk. **c)** Zoomed-in view of the structural superposition of the corresponding HVR5 regions in the hexons of HAdV-C5 (blue), HAdV-C6 (light purple), and HAdV-D26 (dark purple), highlighting the differences near the HVR5 interacting site. **d.** Quantitative measurement of hexon cavity entrance opening across different adenovirus serotypes. Distances were measured between equivalent residues identified by the structural superposition. In the factor-free state, HAdV-D26 and HAdV-C6 hexons exhibit similarly narrow cavity openings, both smaller than that of HAdV-C5. Upon factor binding (FX shown as an example), the cavity entrance of HAdV-C5 remains unchanged, whereas the HAdV-C6 hexon undergoes HVR5 rearrangement that widens the cavity opening.

To further investigate why HAdV-D26 fails to accommodate coagulation factors, we generated a pseudo-model of an Ad26Hx-FX complex by superimposing the HAdV-D26 hexon structure (PDB: 7TAU) onto our Ad5Hx-FX complex (Fig 6a). This analysis revealed a three-residue insertion (residues 265–267) within the HAdV-D26 HVR5 loop facing the hexon cavity entrance (Fig 6b). The inserted glycine–glycine–serine segment is flanked by proline residues, which likely restrict the conformational flexibility of HVR5, resulting in a narrowed cavity entrance. We hypothesize that this structural constraint would likely sterically preclude insertion of coagulation factors into the HAdV-D26 hexon cavity (Fig 6b). Notably, proline residues are absent at the corresponding positions in the HVR5 loops of HAdV-C5 and HAdV-C6 hexons (Figs 6c and S18). To quantify the extent of this restriction, we measured the size of the cavity entrance among different serotypes using equivalent residues selected based on structural alignment of the Gla-hexon interface. In the factor-free state, HAdV-D26 and HAdV-C6 hexons exhibit similarly narrow cavity openings, both smaller than that of HAdV-C5 (Fig 6d). Upon factor engagement, HAdV-C6 expands its cavity entrance through HVR5 rearrangement, whereas the entrance of the HAdV-D26 hexon is likely to remain constricted due to the inflexible HVR5 insertion (Fig 6b-6d). Furthermore, as opposed to HAdV-C5 and HAdV-C6 hexons that display a larger hydrophobic patch formed by 9 hydrophobic residues at the bottom of the cavity, the equivalent region in HAdV-D26 contains only three hydrophobic residues, one (Y458) from each hexon monomer (S19 Fig). This reduced hydrophobic interface may be insufficient to stably anchor the Gla domains of coagulation factors. Together, the steric constraints at the cavity entrance and the diminished hydrophobic interactions at the cavity base provide a potential structural explanation for the inability of HAdV-D26 hexon to bind FX and for its markedly weaker interaction with FII.

## Discussion

Coagulation factor binding plays a central role in modulating adenovirus interactions with host cells. In particular, FX has been shown to bridge adenovirus particles to hepatocytes by interacting with cell surface heparan sulfate proteoglycans, thereby promoting liver tropism following systemic administration [3,6,38]. In addition, association with coagulation factors has been shown to shield viral capsid from neutralizing antibodies and components of the innate immune system, contributing to immune evasion of C-type HAdVs [16].

Within this context, the structural differences observed between HAdV-C5, HAdV-C6, and HAdV-D26 hexons have important functional implications. The ability of HAdV-C5 hexon to engage FX efficiently is consistent with its strong dependence on FX for liver targeting, whereas the distinct engagement behavior of HAdV-C6 may reflect alternative mechanisms of host interaction or reduced reliance on FX-mediated pathways. Conversely, the structural constraints observed in HAdV-D26 hexon, which limit coagulation factor engagement, are consistent with previous observations that species D adenoviruses exhibit reduced coagulation factor dependence and distinct biodistribution profiles.

Across all Ad serotypes that bind coagulation factors, FX and FII appear to use a conserved mechanism to engage the hexon trimer, and the same mechanism may extend to other Gla domain-containing coagulation factors (e.g., FVIII and FIX). The Gla domains of the factors insert into the central hexon cavity, where a hydrophobic pocket at the base of the cavity formed by three HVR7 loops, anchors the exposed phenylalanine residue (F4) of the Gla domain through π-stacking and hydrophobic interactions. Within the cavity, the Gla domain may maintain rotational flexibility until the γ-carboxyglutamic acid groups encounter one of the HVR5 loops at the cavity entrance, which acts as a latch that restricts the factor into a single, stable orientation. Consistent with this mechanism, only one coagulation factor molecule is accommodated per hexon trimer. The high degree of sequence and structural conservation between the Gla domains of FX and FII, together with the nearly identical sets of hexon-contacting residues, further supports a shared binding mechanism. The Ca² ⁺ ions in the Gla domain, which were modeled based on expected coordination geometry from prior structural information, have been reported to play essential role in stabilizing hexon-factor engagement [15,29].

Despite their similar mode of binding, FX and FII do not engage each serotype equally. In HAdV-C5, FX is clearly preferred over FII: only ~22% of particles in the HAdV-C5 dataset contained bound FII compared to almost 100% containing FX. Even when FX and FII were present simultaneously at a relative abundance of 1: 10, HAdV-C5 hexon preferentially bound FX, indicating that intrinsic affinity rather than factor abundance dictates specificity. In contrast, HAdV-C6 showed the opposite engagement pattern under these competitive conditions, with the predominant density consistent with FII binding. These findings agree with previous SPR measurements in which HAdV-C5 binds FX more strongly than FII (K_D_ ≈ 0.74 nM vs 39 nM), while HAdV-C6 appears to bind FII with modest affinity over FX (K_D_ ≈ 2.7 nM vs 28 nM) [14].

Analysis of hexon conformational changes upon factor binding revealed no substantial structural rearrangements in the HAdV-C5 hexon, whereas a pronounced reorganization of the interacting HVR5 loop was observed in the HAdV-C6 hexon. In the factor-free HAdV-C6 hexon, the portion of HVR5 facing the cavity adopts an extended conformation. However, upon engagement with both the factors, this segment refolds into a short α-helix, thereby widening the cavity entrance. This localized, inducible conformational change likely enables HAdV-C6 hexon to accommodate coagulation factors through a flexible, locally adaptive binding interface. In our previous work, we proposed that differences in the structural characteristics of factors may influence how FX and FII engage the hexon cavity [31]. The present study suggests that the wider cavity entrance of HAdV-C5 hexon may readily allow the Gla domain to gain easy access to the hydrophobic patch at the bottom of the cavity. Moreover, the flexible FX may also allow the Gla domain to remain rotationally mobile after initial capture by this hydrophobic patch, thereby increasing the opportunity to explore and form stabilizing contacts with the HVR5 loop. In contrast, the bulkier and more constrained architecture of FII may limit such reorientation, reducing the likelihood of forming more stable interactions. In HAdV-C6, the factor-free hexon adopts a more restricted cavity entrance, with HVR5 partially blocking easy access to the hydrophobic patch. However, initial engagement with the factors likely triggers the observed HVR5 reorganization, thereby widening the entrance to the cavity and allowing the Gla domain to engage the hydrophobic patch located at the bottom of the cavity. This model suggests that HAdV-C6 accommodates FX and FII through a locally adaptive HVR5-mediated mechanism, whereas HAdV-C5 more readily supports direct capture of the Gla domain within a preformed cavity.

In contrast to species C adenoviruses (e.g., HAdV-C5 and HAdV-C6), HAdV-D26, a species D adenovirus lacks the structural adaptability observed in HAdV-C6, despite having a hexon cavity entrance of comparable size in the factor-free state. Our structural analyses indicate that a conformationally rigid three-residue insertion (residues 265–267), flanked by proline residues within the HVR5 loop, might play a key role in sterically restricting HAdV-D26 hexons to bind to coagulation factors. In addition, the HAdV-D26 hexon forms a substantially smaller hydrophobic patch at the base of the cavity, which may be insufficient to securely anchor the Gla domain even if partial entry were possible. Notably, a similar HVR5 insertion is present in the hexons of other species D adenoviruses, such as HAdV-D45 (S20 Fig), suggesting that a rigid HVR5 loop might be a conserved structural feature among species D adenovirus hexons that precludes binding to coagulation factors.

Compared to earlier lower-resolution maps [6,24] and AdV-factor reconstructions [31], the hexon-factor complex structures presented here particularly resolve the contacting amino acid residues of the Gla domain in detail and reveal new interactions that influence binding affinities. These results highlight how subtle sequence variations and local conformational constraints within HVR5 and HVR7 loops control the ability of adenoviruses to engage host factors. These structural determinants provide a blueprint for rational engineering of hexon surfaces to modulate coagulation-factor binding, with potential implications for improving adenoviral vector targeting, reducing off-target liver transduction, and enhancing vector safety.

## Methods

### Purification of adenovirus hexons

Adenovirus (Ad) hexons were purified either by rupturing the respective Ad virions or from the soluble proteins found at the top of the CsCl gradients while purifying the Ad virions. To rupture the Ad virions, the samples were dialyzed overnight against the hexon buffer (10 mM HEPES, pH 7.2, 10 mM NaCl). Following dialysis, the samples were supplemented with 1% sodium deoxycholate and heated in a 56 °C water bath for 10 minutes, followed by three to five freeze-thaw cycles in liquid nitrogen and the 56 °C water bath. The sample was then centrifuged at 4,000g to remove debris of precipitated dsDNA and core proteins. The supernatant was dialyzed against the hexon buffer to remove any remaining sodium deoxycholate. From here onwards, the hexons from the disrupted Ad virions or soluble hexons from the CsCl bands were treated similarly. The dialyzed sample was filtered through a 0.22-micron filter prior to FPLC runs. For initial purification, a DEAE Sepharose column (HiTrap-DEAE-FF, Cytiva) was used to perform anion exchange chromatography on an AKTA go purifier. Selected fractions from various peaks were analyzed by SDS-PAGE. Fractions containing hexon protein based on molecular weight (~110 kDa) were combined and dialyzed overnight in the hexon buffer. The sample was then further purified using a HiTrap Q HP column, and the fractions containing hexon protein were confirmed by SDS-PAGE. The hexon protein fractions were then concentrated using a 30kDa MWCO centrifugal filter (Amicon) and the sample’s homogeneity was assessed by negative stain electron microscopy.

### Negative stain electron microscopy

To assess the homogeneity of the adenovirus hexons, 8 µL of the purified hexon protein was applied to a carbon-coated, glow-discharged copper grid (EMS) and stained with 1% uranyl formate (EMS). The grid was then air-dried and examined using a Tecnai Spirit BioTwin Transmission Electron Microscope (Thermo Fisher) (S21 Fig).

### Formation of hexon-factor complex

The purified hexon protein was dialyzed into DPBS buffer (137 mM NaCl, 2.7 mM KCl, 10 mM phosphate buffer, pH 7.4) supplemented with 0.9 mM CaCl_2_ and 0.5 mM MgCl_2_. The hexon-factor complex was formed by mixing hexon with either Factor X or Factor II at a 1:2 ratio (hexon trimer: factor) and incubating overnight at 4°C. For co-incubation experiments, hexon trimers were incubated with FX and FII simultaneously at a molar ratio of 1:2:20 (hexon:FX:FII).

### Cryo-EM grid preparation and data collection

For each complex, 4 µL of sample was applied to a glow-discharged holey carbon grid (Quantifoil, 300 mesh, R1.2/1.3, EMS) and vitrified using a Vitrobot (Thermo Fisher Scientific). Each grid was incubated for 30 seconds after sample application, blotted for 5 seconds with blot force 0 (100% humidity, 4°C), then plunged into liquid ethane. The grids are stored in liquid nitrogen until data collection. Cryo-EM single-particle data were collected using a Titan Krios G3i microscope (Thermo Fisher Scientific) operated at 300 kV, equipped with a Gatan Imaging Filter (GIF) and a 20-eV energy-selective slit. Automated data collection was performed using EPU software (Thermo Fisher Scientific) with parameters detailed in Table 1. Each movie comprised 40 frames with a total electron dose of ~51 e-/Å², at a pixel size of 1.1 Å or 0.85 Å. The nominal defocus during data collection ranged from -0.8 µm to -2.4 µm.

**Table 1:** Cryo-EM data collection statistics and reconstruction metrics for hexon-factor complexes.

	Ad5Hx-FX	Ad5Hx-FII	Ad6Hx-FX	Ad6Hx-FII	Ad5Hx-FX/FII	Ad6Hx-FX/FII	Ad26Hx-FII
**Data collection**							
**Microscope**	Krios G3i	Krios G3i	Krios G3i	Krios G3i	Krios G3i	Krios G3i	Glacios 2
**Detector**	Gatan K3	Gatan K3	Gatan K3	Gatan K3	Gatan K3	Gatan K3	Falcon 4i
**Voltage (kV)**	300	300	300	300	300	300	200
**Exposure time (s)**	3.5	3.5	2.5	2.5	2	2	5.45
**Total dose (e**^**-**^**/A**^**2**^)	51	51	50	50	52	50	46
**Symmetry imposed**	C3 relaxed	C3 relaxed	C3 relaxed	C3 relaxed	C3 relaxed	C3 relaxed	C3 relaxed
**No. of Movies**	5192	4048	3510	7275	9482	1638	16,594
**Particles selected**	715,202	252,960	280,503	1,099,039	2,075,577	224,594	24,948
**Particles included in final reconstruction**	96,107	97,601	68,552	91,729	54,285	19,920	10,460
**Reconstruction**							
**Pixel size (Å)**	1.1	1.1	0.85	0.85	0.85	0.85	0.88
**Defocus range (μm)**	0.8-2.4	0.8-2.4	0.8-2.4	0.8-2.4	0.8-2.4	0.8-2.4	0.8-2.4
**Reference-based motion correction**	Yes	Yes	Yes	Yes	Yes	Yes	Yes
**Beam tilt correction**	No	No	No	No	Yes	Yes	No
**Resolution (Å)** **FSC cut-off: 0.143**	3.18	3.17	3.26	3.22	2.96	2.92	11.23

For Ad26Hx-FII complex, cryo-EM data were collected on a Glacios 2 Cryo-TEM (Thermo Fisher Scientific) operated at 200 kV. Detailed data collection parameters are summarized in Table 1.

### Data processing and reconstruction

The hexon-factor datasets were processed using CryoSPARC v4.4.1 [30]. An overview of the general workflow is shown in S2 Fig. Briefly, movie frames were imported into CryoSPARC and aligned using Patch Motion Correction [39] or MotionCor2 [40] to correct for drift and beam-induced motion. Contrast transfer function (CTF) analysis was performed using Patch CTF Estimation [41]. Particles were initially selected using the blob picker, with a diameter range of 90–120 Å, and then refined through reference-free 2D classification [30]. Well-defined 2D classes representing top and side views of hexon particles were selected and used as templates for a second round of template-based particle picking [42]. The particles were extracted with a 256-pixel² box (pixel size 1.1 Å) and binned by a factor of 4 (pixel size 4.4 Å) for reference-free 2D classification. Well-defined hexon classes were selected for Ab initio model generation and heterogeneous refinement [30] to remove poor-quality particles. The refined particles were re-extracted in bin 1 (pixel size 1.1 Å) with a 256-pixel² box size and subjected to non-uniform refinement with C3 symmetry relaxation. To further separate hexon-factor complexes from the factor-free hexons, focused 3D classification [43] was performed using a spherical mask over the hexon cavity. Classes with prominent factor protrusions were selected and combined for another round of non-uniform refinement with C3 symmetry relaxation, followed by reference-based motion correction and a final round of 3D refinement. Post-processing and map sharpening were performed using DeepEMhancer [44]. Fourier Shell Correlation (FSC) plots, local resolution maps, and angular distributions of the particles are shown in Supplementary Figures.

For the Ad5Hx-FII complex, where preferred orientation was observed, two additional datasets were collected: one using graphene-oxide-coated grids and the other using Quantifoil grids but tilted to 30° during data collection. After CTF estimation, all three datasets were combined, and the subsequent processing followed the same steps as for the other samples.

### Model building and refinement

Initial models of the hexon-factor complex were generated using Alpha Fold-predicted models of the coagulation factors [45] and PDB models of hexon (HAdV-C5: PDB 1P30; HAdV-C6: determined in our lab). Glutamic acid residues in the N-terminal Gla domain of FX and FII were manually replaced with Gla residues to account for the post-translational modification as described previously [31]. Coordinating Ca² ⁺ ions were adapted from PDB: 1IOD [27]. The initial models were docked into the sharpened maps using ChimeraX [46, 47], followed by adjustment in COOT [34] and real-space refinement in PHENIX [33]. Refinement statistics and model details are provided in Table 2. The interactions between the Gla domains and the hexon monomers were calculated using the CONTACT program in CCP4 [35], with a 4.0 Å distance cutoff. A full list of contacting residues is provided in S1,S3,S5 Tables.

**Table 2:** Model refinement statistics for hexon-factor complexes.

	Ad5Hx-FX	Ad5Hx-FII	Ad6Hx-FX	Ad6Hx-FII	Factor-free Ad5Hx	Factor-free Ad6Hx
PDB ID	13ES	13 CM	13EU	13ER	13DJ	13DM
EMDB ID	EMD-77025	EMD-76963	EMD-77027	EMD-77024	EMD-76991	EMD-76994
**Model composition and refinement statistics**						
Number of chains	4	4	4	4	3	3
Number of atoms	22421	22550	22259	22474	21984	21851
Number of protein residues	2789	2809	2779	2815	2742	2736
Clash score	5.47	6.05	6.25	6.52	5.25	4.53
Rotamer outliers	3.59%	5.58%	3.79%	5.49%	3.43%	3.29%
MolProbity score	2.21	2.33	2.21	2.39	2.16	2.07
EMRinger Score	4.21	3.66	3.74	4.08	3.98	4.24
Map-Model cc (main chain)	0.64	0.75	0.69	0.75	0.66	0.74
Map-Model cc (side chain)	0.63	0.75	0.69	0.75	0.64	0.73
**Ramachandran statistics**						
Favored	91.89	93.36	93.55	92.46	92.33	92.92
Allowed	8.08	6.57	6.45	7.32	7.64	7.04
Outliers	0.04	0.07	0	0.22	0.04	0.04
**R.M.S. deviations**						
Bond Lengths (a)	0.006	0.004	0.003	0.005	0.006	0.004
Bond angles (°)	0.646	0.643	0.545	0.703	0.664	0.624

## Supporting information

S1 Fig A representative Cryo-EM data processing workflow employed for the structure determination of hexon-factor complexes using CryoSPARC. A total of 5,192 movies were collected for Ad5Hx-FX complex. After initial 2D classification, 712,699 particles were selected and subjected to heterogeneous refinement. Well-defined classes were selected and further refined through homogeneous refinement. A focused 3D classification using a spherical mask over the hexon cavity was performed to distinguish factor-bound particles. A subset of 96,107 particles exhibiting bulky factor density was selected for final homogeneous refinement. Arrows indicate to the tail-like density visible in the 2D classes.
(DOCX)

S2 Fig Resolution estimation and assessment of map quality of the Ad5Hx-FX complex. **a, d**, Local resolution maps of the Ad5Hx-FX density map before **(a)** and after **(d)** focused 3D classification. Resolution gradient spans from 2.0 Å (blue) to 4.0 Å (red). **b, e**, Angular distribution plots showing particle orientations before **(b)** and after **(e)** focused 3D classification. **c, f,** Gold-standard Fourier shell correlation (GSFSC) curves of the Ad5Hx-FX complex before **(c)** and after **(f)** focused 3D classification, with resolution estimated at the FSC = 0.143 threshold.
(DOCX)

S3 Fig AlphaFold2-predicted structure of human coagulation factosr FX and FII, colored by pLDDT confidence. **a,** AlphaFold2-predicted structure of FX. **b**, AlphaFold2-predicted structure of FII. Both models are shown as ribbon diagrams colored by per-residue pLDDT confidence scores (blue, high confidence; cyan, intermediate confidence; yellow, low confidence; orange, very low confidence). pLDDT values for the AlphaFold models of FX and FII are 80.7 and 85.9 respectively.
(DOCX)

S4 Fig Structural comparison of Ad5Hx-FX models. Superposition of the Ad5Hx-FX complex determined in this study (blue hexon and orange FX-Gla) with the previously reported Ad5-FX model (PDB 9CLI; purple) shows high overall similarity, with an RMSD of 0.51 Å. The inset highlights the FX-Gla domain, illustrating the subtle positional shift between the two models.
(DOCX)

S5 Fig Orientation distributions of particles in the un-tilted (initial), tilted and graphene-coated datasets of Ad5Hx-FII. **a**, Representative 2D class averages from the un-tilted (initial) Ad5Hx-FII dataset showing strong preferred orientation, with most particles adopting top or bottom views. **b**, 2D class averages after incorporating an additional 30-degree tilted dataset and images collected on graphene-coated grids, demonstrating improved angular distribution and reduced preferred orientation.
(DOCX)

S6 Fig Resolution estimation and assessment of map quality of the Ad5Hx-FII complex. **a, d**, Local resolution maps of the Ad5Hx–FII reconstruction before **(a)** and after **(d)** focused 3D classification. Resolution gradient spans from 2.0 Å (blue) to 4.0 Å (red). **b, e**, Angular distribution plots showing particle orientations before **(b)** and after **(e)** focused 3D classification. **c, f,** Gold-standard Fourier shell correlation (GSFSC) curves of the Ad5Hx-FII complex before **(c)** and after **(f)** focused 3D classification, with resolution estimated at the FSC = 0.143 threshold.
(DOCX)

S7 Fig Sequence conservation of the Gla domains from human coagulation factor X (FX) and factor II (FII). **a,** Amino acid sequence alignment of the γ-carboxyglutamic acid (Gla) domains from human FX and FII, which are responsible for calcium-dependent interactions with adenovirus hexon proteins. Identical residues are highlighted in blue. **b**, Structural superposition of the FX and FII Gla domains, showing their highly similar overall folds despite minor local differences. This strong sequence and structural conservation is consistent with a shared mode of interaction with adenovirus hexon proteins.
(DOCX)

S8 Fig 3D reconstruction of factor free HAdV-C5 hexon. **a,** Cryo-EM density map of factor-free HAdV-C5 hexon. The three hexon monomers are displayed in different colors to indicate subunit boundaries. **b,** Atomic model of the factor-free HAdV-C5 hexon built from the density map, with each monomer shown in a different color corresponding to panel a. **c,** Gold-standard Fourier shell correlation (GSFSC) curve of the HAdV-C5 hexon reconstruction, with resolution estimated at the FSC = 0.143 threshold. Factor-free hexon structures were reconstructed from particle classes lacking visible density for bound coagulation factors, derived from the same datasets as the Ad5Hx-FII complexes.
(DOCX)

S9 Fig Structural comparison of factor-free and factor-bound HAdV-C5 hexon. Left, superposition of the factor-free HAdV-C5 hexon (grey), Ad5Hx-FX complex (orange), and Ad5Hx-FII complex (green), showing that the overall hexon architecture is highly conserved across all states. Right, enlarged views highlight local regions at the hexon-factor interface. **i**, HVR7 loops at the base of the hexon cavity. **ii**, HVR5 loops at the cavity entrance.
(DOCX)

S10 Fig Structural comparison of Ad5Hx-FII models. Superposition of the Ad5Hx-FII complex determined in this study (hexon in blue and FII-Gla in green) onto previously reported Ad5Hx-FII model (PDB 9CLN; grey) shows overall agreement with an RMSD of 0.78 Å. The inset highlights the Gla domain of FII, illustrating the close correspondence in its positioning between the two models.
(DOCX)

S11 Fig Resolution estimation and assessment of map quality of the Ad6Hx-FX complex. **a, d**, Local resolution maps of the Ad6Hx-FX reconstruction before **(a)** and after **(d)** focused 3D classification. Resolution gradient spans from 2.0 Å (blue) to 4.0 Å (red). **b, e**, Angular distribution plots showing particle orientations before **(b)** and after **(e)** focused 3D classification. **c, f,** Gold-standard Fourier shell correlation (GSFSC) curves of the Ad6Hx-FX complex before **(c)** and after **(f)** focused 3D classification, with resolution estimated at the FSC = 0.143 threshold.
(DOCX)

S12 Fig Resolution estimation and assessment of map quality of the Ad6Hx-FII complex. **a, d**, Local resolution maps of the Ad6Hx-FII reconstruction before **(a)** and after **(d)** focused 3D classification. Resolution gradient spans from 2.0 Å (blue) to 4.0 Å (red). **b, e**, Angular distribution plots showing particle orientations before **(b)** and after **(e)** focused 3D classification. **c, f,** Gold-standard Fourier shell correlation (GSFSC) curves of the Ad6Hx-FII complex before **(c)** and after **(f)** focused 3D classification, with resolution estimated at the FSC = 0.143 threshold.
(DOCX)

S13 Fig 3D reconstruction of factor-free HAdV-C6 hexon. **a,** Cryo-EM density map of the factor-free HAdV-C6 hexon. The three hexon monomers are displayed in different colors to indicate subunit boundaries. **b,** Atomic model of the factor-free HAdV-C6 hexon built from the density map, with each monomer shown in a different color corresponding to panel a. **c,** Gold-standard Fourier shell correlation (GSFSC) curve of the HAdV-C6 hexon reconstruction, with resolution estimated at the FSC = 0.143 threshold. Factor-free hexon structures were reconstructed from particle classes lacking visible density for bound coagulation factors, derived from the same datasets as the Ad6Hx-FX complexes.
(DOCX)

S14 Fig Structural comparison of factor-free and factor-bound HAdV-C6 hexon. Left, superposition of the factor-free HAdV-C6 hexon (grey), Ad6Hx-FX complex (orange), and Ad6Hx-FII complex (green), showing that the overall hexon trimer architecture is largely conserved across all states, except for the factor-engaged HVR5 loop, which undergoes rearrangement upon binding of either FX or FII. Right, enlarged views highlight local conformational differences at the hexon-factor interface. **i**, 90°- rotated view of the HVR7 loops at the base of the hexon cavity, which remain structurally similar between the factor-free and the factor-bound states. **ii**, HVR5 loops at the cavity entrance, illustrating localized conformational rearrangements upon FX or FII binding. These changes are restricted to the factor-engaged HVR5 loop, while the rest of the hexon structure remains unchanged.
(DOCX)

S15 Fig Resolution assessment of the HAdV-C5 hexon co-incubated with FX and FII. **a,** Local resolution map of the Ad5Hx-FX/FII complex, colored according to the resolution scale shown (2.0-4.0 Å). **b,** Gold-standard Fourier shell correlation (GSFSC) curves for the Ad5Hx-FX/FII reconstruction. The overall resolution of 2.96 Å was determined using the FSC = 0.143 criterion after mask correction.
(DOCX)

S16 Fig Resolution assessment of the HAdV-C6 hexon co-incubated with FX and FII. **a,** Local resolution map of the Ad6Hx-FX/FII complex, colored according to the resolution scale shown (2.0-4.0 Å). **b,** Gold-standard Fourier shell correlation (GSFSC) curves for the Ad6Hx-FX/FII reconstruction. The overall resolution of 2.96 Å was determined using the FSC = 0.143 criterion after mask correction.
(DOCX)

S17 Fig Preferred orientation and low-resolution reconstruction of the Ad26Hx-FII dataset. (**a**) Representative 2D class averages of the Ad26Hx-FII dataset showing pronounced preferred orientation, with most particles adopting top-view projections. (**b**) Low-resolution 3D reconstruction generated from these classes in CryoSPARC. No density corresponding to FII is observed within the hexon cavity, consistent with the notion of lack of detectable factor binding.
(DOCX)

S18 Fig Sequence alignment of HAdV-C5, HAdV-C6, and HAdV-D26 hexon proteins. Amino acid sequence alignment highlighting conserved and variable regions among HAdV-C5, HAdV-C6, and HAdV-D26 hexons. Of note, partial sequence alignment spanning hypervariable regions HVR5 and HVR7 (boxed in red), the key regions involved in coagulation factor binding are shown. Identical residues are shaded in dark blue.
(DOCX)

S19 Fig Comparison of hydrophobic residues forming the base of the hexon cavity in HAdV-D26, HAdV-C5, and HAdV-C6. Surface representations of the hexon trimers of HAdV-D26, HAdV-C5, and HAdV-C6 highlighting the hydrophobic pocket at the bottom of the central cavity. The hydrophobic patch of Ad26Hx comprises a single contributing residue (Y458) and its two symmetry mates, resulting in a markedly smaller hydrophobic patch (~8 Å across). In contrast, Ad5Hx and Ad6Hx each possess three hydrophobic residues (Ad5: I421, F458, M460; Ad6: F470, M472, I431), producing larger pockets of ~9.4 Å and ~11.5 Å, respectively. These differences in the hydrophobic pocket size and composition may contribute to the differences in the factor binding or lack of it between Ad26Hx vs Ad5Hx and Ad6Hx.
(DOCX)

S20 Fig Sequence alignment of HAdV-D26 and HAdV-D45 hexon proteins. Amino acid sequence alignment highlighting the conserved insertion flanked by Proline residues in HVR5 (boxed in red) in HAdV-D26 and HAdV-D45 hexons, both of which belong to species D adenoviruses.
(DOCX)

S21 Fig Negative-stain electron microscopy of purified hexon trimers. Representative micrograph showing a highly homogeneous population of adenovirus hexon trimers, confirming the quality and uniformity of the purified sample prior to cryo-EM analysis.
(DOCX)

S1 Table List of interacting residues between coagulation factor X (FX) and HAdV-C5 hexon. (DOCX)

S2 Table Distribution of focused 3D classification populations across all datasets. (DOCX)

S3 Table List of interacting residues between prothrombin (FII) and HAdV-C5 hexon. (DOCX)

S4 Table List of interacting residues between coagulation factor X (FX) and HAdV-C6 hexon. (DOCX)

S5 Table List of interacting residues between prothrombin (FII) and HAdV-C6 hexon. (DOCX)

S6 Table Assessment of the fit of Gla domains to the factor density in the co-incubation study. (DOCX)
