# Supplementary figures and images for: Structural requirements of blood factors binding to soluble hexon trimers with implications for adenovirus cell targeting and immune evasion
Source: PLoS Pathog. 2026 Jul 13;22(7):e1014389. doi: 10.1371/journal.ppat.1014389 (PMC13379079; doi:10.1371/journal.ppat.1014389)

# S1 Fig


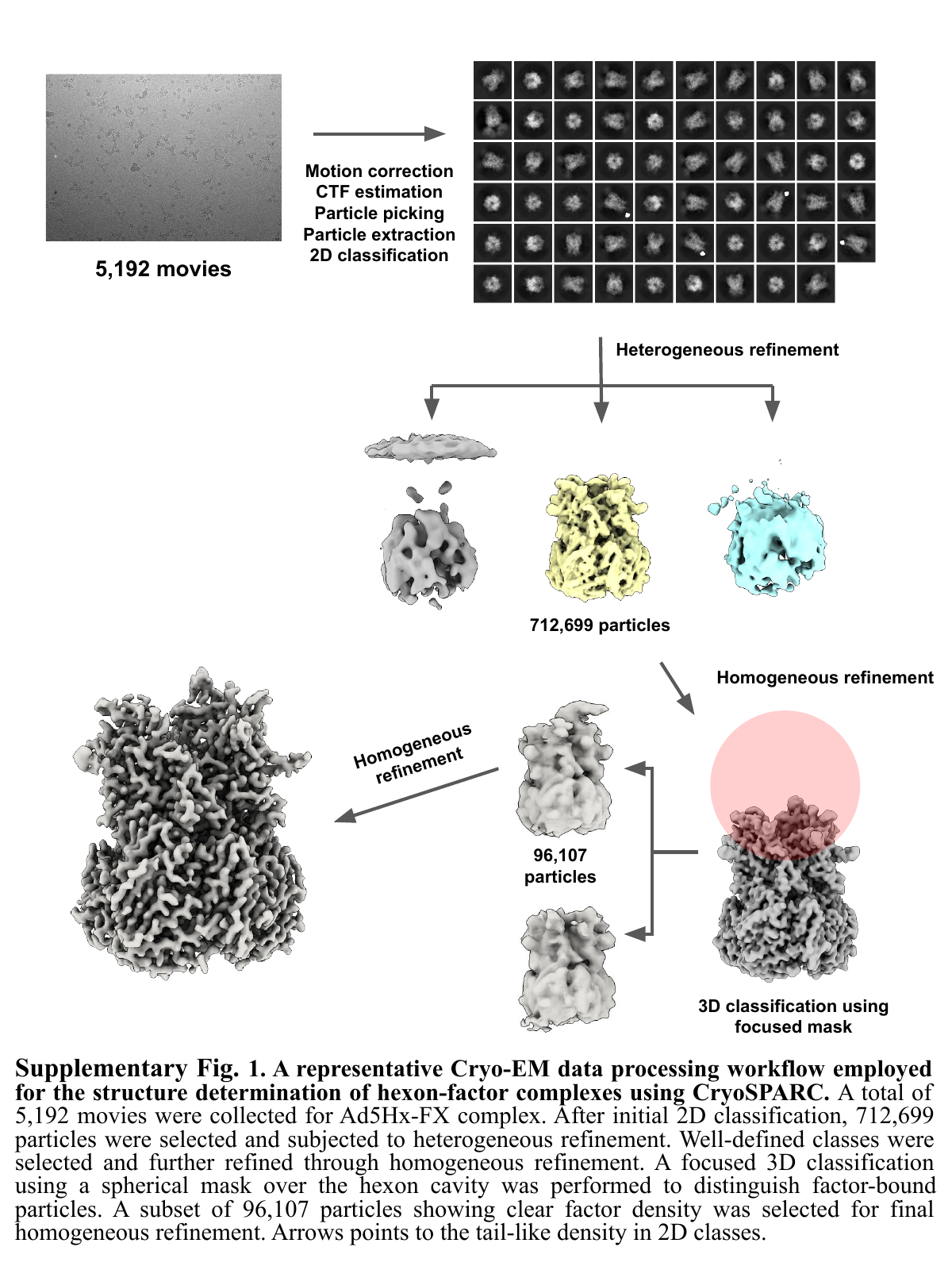

Supplement: S1 Fig — A total of 5,192 movies were collected for Ad5Hx-FX complex. After initial 2D classification, 712,699 particles were selected and subjected to heterogeneous refinement. Well-defined classes were selected and further refined through homogeneous refinement. A focused 3D classification using a spherical mask over the hexon cavity was performed to distinguish factor-bound particles. A subset of 96,107 particles exhibiting bulky factor density was selected for final homogeneous refinement. Arrows indicate to the tail-like density visible in the 2D classes. (DOCX) [file ppat.1014389.s001.docx]

# S2 Fig


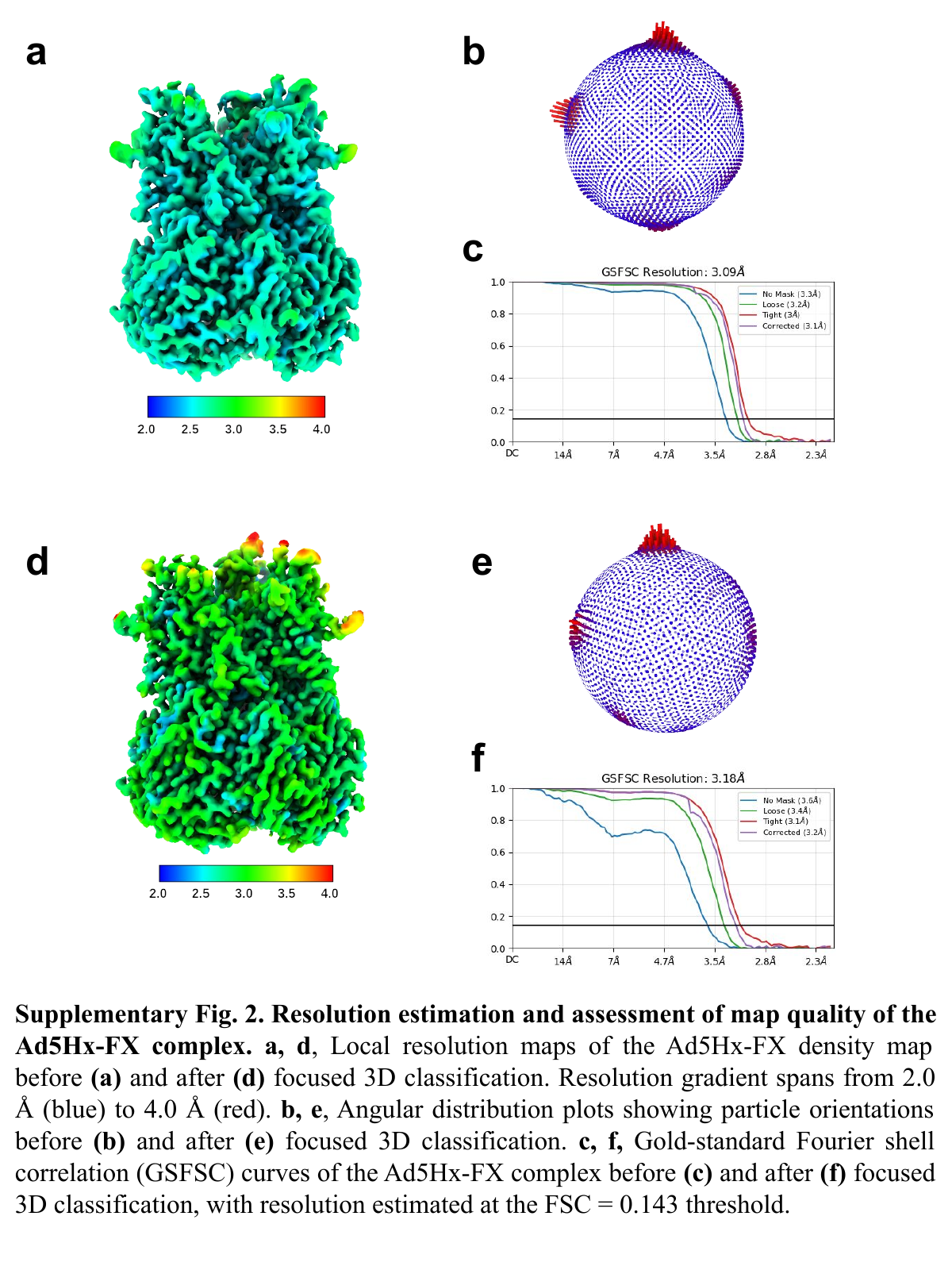

Supplement: S2 Fig — a, d, Local resolution maps of the Ad5Hx-FX density map before (a) and after (d) focused 3D classification. Resolution gradient spans from 2.0 Å (blue) to 4.0 Å (red). b, e, Angular distribution plots showing particle orientations before (b) and after (e) focused 3D classification. c, f, Gold-standard Fourier shell correlation (GSFSC) curves of the Ad5Hx-FX complex before (c) and after (f) focused 3D classification, with resolution estimated at the FSC = 0.143 threshold. (DOCX) [file ppat.1014389.s002.docx]

# S3 Fig


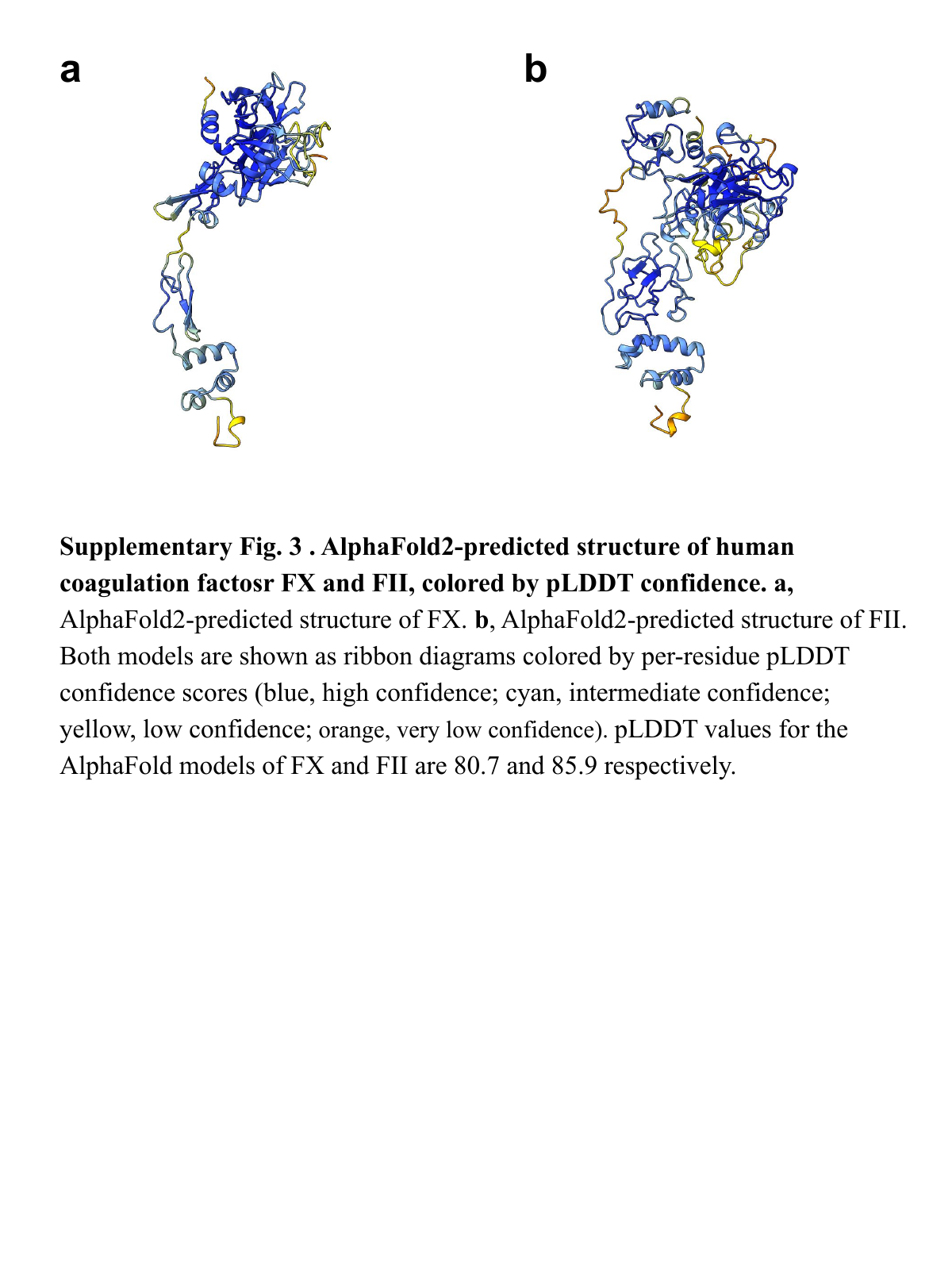

Supplement: S3 Fig — a, AlphaFold2-predicted structure of FX. b, AlphaFold2-predicted structure of FII. Both models are shown as ribbon diagrams colored by per-residue pLDDT confidence scores (blue, high confidence; cyan, intermediate confidence; yellow, low confidence; orange, very low confidence). pLDDT values for the AlphaFold models of FX and FII are 80.7 and 85.9 respectively. (DOCX) [file ppat.1014389.s003.docx]

# S4 Fig


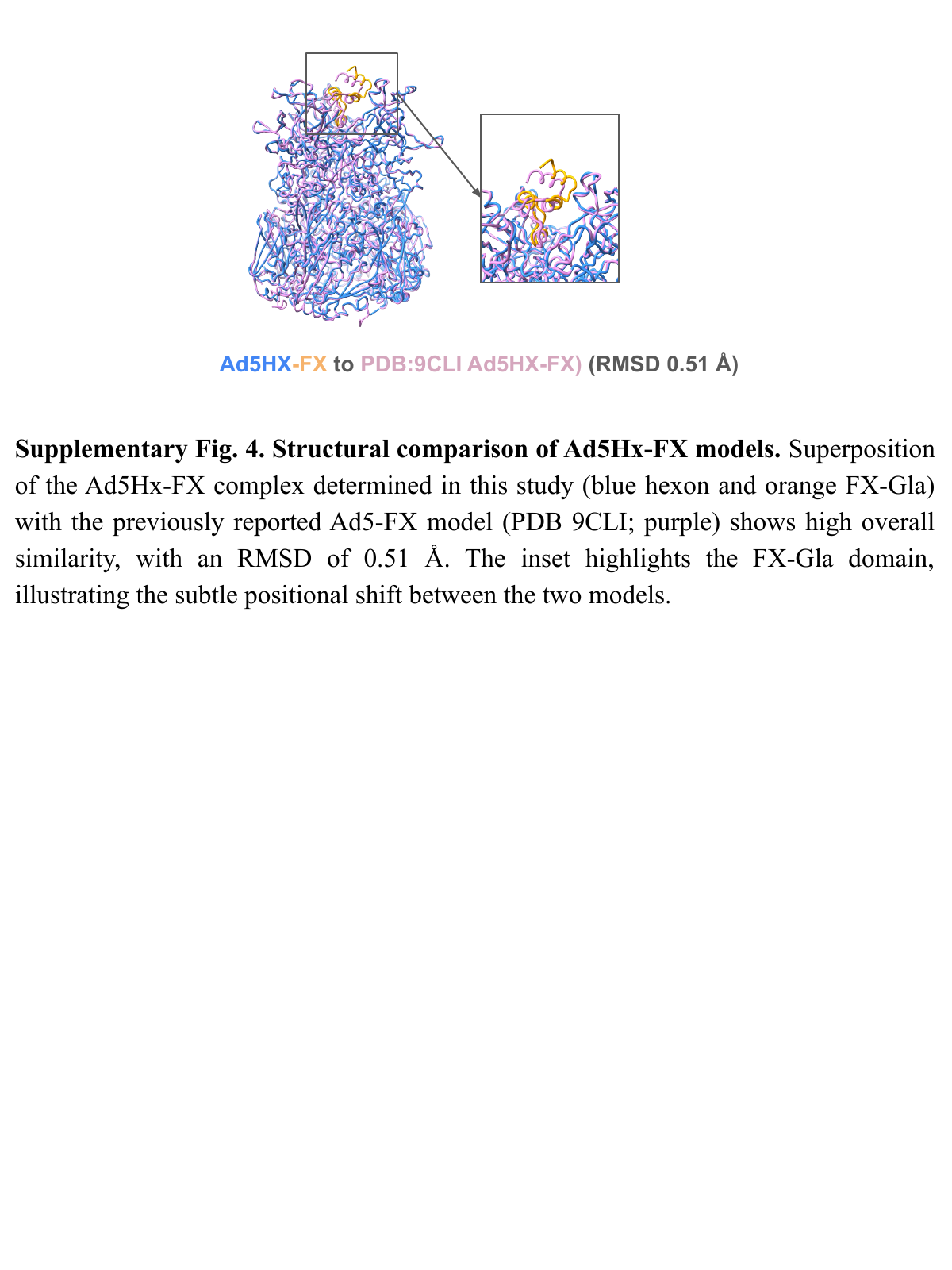

Supplement: S4 Fig — Superposition of the Ad5Hx-FX complex determined in this study (blue hexon and orange FX-Gla) with the previously reported Ad5-FX model (PDB 9CLI; purple) shows high overall similarity, with an RMSD of 0.51 Å. The inset highlights the FX-Gla domain, illustrating the subtle positional shift between the two models. (DOCX) [file ppat.1014389.s004.docx]

# S5 Fig


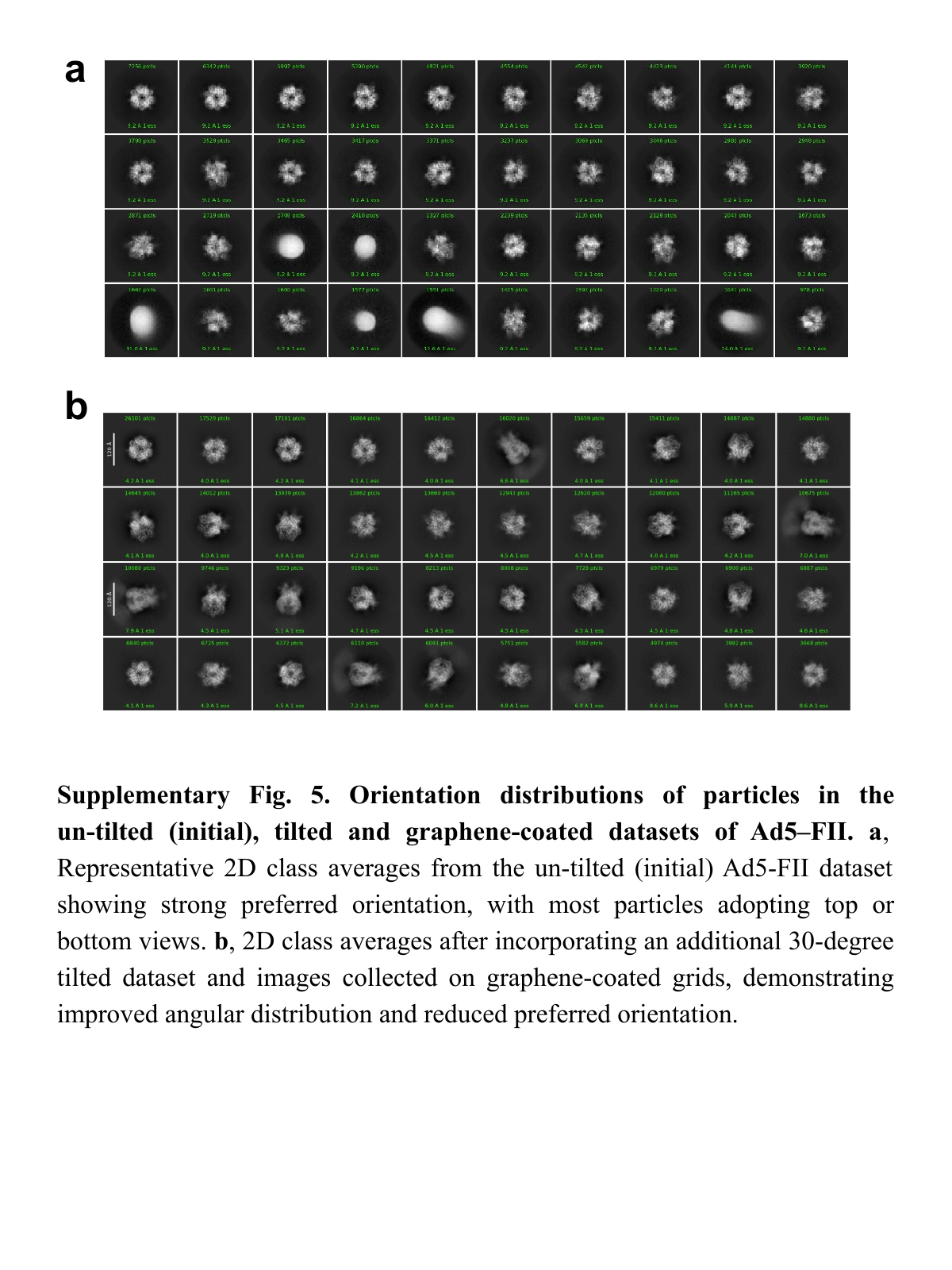

Supplement: S5 Fig — a, Representative 2D class averages from the un-tilted (initial) Ad5Hx-FII dataset showing strong preferred orientation, with most particles adopting top or bottom views. b, 2D class averages after incorporating an additional 30-degree tilted dataset and images collected on graphene-coated grids, demonstrating improved angular distribution and reduced preferred orientation. (DOCX) [file ppat.1014389.s005.docx]

# S6 Fig


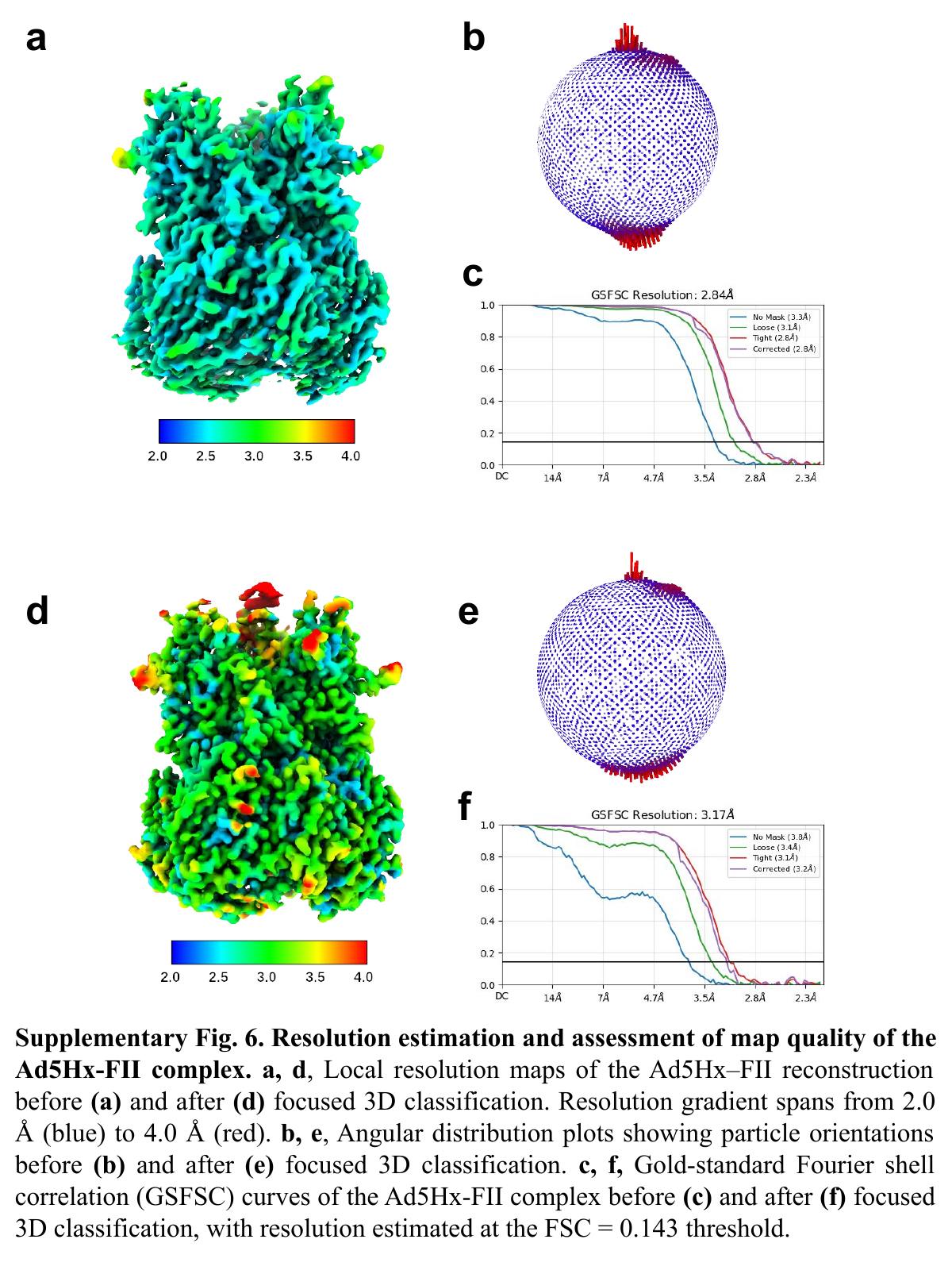

Supplement: S6 Fig — a, d, Local resolution maps of the Ad5Hx–FII reconstruction before (a) and after (d) focused 3D classification. Resolution gradient spans from 2.0 Å (blue) to 4.0 Å (red). b, e, Angular distribution plots showing particle orientations before (b) and after (e) focused 3D classification. c, f, Gold-standard Fourier shell correlation (GSFSC) curves of the Ad5Hx-FII complex before (c) and after (f) focused 3D classification, with resolution estimated at the FSC = 0.143 threshold. (DOCX) [file ppat.1014389.s006.docx]

# S7 Fig


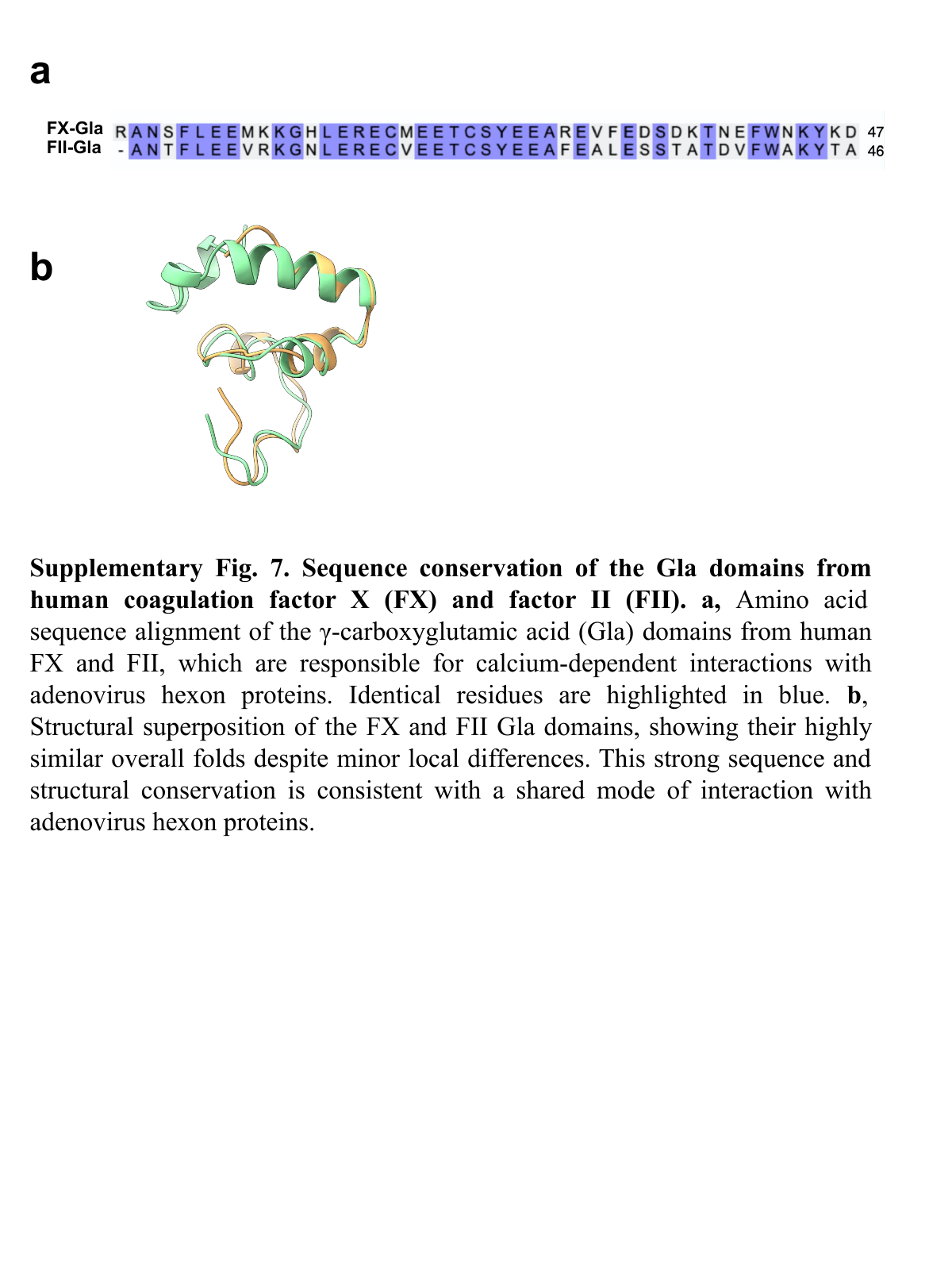

Supplement: S7 Fig — a, Amino acid sequence alignment of the γ-carboxyglutamic acid (Gla) domains from human FX and FII, which are responsible for calcium-dependent interactions with adenovirus hexon proteins. Identical residues are highlighted in blue. b, Structural superposition of the FX and FII Gla domains, showing their highly similar overall folds despite minor local differences. This strong sequence and structural conservation is consistent with a shared mode of interaction with adenovirus hexon proteins. (DOCX) [file ppat.1014389.s007.docx]

# S8 Fig


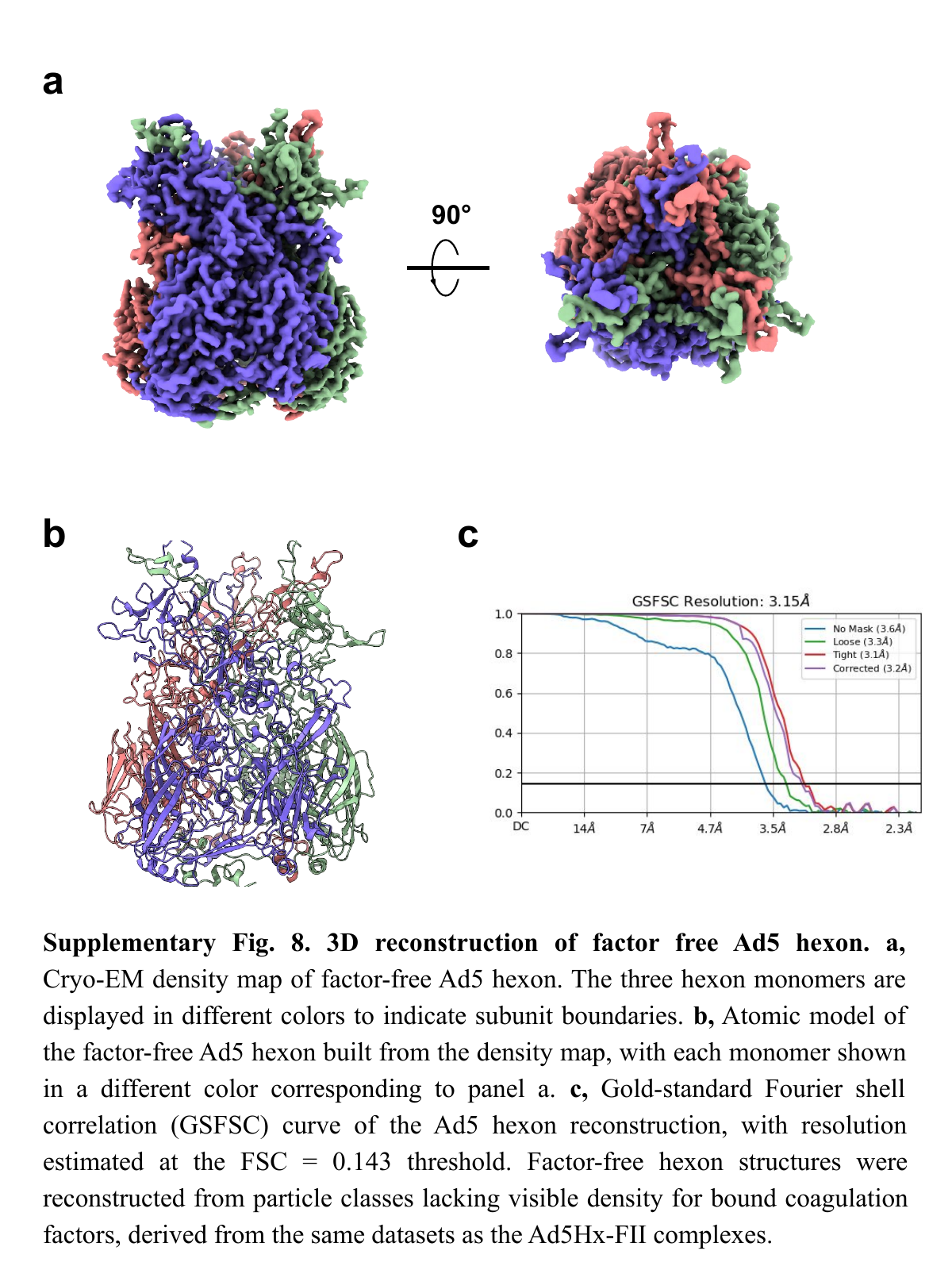

Supplement: S8 Fig — a, Cryo-EM density map of factor-free HAdV-C5 hexon. The three hexon monomers are displayed in different colors to indicate subunit boundaries. b, Atomic model of the factor-free HAdV-C5 hexon built from the density map, with each monomer shown in a different color corresponding to panel a. c, Gold-standard Fourier shell correlation (GSFSC) curve of the HAdV-C5 hexon reconstruction, with resolution estimated at the FSC = 0.143 threshold. Factor-free hexon structures were reconstructed from particle classes lacking visible density for bound coagulation factors, derived from the same datasets as the Ad5Hx-FII complexes. (DOCX) [file ppat.1014389.s008.docx]

# S9 Fig


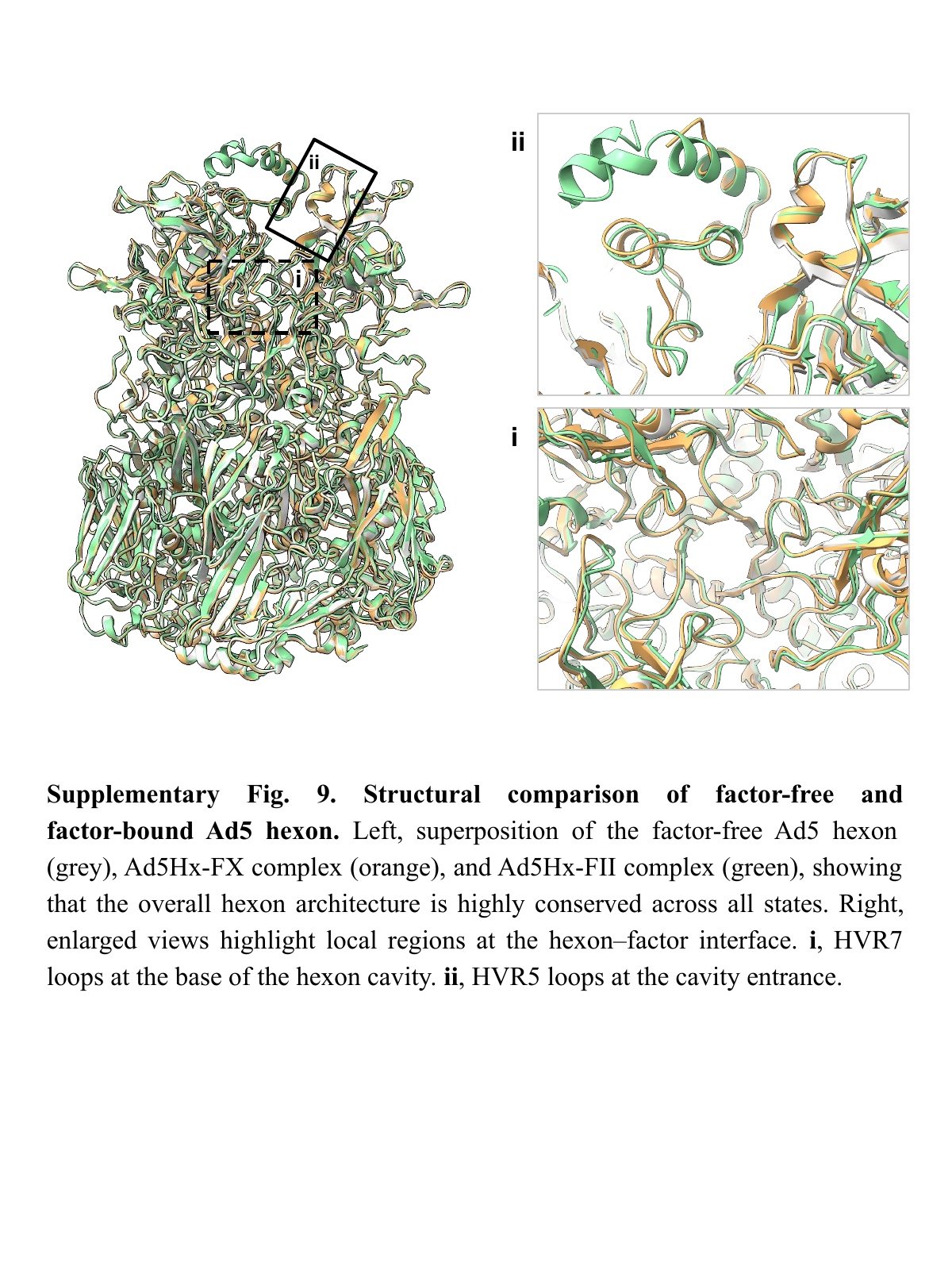

Supplement: S9 Fig — Left, superposition of the factor-free HAdV-C5 hexon (grey), Ad5Hx-FX complex (orange), and Ad5Hx-FII complex (green), showing that the overall hexon architecture is highly conserved across all states. Right, enlarged views highlight local regions at the hexon-factor interface. i, HVR7 loops at the base of the hexon cavity. ii, HVR5 loops at the cavity entrance. (DOCX) [file ppat.1014389.s009.docx]

# S10 Fig


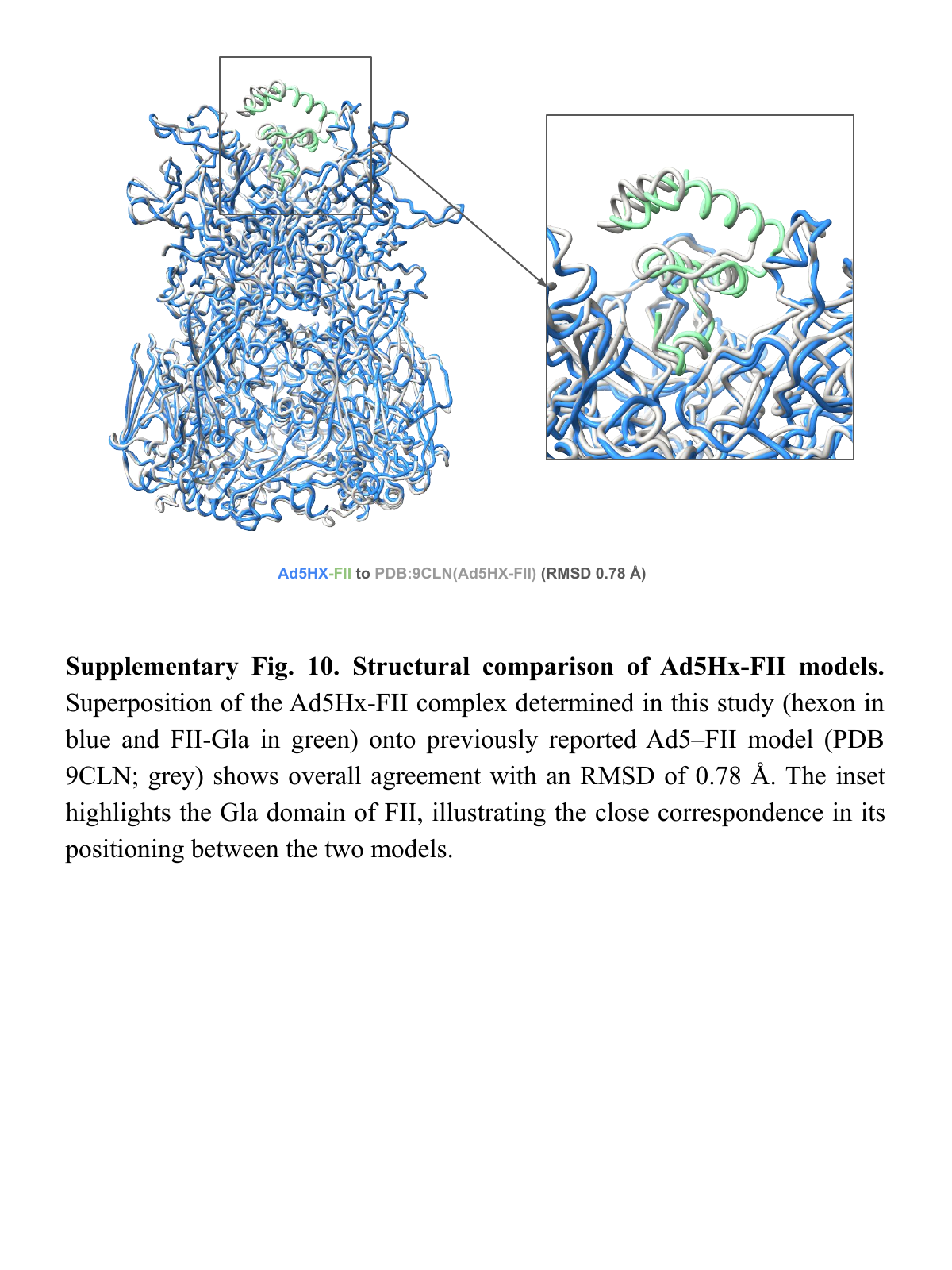

Supplement: S10 Fig — Superposition of the Ad5Hx-FII complex determined in this study (hexon in blue and FII-Gla in green) onto previously reported Ad5Hx-FII model (PDB 9CLN; grey) shows overall agreement with an RMSD of 0.78 Å. The inset highlights the Gla domain of FII, illustrating the close correspondence in its positioning between the two models. (DOCX) [file ppat.1014389.s010.docx]

# S11 Fig


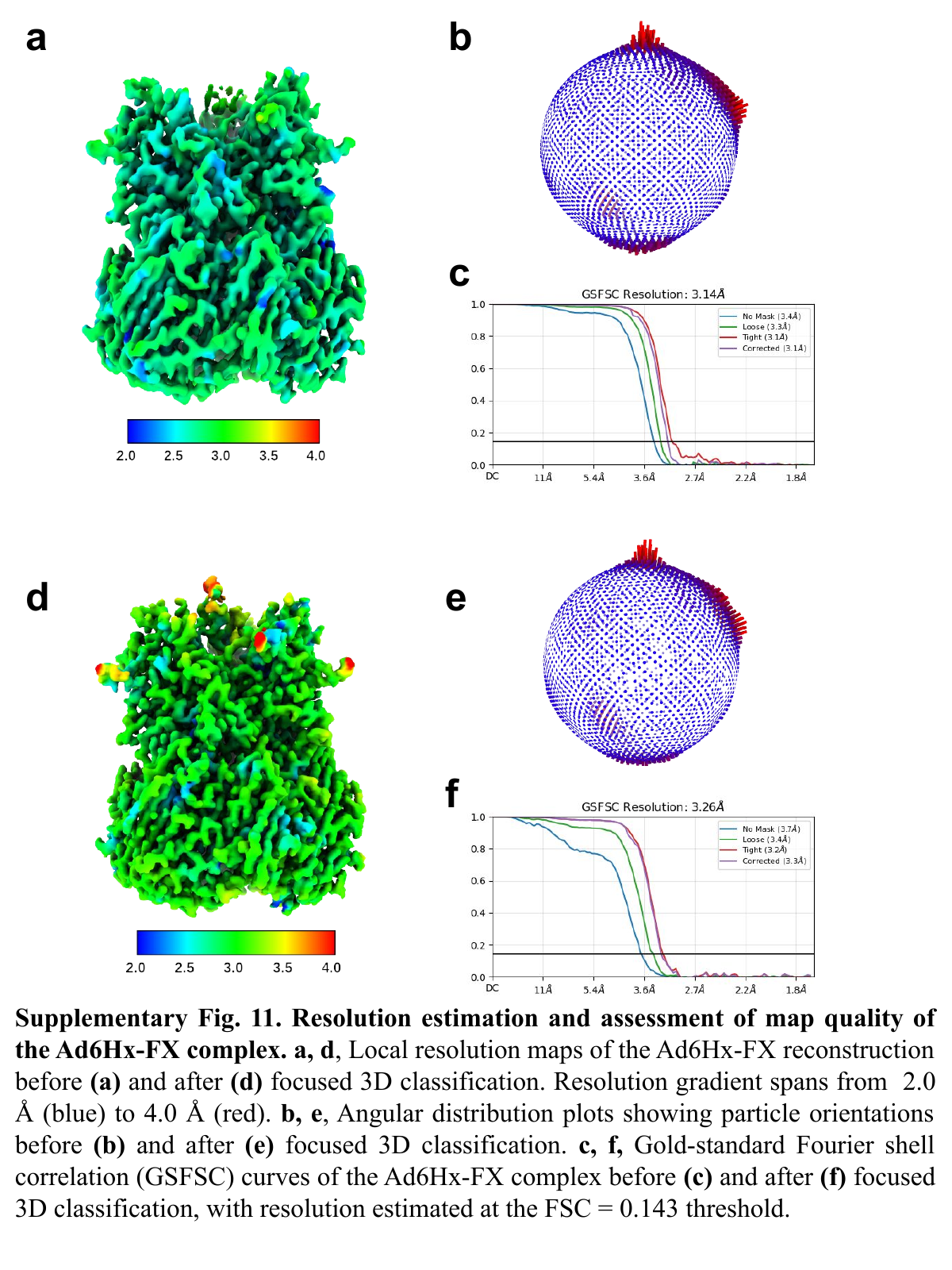

Supplement: S11 Fig — a, d, Local resolution maps of the Ad6Hx-FX reconstruction before (a) and after (d) focused 3D classification. Resolution gradient spans from 2.0 Å (blue) to 4.0 Å (red). b, e, Angular distribution plots showing particle orientations before (b) and after (e) focused 3D classification. c, f, Gold-standard Fourier shell correlation (GSFSC) curves of the Ad6Hx-FX complex before (c) and after (f) focused 3D classification, with resolution estimated at the FSC = 0.143 threshold. (DOCX) [file ppat.1014389.s011.docx]

# S12 Fig


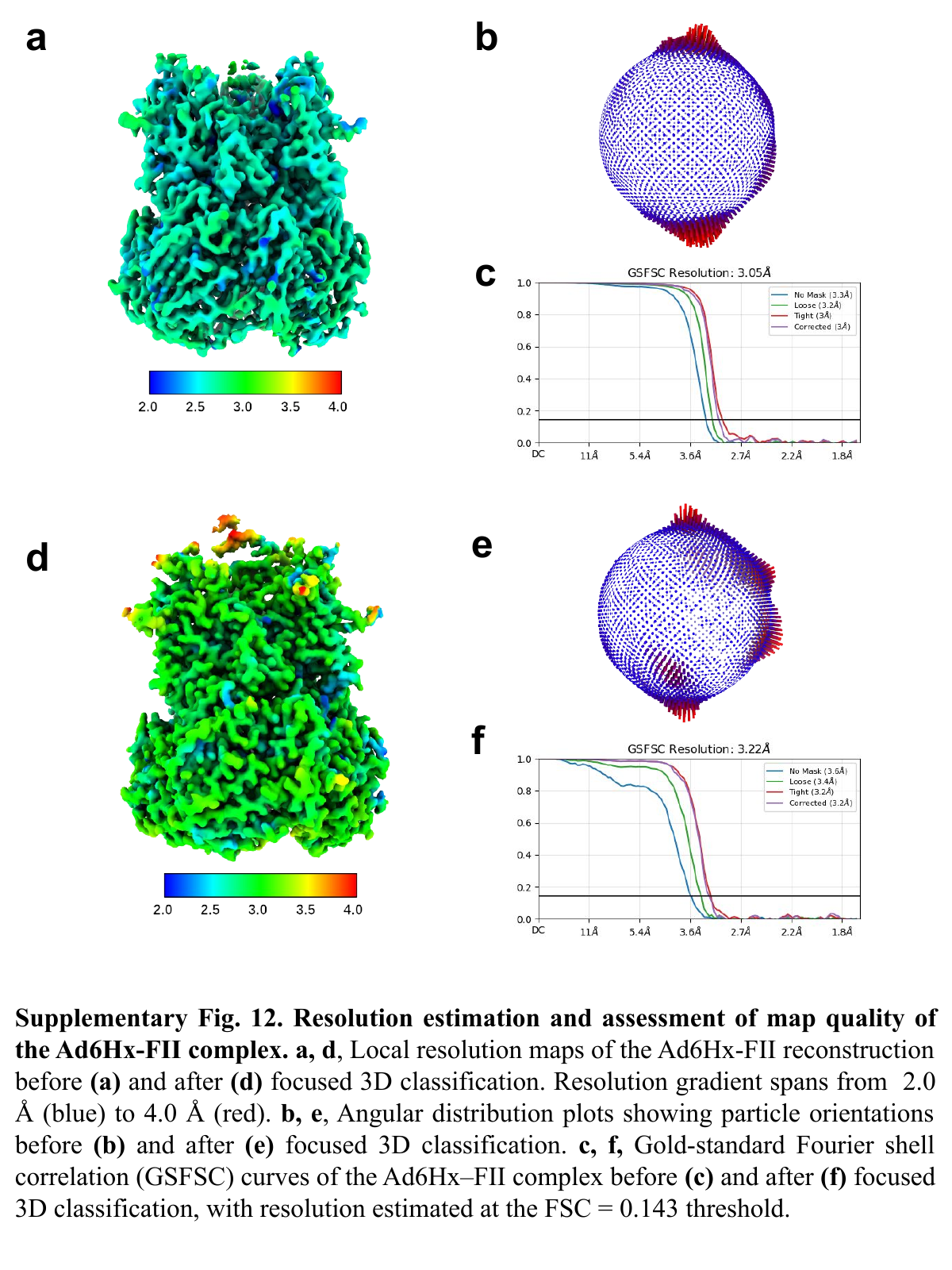

Supplement: S12 Fig — a, d, Local resolution maps of the Ad6Hx-FII reconstruction before (a) and after (d) focused 3D classification. Resolution gradient spans from 2.0 Å (blue) to 4.0 Å (red). b, e, Angular distribution plots showing particle orientations before (b) and after (e) focused 3D classification. c, f, Gold-standard Fourier shell correlation (GSFSC) curves of the Ad6Hx-FII complex before (c) and after (f) focused 3D classification, with resolution estimated at the FSC = 0.143 threshold. (DOCX) [file ppat.1014389.s012.docx]

# S13 Fig


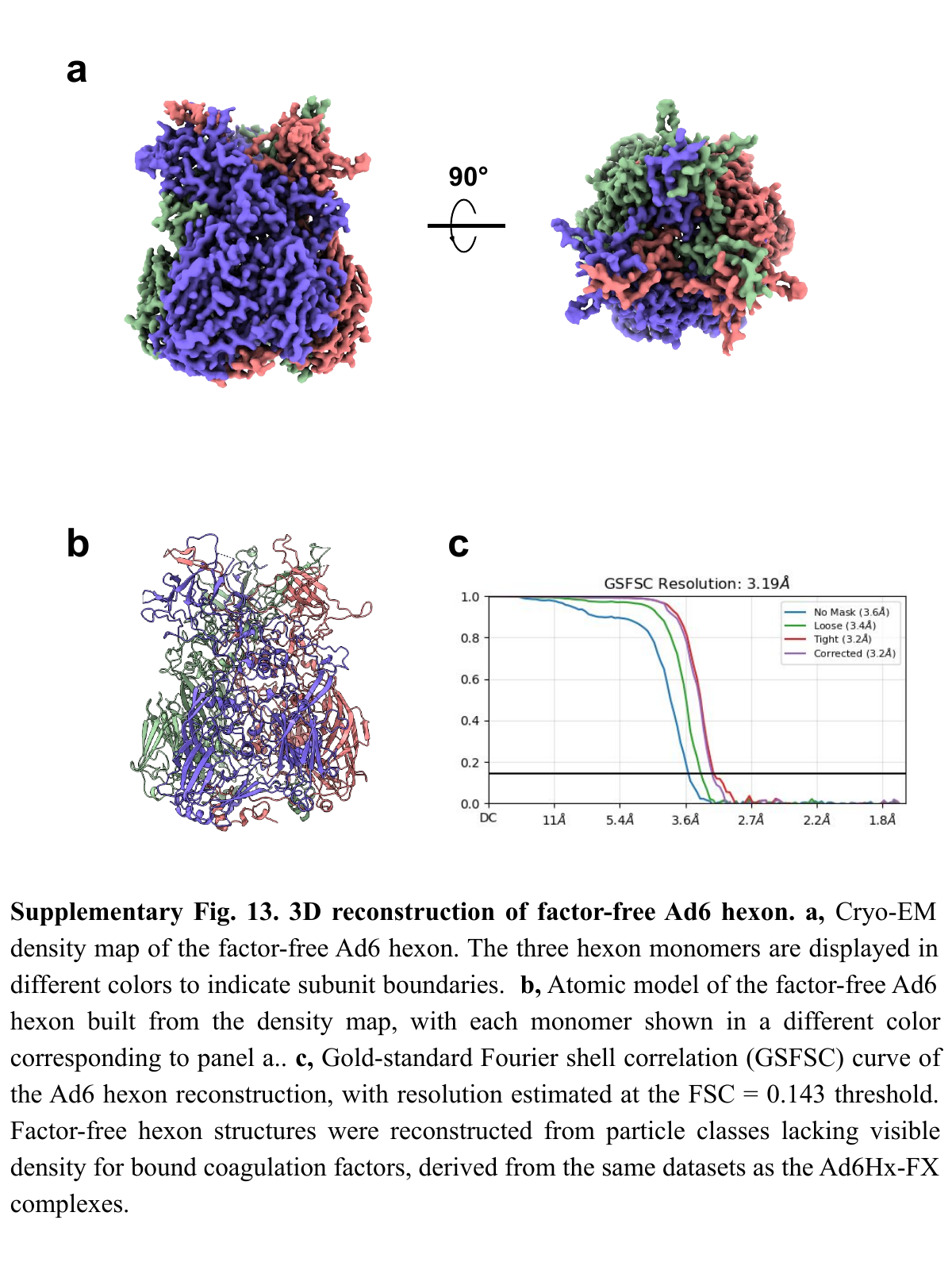

Supplement: S13 Fig — a, Cryo-EM density map of the factor-free HAdV-C6 hexon. The three hexon monomers are displayed in different colors to indicate subunit boundaries. b, Atomic model of the factor-free HAdV-C6 hexon built from the density map, with each monomer shown in a different color corresponding to panel a. c, Gold-standard Fourier shell correlation (GSFSC) curve of the HAdV-C6 hexon reconstruction, with resolution estimated at the FSC = 0.143 threshold. Factor-free hexon structures were reconstructed from particle classes lacking visible density for bound coagulation factors, derived from the same datasets as the Ad6Hx-FX complexes. (DOCX) [file ppat.1014389.s013.docx]

# S14 Fig


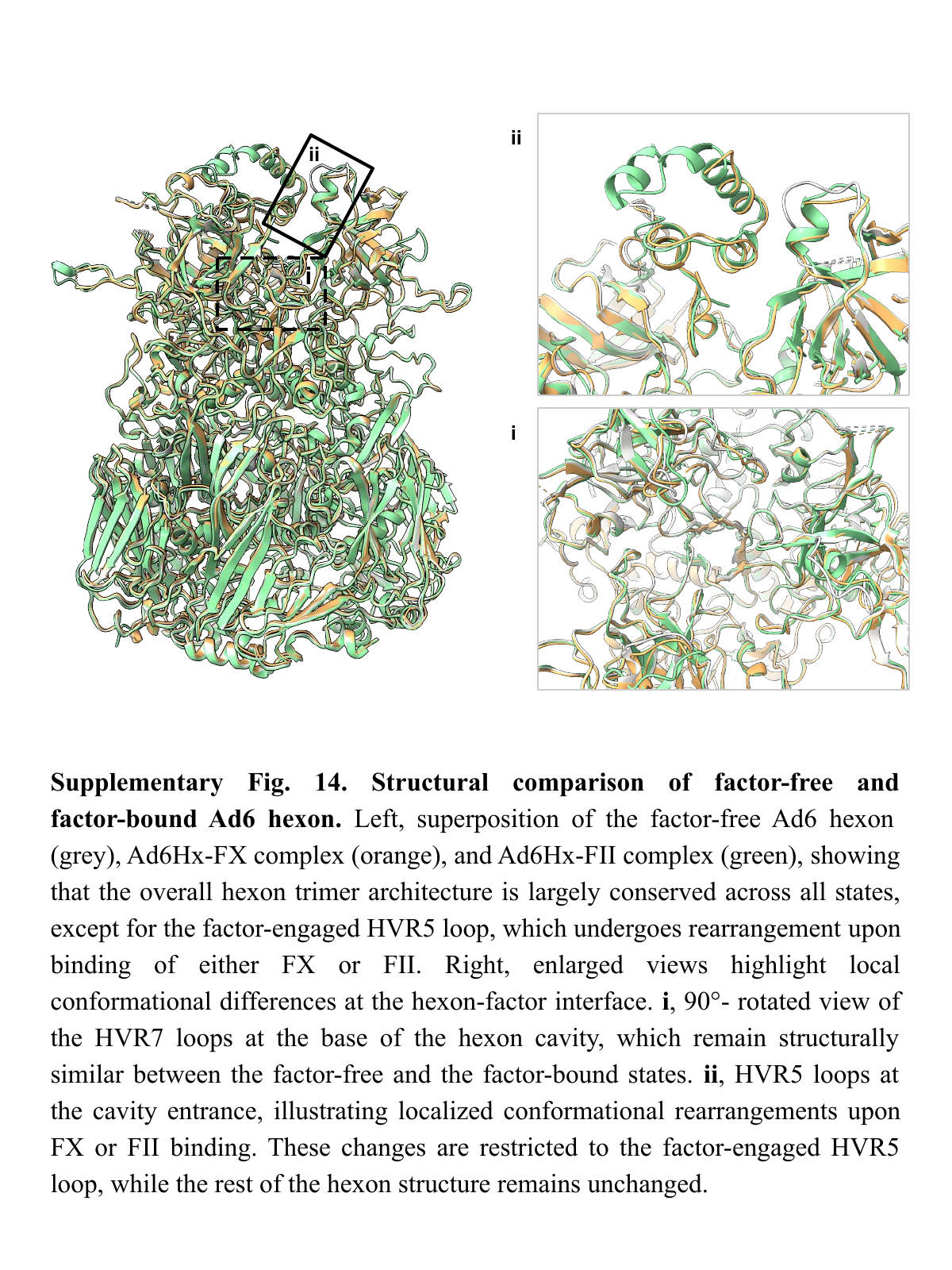

Supplement: S14 Fig — Left, superposition of the factor-free HAdV-C6 hexon (grey), Ad6Hx-FX complex (orange), and Ad6Hx-FII complex (green), showing that the overall hexon trimer architecture is largely conserved across all states, except for the factor-engaged HVR5 loop, which undergoes rearrangement upon binding of either FX or FII. Right, enlarged views highlight local conformational differences at the hexon-factor interface. i, 90°- rotated view of the HVR7 loops at the base of the hexon cavity, which remain structurally similar between the factor-free and the factor-bound states. ii, HVR5 loops at the cavity entrance, illustrating localized conformational rearrangements upon FX or FII binding. These changes are restricted to the factor-engaged HVR5 loop, while the rest of the hexon structure remains unchanged. (DOCX) [file ppat.1014389.s014.docx]

# S15 Fig


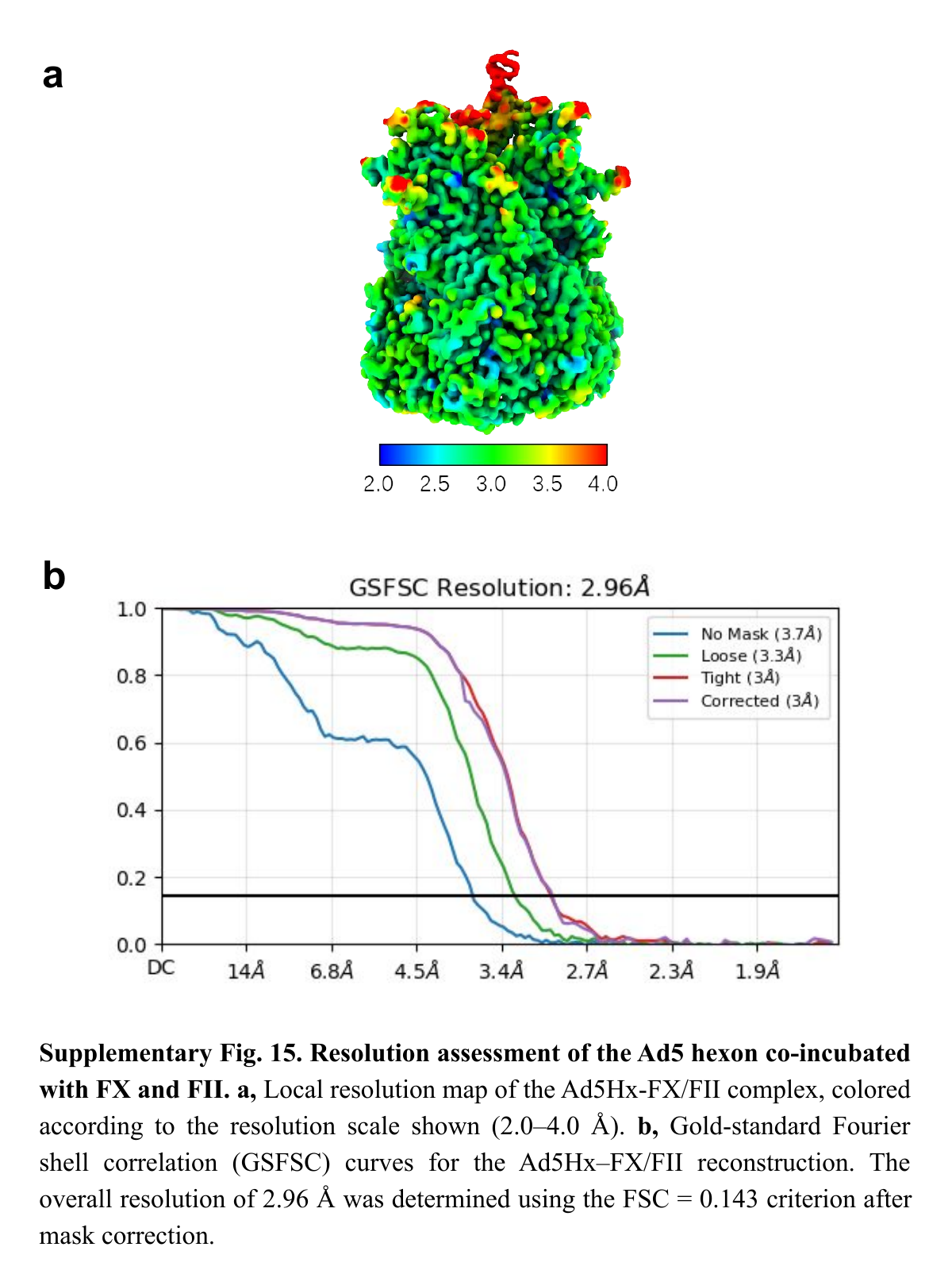

Supplement: S15 Fig — a, Local resolution map of the Ad5Hx-FX/FII complex, colored according to the resolution scale shown (2.0-4.0 Å). b, Gold-standard Fourier shell correlation (GSFSC) curves for the Ad5Hx-FX/FII reconstruction. The overall resolution of 2.96 Å was determined using the FSC = 0.143 criterion after mask correction. (DOCX) [file ppat.1014389.s015.docx]

# S16 Fig


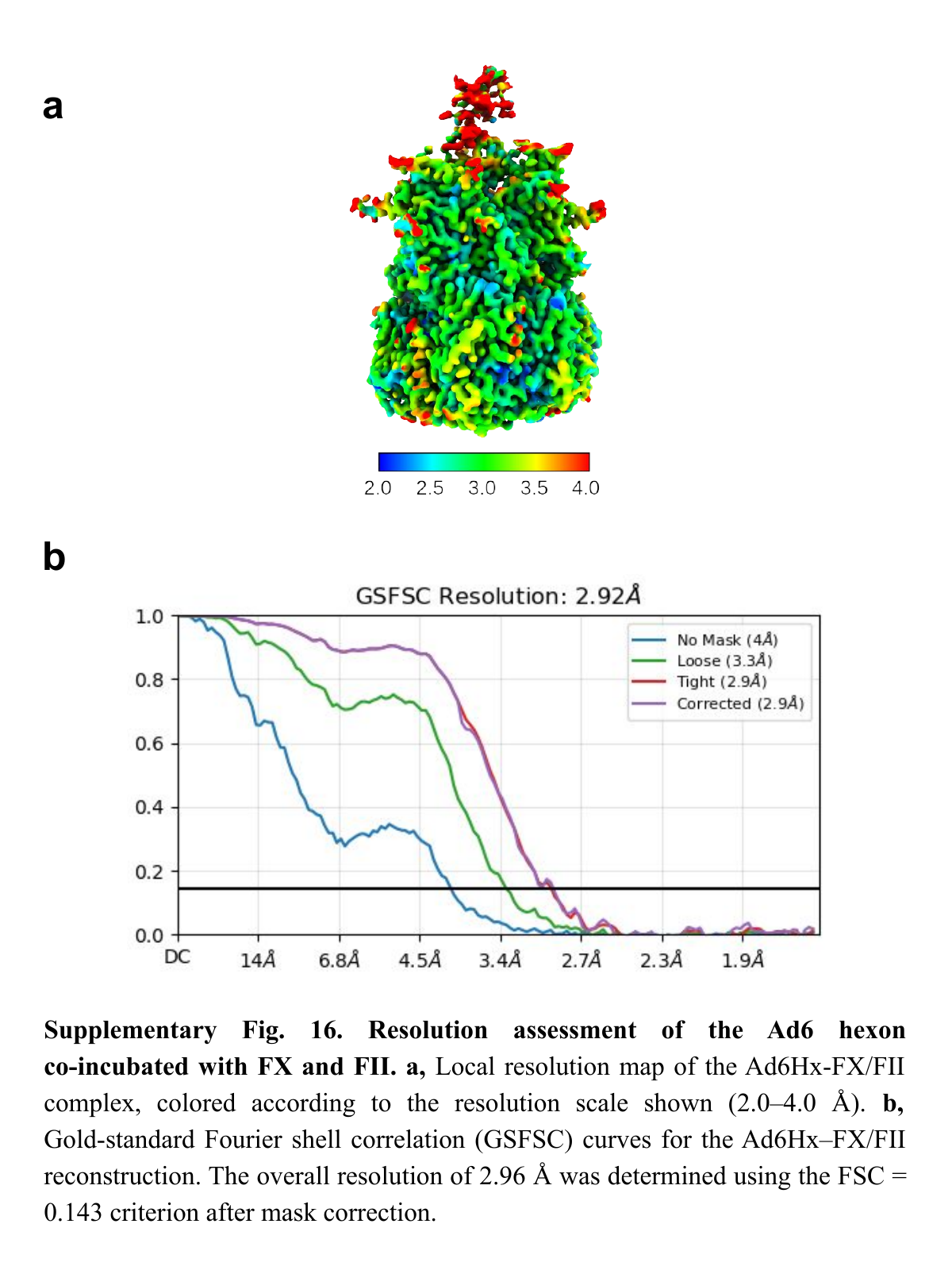

Supplement: S16 Fig — a, Local resolution map of the Ad6Hx-FX/FII complex, colored according to the resolution scale shown (2.0-4.0 Å). b, Gold-standard Fourier shell correlation (GSFSC) curves for the Ad6Hx-FX/FII reconstruction. The overall resolution of 2.96 Å was determined using the FSC = 0.143 criterion after mask correction. (DOCX) [file ppat.1014389.s016.docx]

# S17 Fig


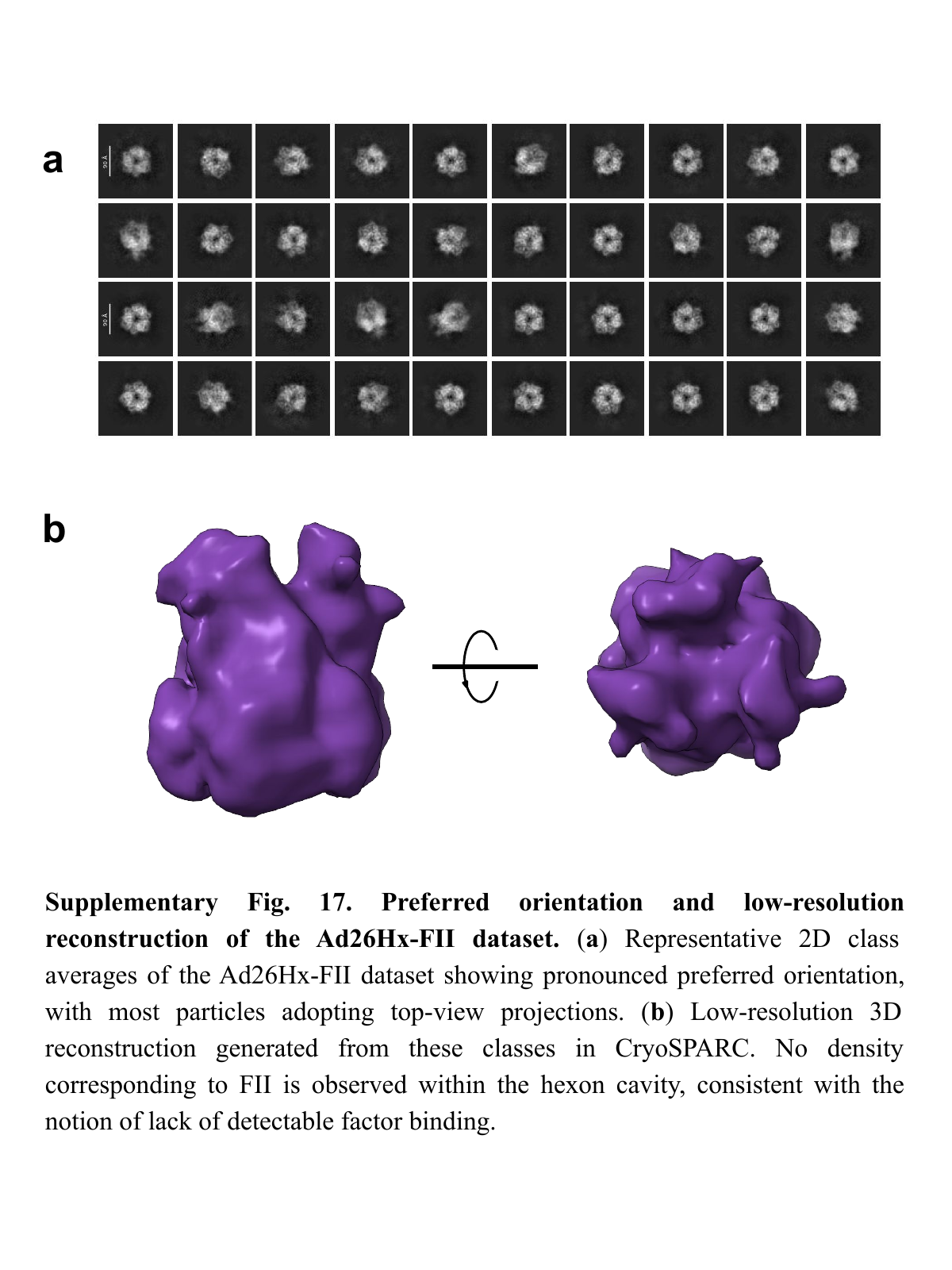

Supplement: S17 Fig — (a) Representative 2D class averages of the Ad26Hx-FII dataset showing pronounced preferred orientation, with most particles adopting top-view projections. (b) Low-resolution 3D reconstruction generated from these classes in CryoSPARC. No density corresponding to FII is observed within the hexon cavity, consistent with the notion of lack of detectable factor binding. (DOCX) [file ppat.1014389.s017.docx]

# S18 Fig


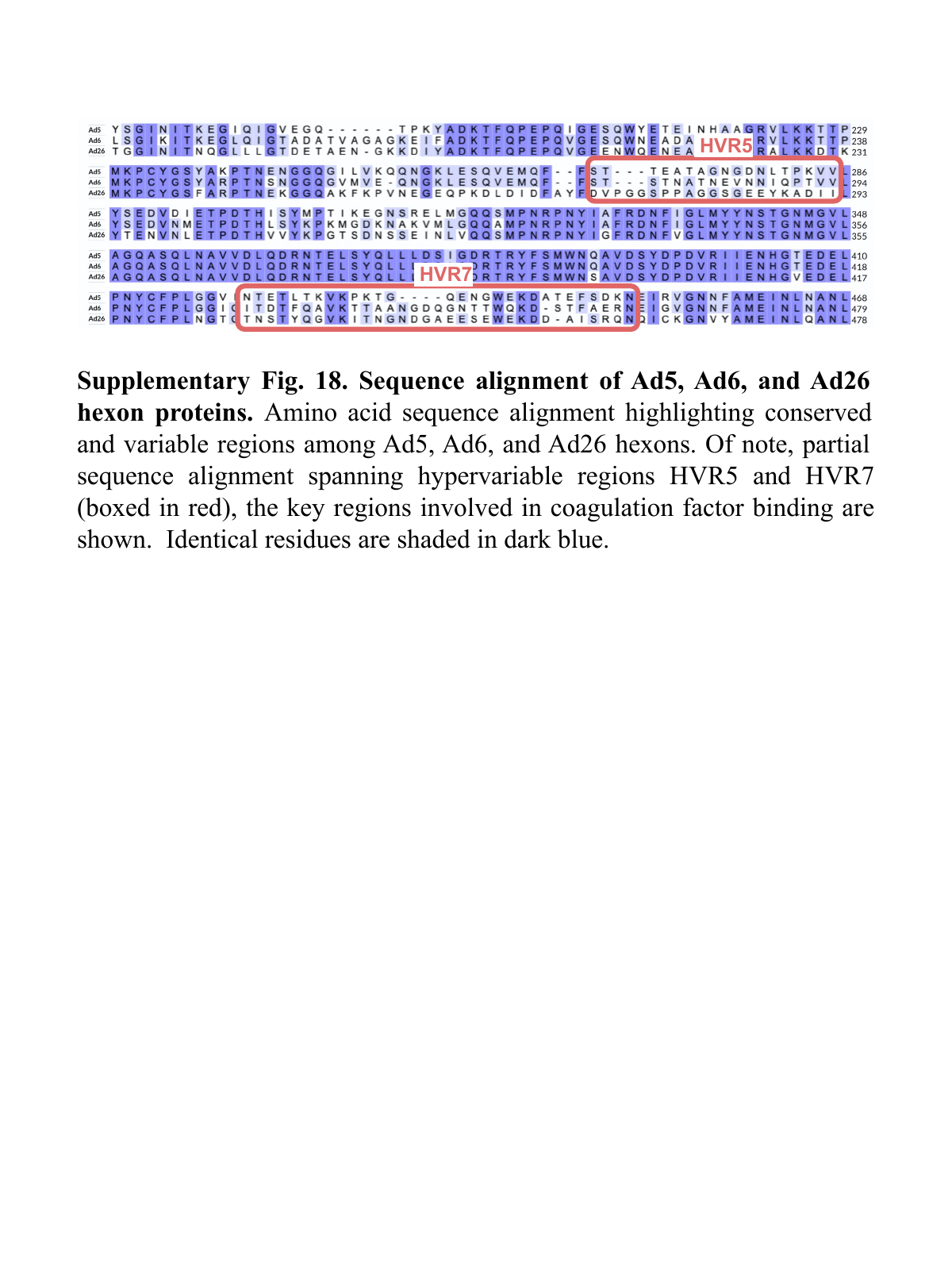

Supplement: S18 Fig — Amino acid sequence alignment highlighting conserved and variable regions among HAdV-C5, HAdV-C6, and HAdV-D26 hexons. Of note, partial sequence alignment spanning hypervariable regions HVR5 and HVR7 (boxed in red), the key regions involved in coagulation factor binding are shown. Identical residues are shaded in dark blue. (DOCX) [file ppat.1014389.s018.docx]

# S19 Fig


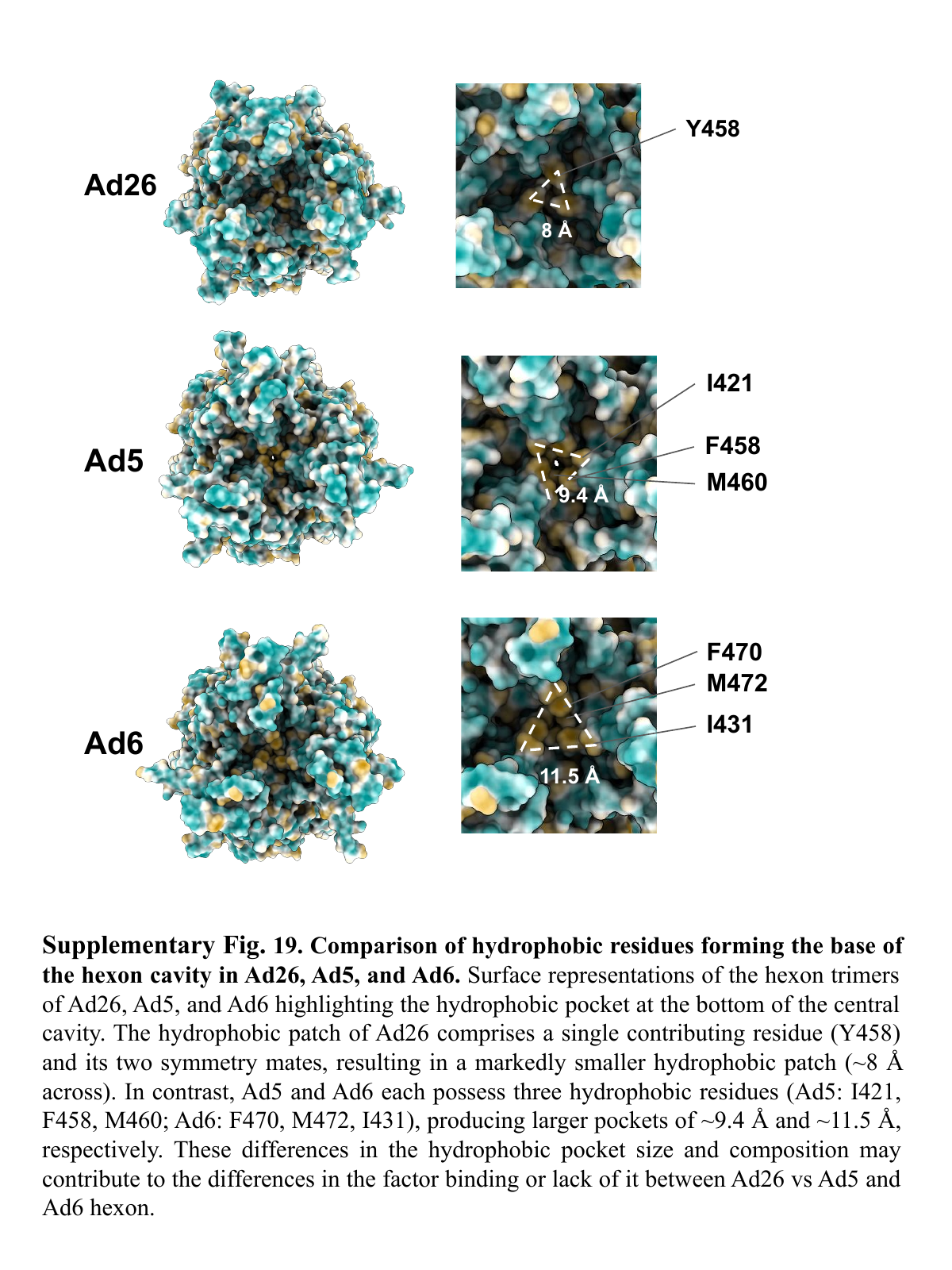

Supplement: S19 Fig — Surface representations of the hexon trimers of HAdV-D26, HAdV-C5, and HAdV-C6 highlighting the hydrophobic pocket at the bottom of the central cavity. The hydrophobic patch of Ad26Hx comprises a single contributing residue (Y458) and its two symmetry mates, resulting in a markedly smaller hydrophobic patch (~8 Å across). In contrast, Ad5Hx and Ad6Hx each possess three hydrophobic residues (Ad5: I421, F458, M460; Ad6: F470, M472, I431), producing larger pockets of ~9.4 Å and ~11.5 Å, respectively. These differences in the hydrophobic pocket size and composition may contribute to the differences in the factor binding or lack of it between Ad26Hx vs Ad5Hx and Ad6Hx. (DOCX) [file ppat.1014389.s019.docx]

# S20 Fig


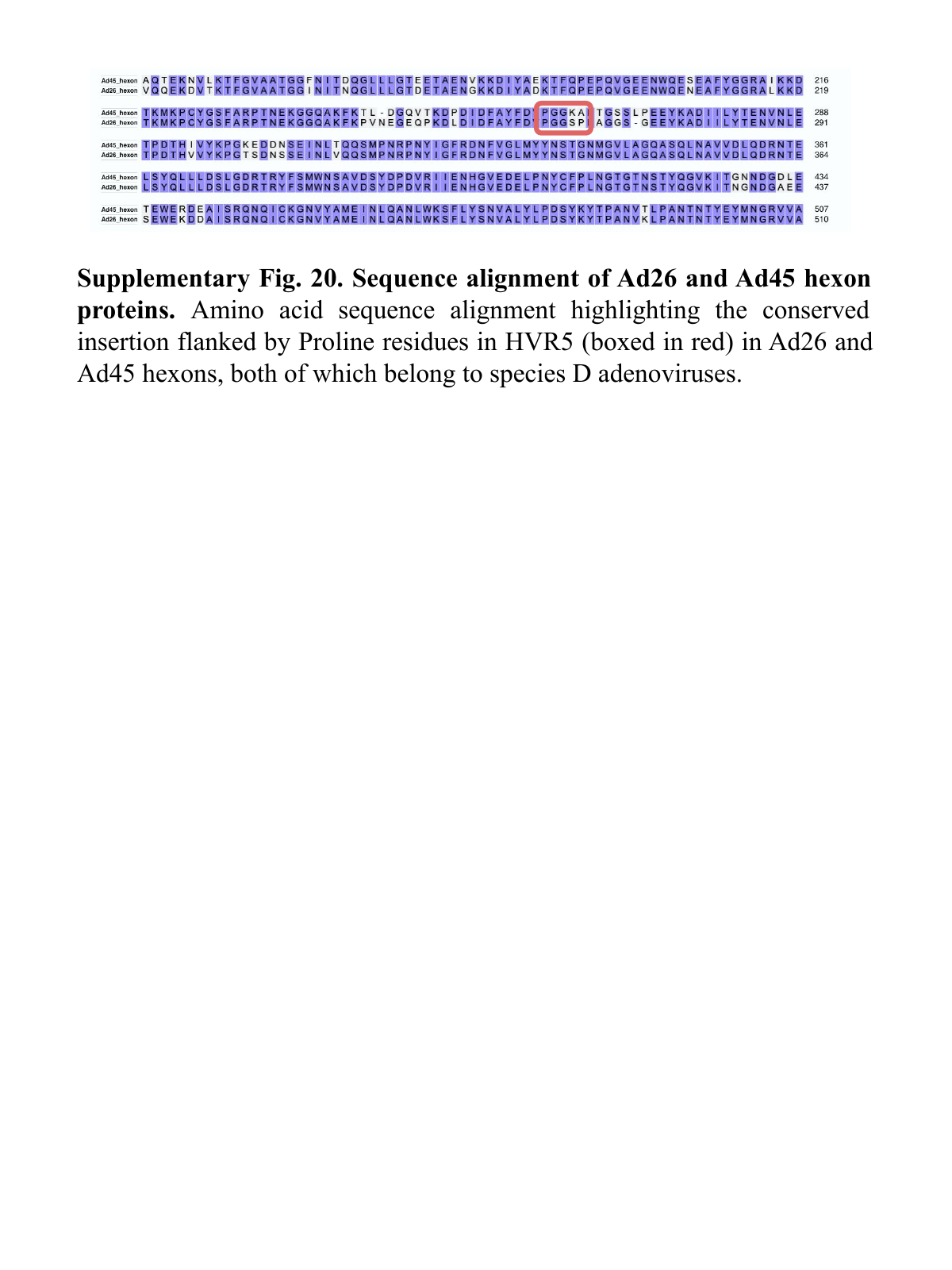

Supplement: S20 Fig — Amino acid sequence alignment highlighting the conserved insertion flanked by Proline residues in HVR5 (boxed in red) in HAdV-D26 and HAdV-D45 hexons, both of which belong to species D adenoviruses. (DOCX) [file ppat.1014389.s020.docx]

# S21 Fig


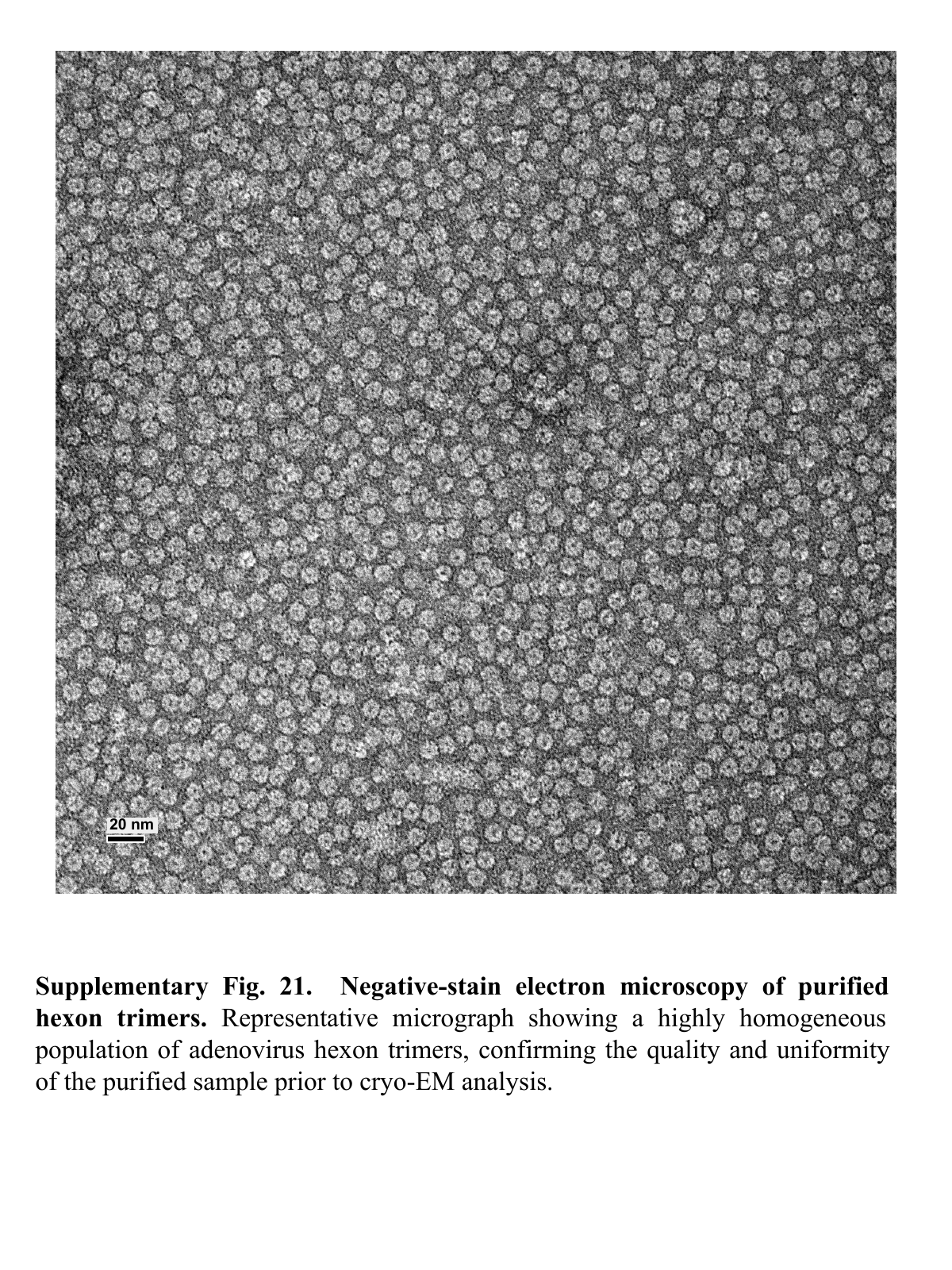

Supplement: S21 Fig — Representative micrograph showing a highly homogeneous population of adenovirus hexon trimers, confirming the quality and uniformity of the purified sample prior to cryo-EM analysis. (DOCX) [file ppat.1014389.s021.docx]

# S1 Table


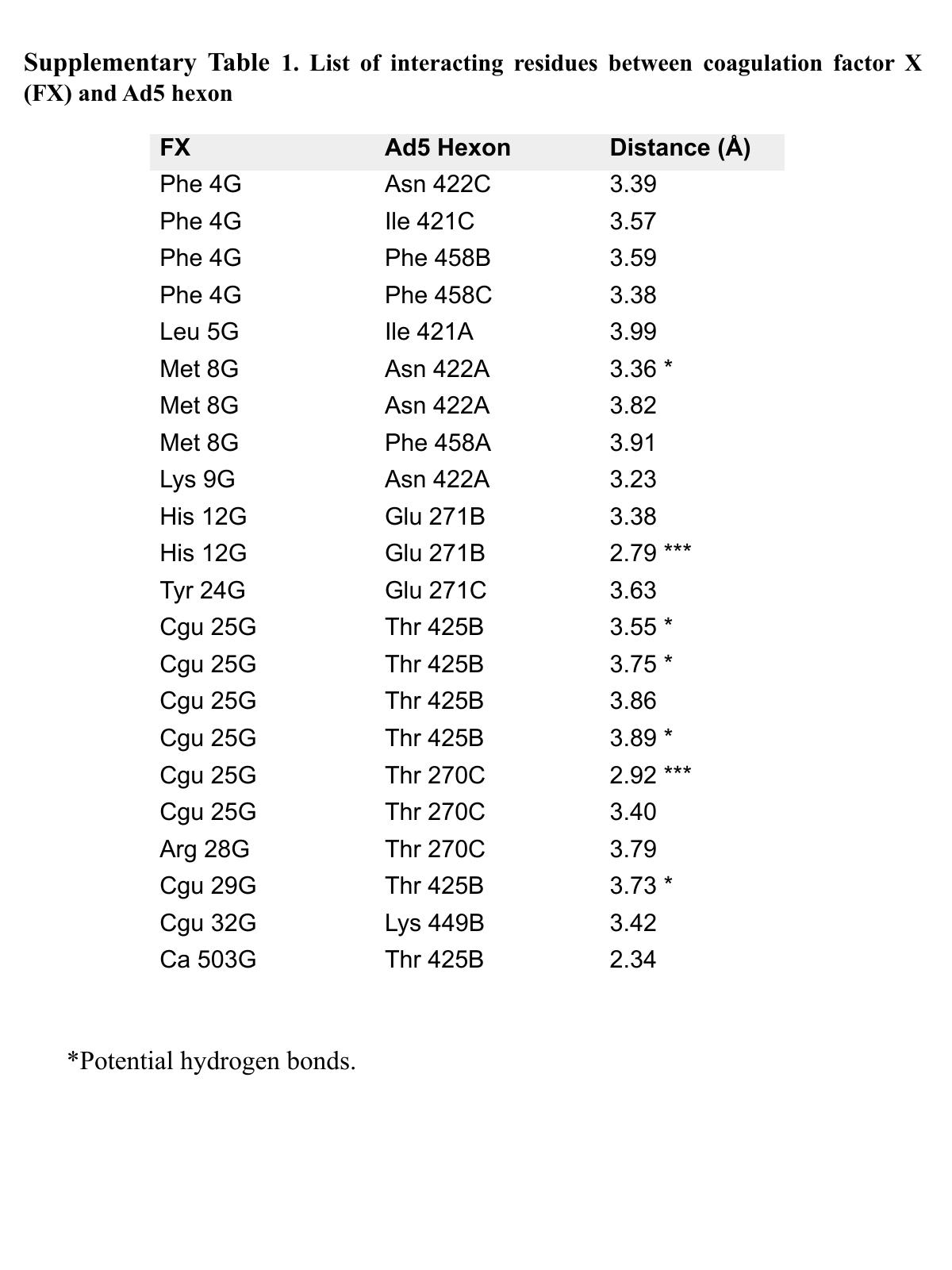

Supplement: S1 Table — (DOCX) [file ppat.1014389.s022.docx]

# S2 Table


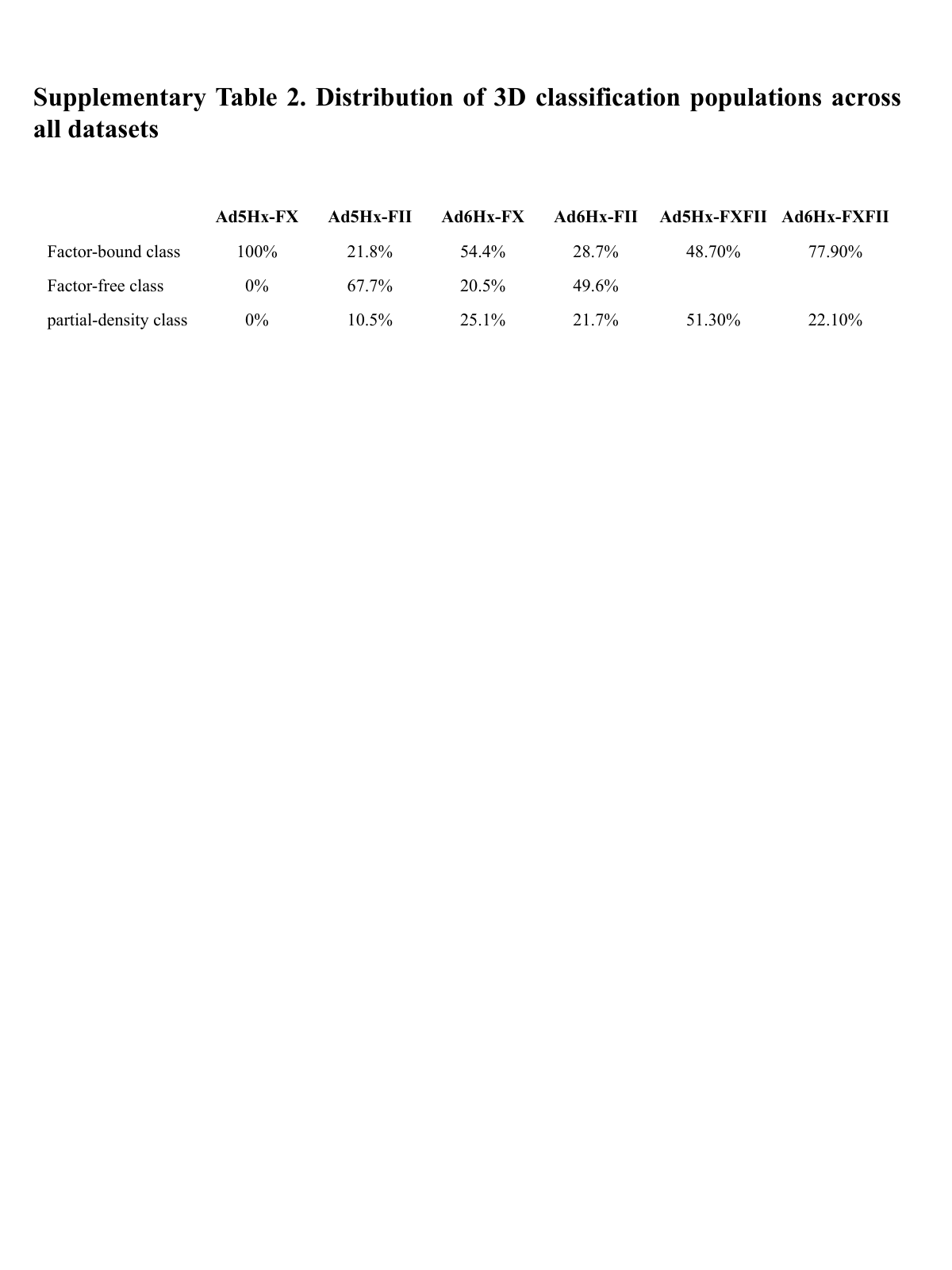

Supplement: S2 Table — (DOCX) [file ppat.1014389.s023.docx]

# S3 Table


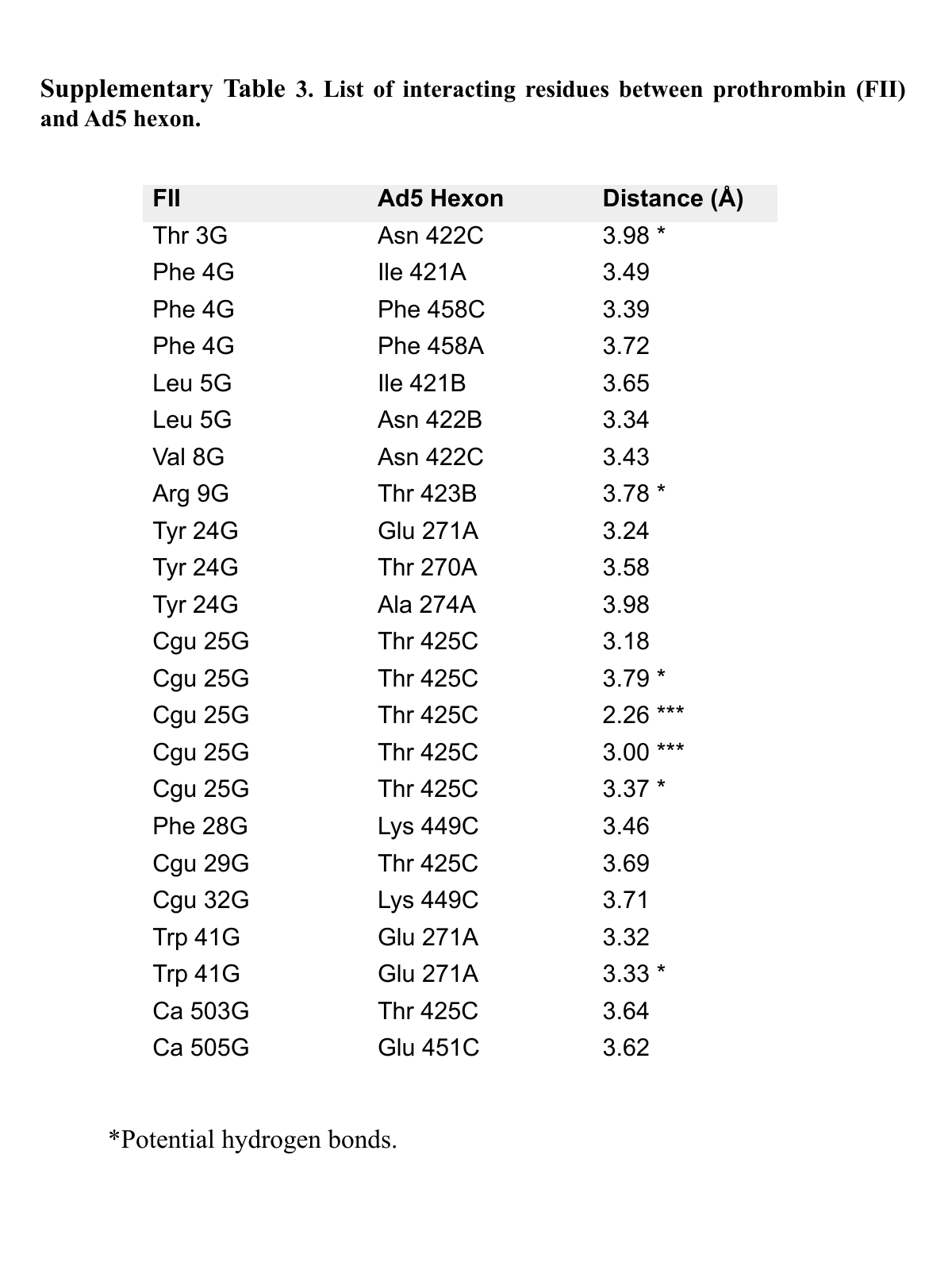

Supplement: S3 Table — (DOCX) [file ppat.1014389.s024.docx]

# S4 Table


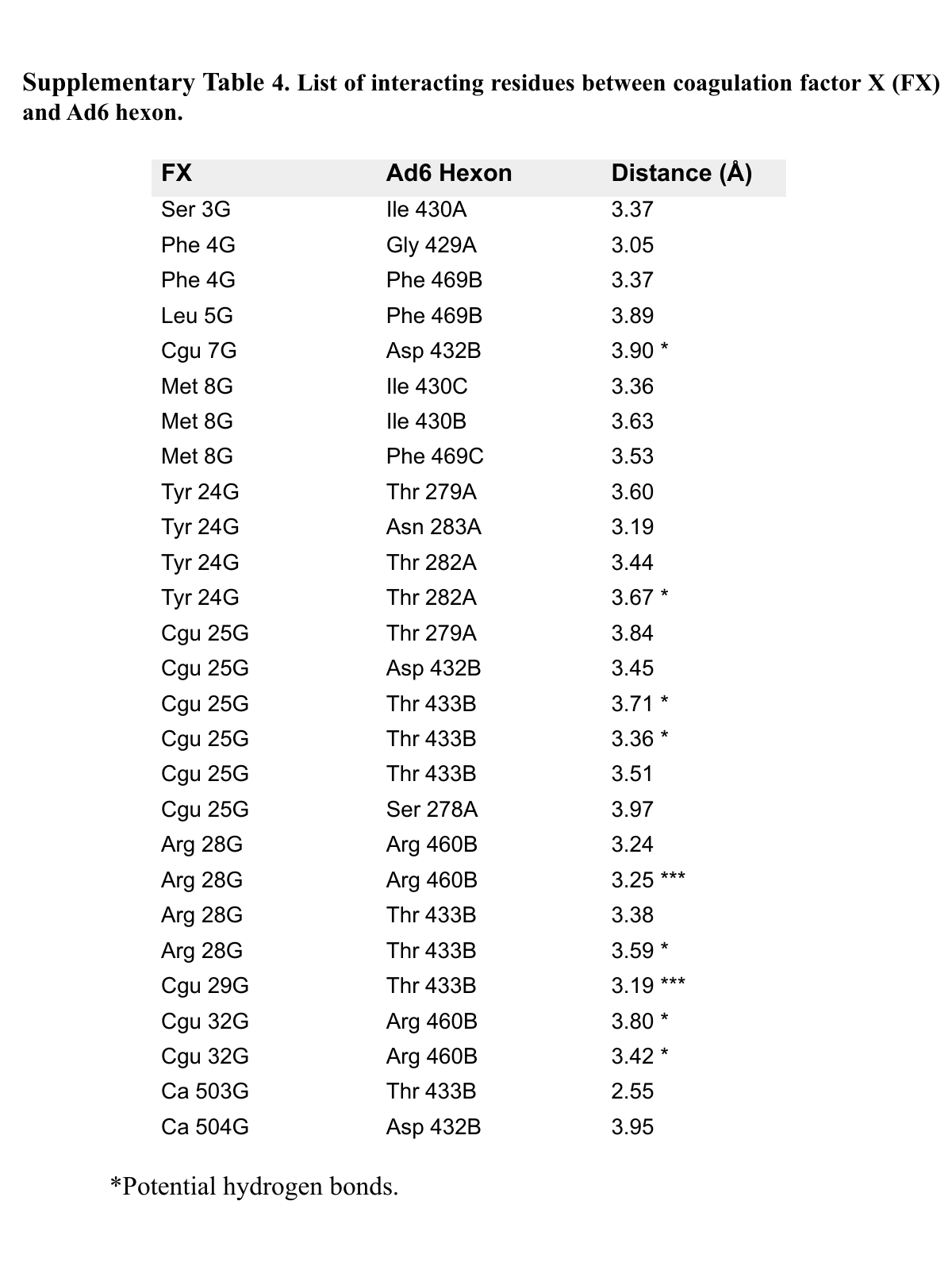

Supplement: S4 Table — (DOCX) [file ppat.1014389.s025.docx]

# S5 Table


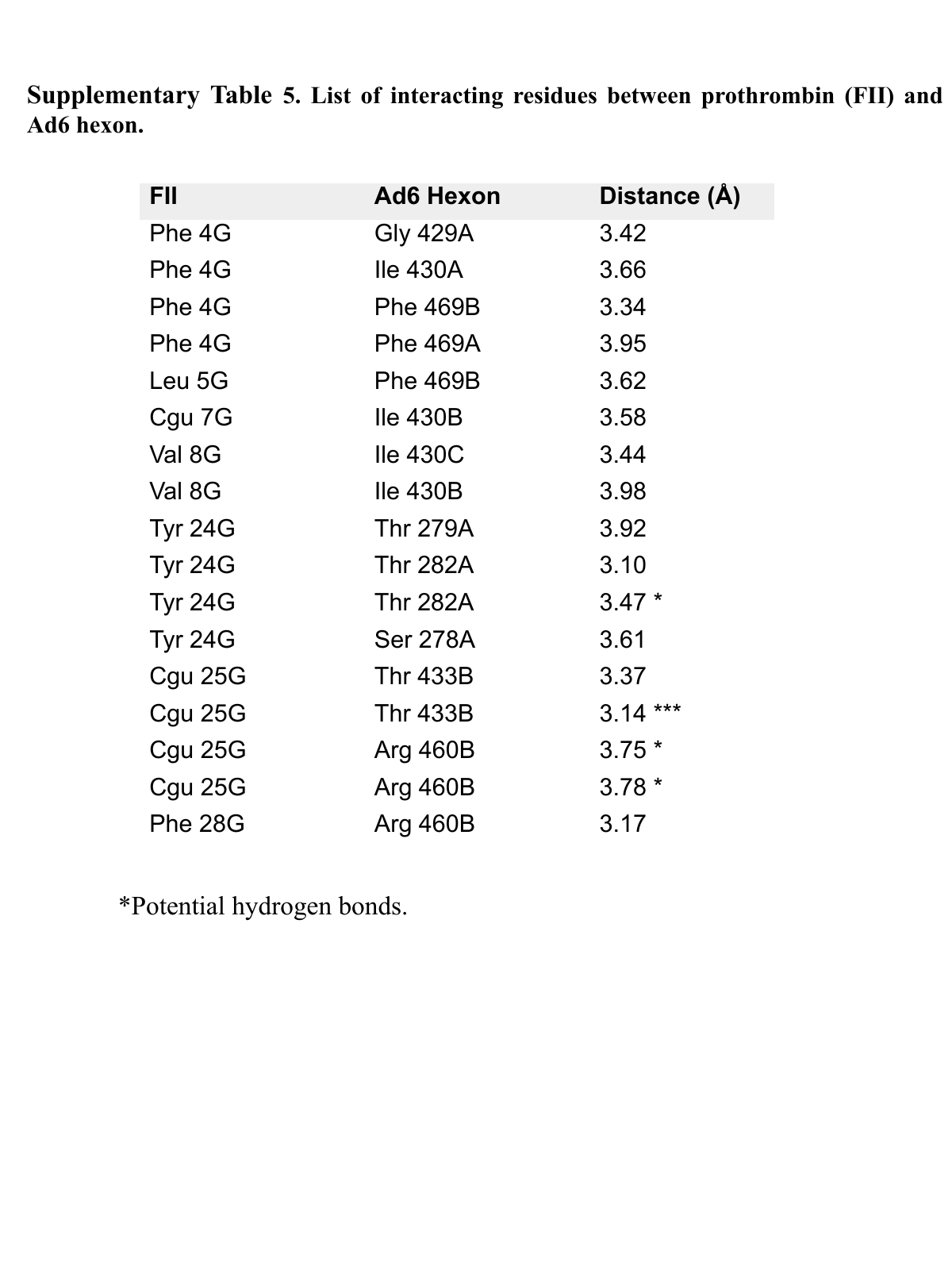

Supplement: S5 Table — (DOCX) [file ppat.1014389.s026.docx]

# S6 Table


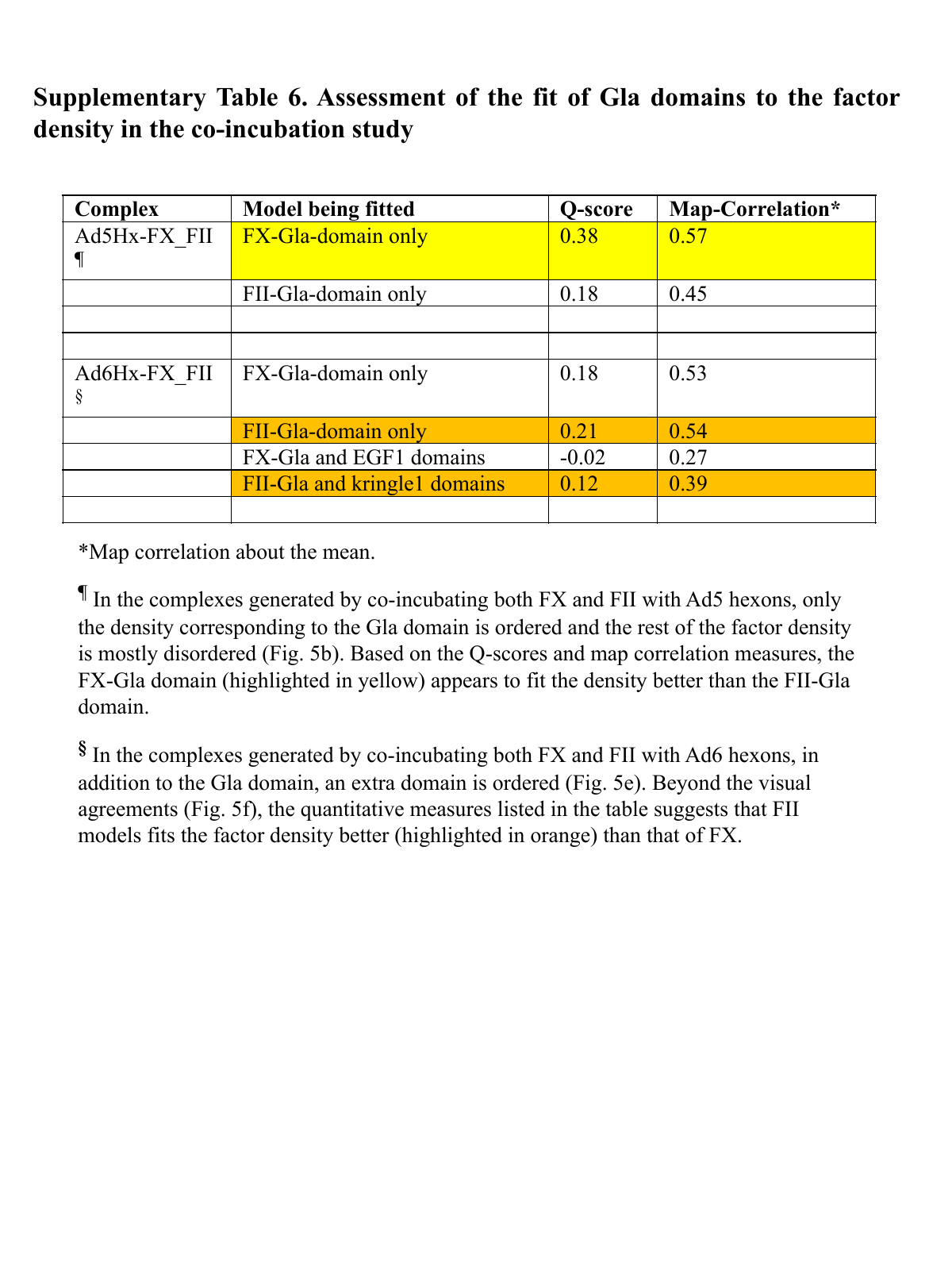

Supplement: S6 Table — (DOCX) [file ppat.1014389.s027.docx]
