# Supplementary material for: Structural requirements of blood factors binding to soluble hexon trimers with implications for adenovirus cell targeting and immune evasion
Source: PLoS Pathog. 2026 Jul 13;22(7):e1014389. doi: 10.1371/journal.ppat.1014389 (PMC13379079; doi:10.1371/journal.ppat.1014389)
Supplement: Attachment — Submitted filename: PlosPathogena_Response-to-reviews_VR_edtd-5_Final.pdf [file ppat.1014389.s028.pdf]

Responses to the reviewer's question

Part II – Major Issues: Key Experiments Required for Acceptance

Reviewer #1: (No Response)

Reviewer #2: none

Reviewer #3: 1. Given the similarity with the study by Mudrick et al 2024, I would suggest that the authors emphasize not only the differences in experimental strategy (as they do in lines 95-103), but also the new information provided by the present work.

We thank the reviewer for this helpful suggestion. We agree that more clearly distinguishing the novel insights of the present study in relation to Mudrick et al. (2024) strengthens the manuscript. In the revised manuscript (lines 83-96), we describe the main distinctions between the two studies.

Specifically, this study resolves the hexon-factor interactions at near-atomic resolution, allowing detailed identification of residue-level contacts within HVR5 and HVR7. Notably, the interacting residues in the Gla domain are clearly resolved in the current study compared to docking of the factor model into the corresponding density in the previous study (Mudrick et al. 2024). The current study also reveals distinct patterns of factor engagement by Ad5 and Ad6 hexons for FX and FII under co-incubation conditions. In addition, we provide a structural explanation for the reduced coagulation factor binding observed in Ad26, attributed to steric constraints within the HVR5 loop.

To address the reviewer's comments, we have revised the Introduction section to more explicitly articulate the above distinctions and clarify how they extend the knowledge and highlight the significance of factor binding to Ads (lines 83-96).

2. The most novel aspect is the study of co-incubation with both blood factors. However, it is difficult to evaluate the preference for one or the other with the information provided, in particular in case of Ad5: the fit to the density shown in Fig 5c is not so clear. The authors might want to quantify the fit of both factors, for example using the Q-score metric, or feeding the density to an algorithm such as ModelAngelo.

We thank the reviewer for this insightful suggestion and agree that quantitative evaluation of model-map agreement strengthens the interpretation of factor preference in the co-incubation experiments.

To address this point, we performed quantitative fitting analyses of the FX and FII models against the corresponding density maps using Q-score and map-model correlation metrics. For the Ad5 hexon co-incubation dataset, the FX Gla domain shows a substantially better fit to the density compared to the FII Gla domain (Q-score: 0.38 vs

0.18; map correlation: 0.57 vs 0.45), supporting preferential engagement of FX under these conditions. However, for the Ad6 dataset, the FII model shows modestly better agreement with the density compared to FX (Q-score: 0.21 vs 0.18; map correlation: 0.54 vs 0.53), favoring FII binding over FX. These results are summarized in a new supplementary table (Supplementary Table 6).

We have revised the Results section to incorporate these quantitative comparisons and to clarify that factor assignment is based on both visual inspection; Q-scores and model-map correlation metrics (lines 207-218). In addition, we have updated the figure legend for Fig. 5 to explicitly indicate the basis for model assignment (line 520, line 528).

3. It is also not clear if the authors maintained the blood factor:hexon ratio from the other experiments for either one of the factors in the co-incubation assays. That is: did they use 2 FX and 20 FII molecules per hexon trimer?

We thank the reviewer for this important point and agree that the stoichiometry used in the co-incubation experiments should be stated more clearly.

In the experiments involving single factors, hexon trimers were incubated with each coagulation factor at a 1:2 (hexon trimer: factor) molar ratio. In the co-incubation experiments, we maintained the same relative scaling while introducing both factors simultaneously at a physiologically relevant ratio. Specifically, hexon trimers were incubated with FX and FII at a molar ratio of 1:2:20 (hexon: FX: FII), corresponding to a 1:10 ratio of FX to FII. Thus, the amount of FX per hexon trimer was kept consistent with the single-factor experiments, while FII was added in excess to reflect its higher physiological abundance.

We have revised the Results and Methods sections to explicitly state these ratios and to clarify the design of the co-incubation experiments (lines 200-204 and lines 364-365).

4. In the same line, I am not sure if the authors can conclude that Ad6 binds FII preferentially (lines 241–253), since in this experiment FII was present in a concentration 10-fold higher than FX. And this does not correlate with the amount of factor-bound hexons in the single incubation experiments, where there seem to be more Ad6 hexons with FX density (54%, fig 3a) than with FII (29%, Fig 4a). Finally, in lines 332–333 it is stated that the conformational change of HVR5 “enables Ad6 hexon to accommodate both factors efficiently”, which seems a contradiction with line 219.

We thank the reviewer for this careful observation and for highlighting the need to clarify the interpretation of factor binding in Ad6.

We agree that the co-incubation experiments, where FII is present at a tenfold higher concentration than FX, do not allow us to directly infer intrinsic binding preference or affinity. Instead, these experiments reflect factor engagement under competitive

conditions where ligand availability differs. We have therefore revised the text to avoid overstating preferential binding and now describe these results as indicating that Ad6 hexon appear to engage FII more frequently under the tested conditions.

We also agree that the apparent discrepancy between the co-incubation and single-factor experiments needs clarification. In the single-factor a higher fraction of particles displays FX density compared to FII, which may reflect differences in binding efficiency or occupancy under non-competitive conditions. In contrast, in the co-incubation experiment, the relative excess of FII likely shifts the observed population toward FII-bound states. We have revised the manuscript to explicitly state this distinction.

Finally, we have clarified the description of the conformational change in HVR5. Rather than implying equivalent efficiency in binding both factors, we now state that this structural rearrangement enables accommodation of coagulation factors in general, without implying equal binding behavior.

These revisions have been incorporated into the Results and Discussion sections (lines 218-223, lines 298-300).

5. The authors should provide EMDB and PDB IDs and validation reports before final acceptance.

We thank the reviewer for this comment. The cryo-EM maps and corresponding atomic models have now been deposited in the Electron Microscopy Data Bank (EMDB) and the Protein Data Bank (PDB). The accession codes have been included in the revised manuscript and will be made publicly available upon publication.

Part III – Minor Issues: Editorial and Data Presentation Modifications
Please use this section for editorial suggestions as well as relatively minor modifications of existing data that would enhance clarity.

Reviewer #1: There are some minor concerns that would improve readability:

We thank the reviewer for the helpful suggestions.

1. Refer to the viruses as HAdV-C5, HAdV-C6, and HAdV-D26 throughout.

We have revised the manuscript to consistently use the nomenclature HAdV-C5, HAdV-C6, and HAdV-D26 throughout the text, figures, and figure legends.

2. Define “Gla domain” in the abstract.

We have revised the abstract to define the Gla domain (γ-carboxyglutamic acid domain) at its first occurrence for clarity (line 39).

3. There are more recent references than 4-6 to support the prominence of HAdV-based vectors in gene therapy and as vaccine platforms.

We have updated the Introduction to include more recent references highlighting the continued prominence of HAdV-based vectors in gene therapy and vaccine applications.

4. Line 67 – references here could be more inclusive of other studies cited elsewhere in the MS.

We have included additional relevant references, ensuring a more comprehensive representation of prior studies on adenovirus-coagulation factor interactions.

5. Lines 151–163 – the residue numbers mentioned here don’t appear to match the numbers in Fig 1.

We have re-checked the residue numbering and identified a few discrepancies between the text and Fig. 1. The residue numbers have now been corrected.

6. Fig 1ci – it would be helpful to distinguish the monomers, perhaps by different colors.

We have used a consistent color scheme in generating the figures to distinguish between different components and for the ease of following, with Ad5 hexon shown in blue, Ad6 hexon in purple, factor X in orange, and factor II in green.

We chose to maintain this unified color coding across all figures to facilitate comparison between complexes and to avoid confusion when interpreting multiple datasets. We agree that distinguishing individual monomers could further improve clarity, and we have therefore modified supplementary figures 8 and 13 to indicate monomers with different colors.

7. It’s a bit of an overstatement to call a single 1:10 mixture of FX and FII in PBS “physiologic” in mimicking how these proteins might bind to HAdVs in serum, which is a much more complex mixture.

We agree with reviewer's comment that the term "physiological conditions" as we have implied is an oversimplification of the complexity of serum composition.

The purpose of the coincubation study was to reflect the relative abundance of FX and FII, which differ approximately by an order of magnitude in the bloodstream, rather than to fully recapitulate the biochemical environment of serum. We have revised the text throughout the manuscript to clarify this point and now describe these conditions reflect relative abundance of FX and FII, rather than to emulate full complexity of physiological conditions.

Reviewer #2: I only have minor suggestions to improve this very nice contribution as noted below.

1. Adding Figure numbers to the attached figures would be helpful.

We sincerely apologize for this oversight. We now included the figure numbers on all main and supplementary figures.

2. Figure 2, difficult to visualize all 6 calcium ions bound to the hexon complexes containing each clotting factor. The authors should include a statement about this in the figure legend to clarify.

We thank the reviewer for this helpful observation. We agree that not all Ca^{2+} ions are clearly visualized in the density maps. We have revised the figure legend for Fig. 2 to clarify that while Ca^{2+} ions are included in the model based on expected coordination within the Gla domain and prior structural knowledge (PDB-ID: 1IOD), only a subset are supported by discernible density in our maps (lines 471-473).

3. Figure 5. Does the preference of FX binding to Ad5 hexons and FII binding to Ad6 hexons hold true for intact virions?

We thank the reviewer for this important question.

Distinguishing between FX and FII binding on intact virions under co-incubation conditions would be technically challenging, as the resulting density would likely be limited by the heterogeneity due to particle averaging. Additionally, the resulting lower resolution density at the factor binding regions would significantly hamper to reliably differentiate between the two factors, particularly given that only the Gla domain is well resolved in our datasets. In fact, that was one of the reasons for undertaking the study involving the soluble hexons. However, since the hexons are factor binding component in the context of Ad virions also, we surmise that similar preference will be observed with regards to the full virions.

We included this discussion in the manuscript (lines 83-85, lines 291-293).

4. The authors should substantiate the preference for binding of clotting factors by performing BIAcore or EliSA assays.

We thank the reviewer for this constructive suggestion. We agree that orthogonal biochemical approaches such as BIAcore or ELISA could provide additional information about factor binding in a complementary format. However, since the primary aim of the current study is to define the structural basis of coagulation factor engagement by the purified hexon trimers using cryo-EM, the suggested experiments are beyond the scope of the current study.

To address the reviewer's concern, we have revised the manuscript to avoid overinterpreting the co-incubation data as direct evidence of intrinsic binding preference or affinity. Instead, we now describe these results more cautiously as differential factor engagement under the specific competitive conditions tested (lines 219-223). In addition, we have strengthened the support for factor assignment in the co-incubation experiments by including quantitative model-map agreement analyses using Q-score and map-correlation metrics (lines 209-218). These analyses support the interpretation of the densities observed in Fig. 5.

We also note that previous studies (Tian et al., 2022), including our prior work, have already demonstrated coagulation factor association with adenovirus particles and identified hexon determinants involved in factor binding. We therefore believe that the revised version of the manuscript addresses this point within the scope of current study.

5. Discussion (lines 287–300). This part deserves more description in terms of how clotting factors alter binding of viruses to host cells.

We thank the reviewer for this helpful suggestion. We have revised the Discussion to more clearly describe how coagulation factors, particularly FX, mediate interactions between adenovirus particles and host cell surface molecules such as heparan sulfate proteoglycans, thereby promoting liver tropism and influencing viral uptake. We also discuss how factor binding can shield viral epitopes from immune recognition and how differences in factor engagement between serotypes may contribute to variations in biodistribution and immune responses.

These revisions have been incorporated into the Discussion sections (Lines 257-262).

Reviewer #3: Lines 53–55: a reference to the ICTV adenovirus taxonomy page would be appropriate.

We have added a reference to the ICTV adenovirus taxonomy resource in the Introduction (line 53).

Lines 392–397 and 338: please indicate the composition of the DPBS buffer as well as the concentration of added Ca and Mg cations.

We have revised the manuscript to explicitly indicate the composition of the DPBS buffer used in our experiments, including the concentrations of Ca^{2+} and Mg^{2+} ions. Specifically, we now state that DPBS contained [insert exact values, e.g., 137 mM NaCl, 2.7 mM KCl, 10 mM phosphate buffer (pH 7.4), 0.9 mM $CaCl_2$, and 0.5 mM $MgCl_2$]. These details have been added to the Methods section (lines 361-362).

Line 68: Is ref. 20 correct here?

We thank the reviewer for pointing this out. We have replaced it with a more appropriate reference.

Lines 311–315: "Ca ions are consistently observed…" This sentence sounds a bit misleading. Do the authors mean that they observe density corresponding to these cations? This does not seem to be the case from the Results section. Please clarify.

We thank the reviewer for pointing this out. We agree that the original wording could be interpreted as implying direct and unambiguous visualization of Ca^{2+} ions in all cases. Our intention was to indicate that the observed interactions are compatible with previously described Ca^{2+}-mediated coordination in coagulation factor Gla domains, rather than to claim that Ca^{2+} ions are directly resolved in our structures.

We have revised the sentence to clarify that density features consistent with Ca^{2+} coordination is observed in the Gla domain region, and that the placement of Ca^{2+} ions is based on prior structural knowledge and modeling, rather than uniformly resolved density in all datasets (lines 282-284).

Lines 317–318: 80% of Ad5 particles were bound to FX: where is this percentage shown? There is no equivalent to Figs 2b, 3a, 4a in Fig 1. It does not correlate with the particle numbers shown in Fig S2 or Table 1. Or do the authors mean Ad6 and are they referring to Fig 5d? Please clarify. Actually, a table summarizing these percentages for all specimens studied would be useful.

We thank the reviewer for the careful observation and for the helpful suggestions. We agree that the number of particles in the class averages were not clearly indicated. To improve clarity and consistency, we have added a new supplementary table (Supplementary Table 2) summarizing the percentages of factor-bound and factor-free particles for all datasets analyzed.

Lines 322–326: a sentence summarizing the relevant SPR results in reference [9] would be useful.

We have added a sentence summarizing the key SPR findings from reference [9] to provide quantitative context for coagulation factor binding to adenovirus hexons (lines 291-293).

Fig S1: The value corresponding to the scale bar seems to be missing.

We thank the reviewer for pointing this out. We agree that the original scale bar was difficult to see. We have now revised to improve its visibility by using a white scale bar (Supplementary Fig. 21).

Table 2: please clarify the origin of the data used for the unbound hexon structures. Do they correspond to the particle classes without clear density for the blood factors? Or were they imaged separately?

The unbound hexon structures were derived from particle subsets within the same datasets used for the hexon-factor complexes. Specifically, these correspond to particle classes identified during 3D classification that lacked clear density for the bound coagulation factors. These particles were assigned as factor-free and refined independently to obtain the unbound Ad5Hx and Ad6Hx reconstructions.

We have clarified this point in the Results section (lines 169-170, lines 187-189) and updated the figure legends of supplementary figures 8 and 13 to explicitly indicate the origin of the factor-free hexon datasets.

Fig 1b and 2c: when displaying AlphaFold predictions, a panel with the model colored by pLDDT should be included.

We thank the reviewer for this helpful suggestion. We have added panels showing the AlphaFold-predicted models colored by pLDDT confidence scores. pLDDT values for the AlphaFold models of FX and FII are 80.7 and 85.9 respectively (Supplementary Fig. 21).

Data availability (lines 446–449): if unbound hexon structures are also deposited (as indicated in Table 2), this should also be stated here.

We have revised the Data Availability statement to include the unbound hexon structures, ensuring consistency with Table 2 and the datasets described in the manuscript.
