# Supplementary material for: Structural requirements of blood factors binding to soluble hexon trimers with implications for adenovirus cell targeting and immune evasion
Source: PLoS Pathog. 2026 Jul 13;22(7):e1014389. doi: 10.1371/journal.ppat.1014389 (PMC13379079; doi:10.1371/journal.ppat.1014389)
Supplement: Peer review history

# Peer review: Decision Letter 0

**DOI:** 10.1371/journal.ppat.1014389.r001

**Authors:** Blossom Damania, Kinjal Majumder

31 Mar 2026

-->PPATHOGENS-D-26-00456

Structural requirements of blood factors binding to soluble hexon trimers with implications for adenovirus cell targeting and immune evasion.

PLOS Pathogens

Dear Dr. Reddy,

Thank you for submitting your manuscript to PLOS Pathogens. After careful consideration, we feel that it has merit but does not fully meet PLOS Pathogens's publication criteria as it currently stands. Therefore, we invite you to submit a revised version of the manuscript that addresses the points raised during the review process. In addition to addressing the individual points raised by all the reviewers, please focus on addressing the critiques raised by reviewers 1 and 3 about highlighting the novel aspects of your findings, both experimental and scientific, throughout the manuscript.

Please submit your revised manuscript by May 30 2026 11:59PM. If you will need more time than this to complete your revisions, please reply to this message or contact the journal office at plospathogens@plos.org. When you're ready to submit your revision, log on to https://www.editorialmanager.com/ppathogens/ and select the 'Submissions Needing Revision' folder to locate your manuscript file.

Please include the following items when submitting your revised manuscript:

* A letter that responds to each point raised by the editor and reviewer(s). You should upload this letter as a separate file labeled 'Response to Reviewers'. This file does not need to include responses to any formatting updates and technical items listed in the 'Journal Requirements' section below.

* A marked-up copy of your manuscript that highlights changes made to the original version. You should upload this as a separate file labeled 'Revised Manuscript with Track Changes'.

* An unmarked version of your revised paper without tracked changes. You should upload this as a separate file labeled 'Manuscript'.

If you would like to make changes to your financial disclosure, competing interests statement, or data availability statement, please make these updates within the submission form at the time of resubmission. Guidelines for resubmitting your figure files are available below the reviewer comments at the end of this letter.

We look forward to receiving your revised manuscript.

Kind regards,

Kinjal Majumder, PhD

Guest Editor

PLOS Pathogens

Blossom Damania

Section Editor

PLOS Pathogens

Sumita Bhaduri-McIntosh

Editor-in-Chief

PLOS Pathogens

orcid.org/0000-0003-2946-9497

Michael Malim

Editor-in-Chief

PLOS Pathogens

orcid.org/0000-0002-7699-2064

**Additional Editor Comments :**

Please address the significant critiques raised by reviewers 1 and 3 about the novel aspects of this manuscript that build on pre-existing biophysical and mutational findings in the literature.

**Journal Requirements:**

1) Please ensure that the CRediT author contributions listed for every co-author are completed accurately and in full.

At this stage, the following Authors/Authors require contributions: Olivia X Ma, Shao-Chia Lu, Haley E Mudrick, Mary E. Barry, Jarrod B. French, Michael A Barry, and Vijay S Reddy. Please ensure that the full contributions of each author are acknowledged in the "Add/Edit/Remove Authors" section of our submission form.

The list of CRediT author contributions may be found here: https://journals.plos.org/plospathogens/s/authorship#loc-author-contributions

2) We ask that a manuscript source file is provided at Revision. Please upload your manuscript file as a .doc, .docx, .rtf or .tex. If you are providing a .tex file, please upload it under the item type u2018LaTeX Source Fileu2019 and leave your .pdf version as the item type u2018Manuscriptu2019.

3) Please provide an Author Summary. This should appear in your manuscript between the Abstract (if applicable) and the Introduction, and should be 150-200 words long. The aim should be to make your findings accessible to a wide audience that includes both scientists and non-scientists. Sample summaries can be found on our website under Submission Guidelines:

https://journals.plos.org/plospathogens/s/submission-guidelines#loc-parts-of-a-submission

4) Please upload all main figures as separate Figure files in .tif or .eps format. For more information about how to convert and format your figure files please see our guidelines:

https://journals.plos.org/plospathogens/s/figures

5) Tables should not be uploaded as individual files in the Figures file. Please remove these files and include the Tables in your manuscript file as editable, cell-based objects. For more information about how to format tables, see our guidelines:

https://journals.plos.org/plospathogens/s/tables

6) We have noticed that you have uploaded Supporting Information files, but you have not included a list of legends. Please add a full list of legends for your Supporting Information files after the references list.

7) Please note that your Data Availability Statement is currently missing the DOI/accession number of each dataset OR a direct link to access each dataset. If your manuscript is accepted for publication, you will be asked to provide these details on a very short timeline. We therefore suggest that you provide this information now, though we will not hold up the peer review process if you are unable.

8) Please amend your detailed Financial Disclosure statement. This is published with the article. It must therefore be completed in full sentences and contain the exact wording you wish to be published.

1) State what role the funders took in the study. If the funders had no role in your study, please state: "The funders had no role in study design, data collection and analysis, decision to publish, or preparation of the manuscript."

9) Please ensure that the funders and grant numbers match between the Financial Disclosure field and the Funding Information tab in your submission form. Note that the funders must be provided in the same order in both places as well. Currently, "Hormel Foundation" is missing from the Financial Disclosure field.

Note: If the reviewer comments include a recommendation to cite specific previously published works, please review and evaluate these publications to determine whether they are relevant and should be cited. There is no requirement to cite these works unless the editor has indicated otherwise.

**Reviewers' Comments:**

Reviewer's Responses to Questions

**Part I - Summary**

Please use this section to discuss strengths/weaknesses of study, novelty/significance, general execution and scholarship.

Reviewer #1: In this manuscript, Ma and colleagues describe the structures of complexes of purified hexon protein trimers from HAdV-C5 and -C6 with two blood factors, X and II, which were previously known to bind to these proteins. Prior studies have also shown that HAdV-D26 does not bind to either factor. The authors then use alignment of the hexon from HAdV-D26 with the HAdV-5C/FX structure to propose a model in which failure to bind is due to the conformation of a hexon hypervariable loop in D26 that would sterically interfere with blood factor binding. The data and figures are clear and support the authors’ claims; however, there is very little novelty. The current structures are in almost complete agreement with prior structures of hexon/blood factor binding (RMSD ~0.51 angstroms for HAdV-C5/FX and 0.78 angstroms for HAdV-C5/FII) using whole virus rather than purified hexon. The authors also point out the structural and sequence similarities between the interacting (Gla) domains of FII and FX, which are consistent with the highly similar modes of binding reported previously and here. Finally, the results are consistent with previously reported biophysical and mutational studies. In short, the study overall is very limited in scope and presents essentially no new information.

Reviewer #2: The paper by Ma and coworkers reports on the cryoEM structure determination of three different adenovirus hexon types (2,5, 26) in complex with two different clotting factors (prothrombin and FX). Binding of clotting factors to adenovirus vectors is a potential complication in gene therapy and vaccine development and thus these studies are of special interest to this field.

Previous mutagenesis studies suggested that the clotting factors noted above, bind to the central cavity of the hexon trimer. However, the binding contribution by multiple amino acid hexon residues in the hypervariable regions 5 and 7 were not fully investigated. The current cryoEM studies at 3.2Å, aided by AI-based modeling (AlphaFold2), add important new details including the molecular basis for binding, pointing to a rationale for altering unwanted associations with clotting factors in vivo. The findings reported herein have the potential to make a significant impact on adenovirus based gene and vaccine development.

Reviewer #3: In this manuscript the authors solve structures of isolated hexons from adenovirus Ad5 and Ad6 in complex with blood factors X or II. They form the complexes by either incubating hexons separately with each blood factor, or with both factors together, to mimic the physiological situation where they are mixed in the blood. These authors have recently published a related report where they study the structure of complete Ad5 and Ad6 virions, also in complex with FX or FII (Mudrick et al Nat Com 2024). Here they attempt to obtain improved structural detail by studying binding to isolated hexon proteins, thereby removing the blurring effect of the massive icosahedral capsid on the non-icosahedrally bound factors. However, the structures largely confirm the previous results and do not seem to provide much new structural information. An interesting detail is the slight rearrangement of HVR5 in Ad6, seemingly to allow binding of FX/FII in a slightly narrower cavity than that in Ad5 hexon. Finally, a comparison is made with the Ad26 hexon structure in an attempt to find out why this adenovirus fails to bind (or binds more weekly) the same blood factors.

The study addresses a relevant question, fundamental to understand differences in biology between different adenovirus types and their repercussions when using the viruses as therapeutic vectors.

**********

**Part II – Major Issues: Key Experiments Required for Acceptance**

Please use this section to detail the key new experiments or modifications of existing experiments that should be absolutely required to validate study conclusions.

Generally, there should be no more than 3 such required experiments or major modifications for a "Major Revision" recommendation. If more than 3 experiments are necessary to validate the study conclusions, then you are encouraged to recommend "Reject".

Reviewer #1: (No Response)

Reviewer #2: none

Reviewer #3: 1. Given the similarity with the study by Mudrick et al 2024, I would suggest that the authors emphasize not only the differences in experimental strategy (as they do in lines 95-103), but also the new information provided by the present work.

2. The most novel aspect is the study of co-incubation with both blood factors. However, it is difficult to evaluate the preference for one or the other with the information provided, in particular in case of Ad5: the fit to the density shown in Fig 5c is not so clear. The authors might want to quantify the fit of both factors, for example using the Q-score metric, or feeding the density to an algorithm such as ModelAngelo.

3. It is also not clear if the authors maintained the blood factor:hexon ratio from the other experiments for either one of the factors in the co-incubation assays. That is: did they use 2 FX and 20 FII molecules per hexon trimer?.

4. In the same line, I am not sure if the authors can conclude that Ad6 binds FII preferentially (line 219), since in this experiment FII was present in a concentration 10-fold higher than FX. And this does not correlate with the amount of factor-bound hexons in the single incubation experiments, where there seem to be more Ad6 hexons with FX density (54%, fig 3a) than with FII (29%, Fig 4a). Finally, in line 283 it is stated that the conformational change of HVR5 “enables Ad6 hexon to accommodate both factors efficiently”, which seems a contradiction with line 219.

5. The authors should provide EMDB and PDB IDs and validation reports before final acceptance.

**********

**Part III – Minor Issues: Editorial and Data Presentation Modifications**

Please use this section for editorial suggestions as well as relatively minor modifications of existing data that would enhance clarity.

Reviewer #1: There are some minor concerns that would improve readability:

1. Refer to the viruses as HAdV-C5, HAdV-C6, and HAdV-D26 throughout.

2. Define “Gla domain” in the abstract.

3. There are more recent references than 4-6 to support the prominence of HAdV-based vectors in gene therapy and as vaccine platforms.

4. Line 67 – references here could be more inclusive of other studies cited elsewhere in the MS.

5. Line 140 – the residue numbers mentioned here don’t appear to match the numbers in Fig 1.

6. Fig 1ci – it would be helpful to distinguish the monomers, perhaps by different colors.

7. It’s a bit of an overstatement to call a single 1:10 mixture of FX and F11 in PBS “physiologic” in mimicking how these proteins might bind to HAdVs in serum, which is a much more complex mixture.

Reviewer #2: I only have minor suggestions to improve this very nice contribution as noted below.

1. Adding Figure numbers to the attached figures would be helpful.

2. Figure 2, difficult to visualize all 6 calcium ions bound to the hexon complexes containing each clotting factor. The authors should include a statement about this in the figure legend to clarify.

3. Figure 5. Does the preference of FX binding to Ad5 hexons and Fll binding to Ad6 hexons hold true for intact virions?

4. The authors should substantiate the preference for binding of clotting factors by performing BIAcore or EliSA assays.

5. Discussion (lines 253-254). This part deserves more description in terms of how clotting factors alter binding of viruses to host cells.

Reviewer #3: Line 52: a reference to the ICTV adenovirus taxonomy page would be appropriate.

Line 110 and 338: please indicate the composition of the DPBS buffer as well as the concentration of added Ca and Mg cations.

Line 149: Is ref. 20 correct here?

Line 269: “Ca ions are consistenly observed…” This sentence sounds a bit misleading. Do the authors mean that they observe density corresponding to these cations? This does not seem to be the case from the Results section. Please clarify.

Line 273: 80% of Ad5 particles were bound to FX: where is this percentage shown? There is no equivalent to Figs 2b, 3a, 4a in Fig 1. It does not correlate with the particle numbers shown in Fig S2 or Table 1. Or do the authors mean Ad6 and are they referring to Fig 5d? Please clarify. Actually, a table summarizing these percentages for all specimens studied would be useful.

Line 277: a sentence summarizing the relevant SPR results in reference [9] would be useful.

Fig S1: The value corresponding to the scale bar seems to be missing

Table 2: please clarify the origin of the data used for the unbound hexon structures. Do they correspond to the particle classes without clear density for the blood factors? Or were they imaged separately?

Fig 1b and 2c: when displaying AlphaFold predictions, a panel with the model colored by pLDDT should be included

Data availability (lines 390-392): if unbound hexon structures are also deposited (as indicated in Table 2), this should also be stated here.

**********

PLOS authors have the option to publish the peer review history of their article (what does this mean?). If published, this will include your full peer review and any attached files.

If you choose “no”, your identity will remain anonymous but your review may still be made public.

**Do you want your identity to be public for this peer review?**  For information about this choice, including consent withdrawal, please see our Privacy Policy.

Reviewer #1: No

Reviewer #2: No

Reviewer #3: No

[NOTE: If reviewer comments were submitted as an attachment file, they will be attached to this email and accessible via the submission site. Please log into your account, locate the manuscript record, and check for the action link "View Attachments". If this link does not appear, there are no attachment files.]

**Figure resubmission:**

-->While revising your submission, we strongly recommend that you use PLOS’s NAAS tool (https://ngplosjournals.pagemajik.ai/artanalysis) to test your figure files. NAAS can convert your figure files to the TIFF file type and meet basic requirements (such as print size, resolution), or provide you with a report on issues that do not meet our requirements and that NAAS cannot fix.

After uploading your figures to PLOS’s NAAS tool - https://ngplosjournals.pagemajik.ai/artanalysis, NAAS will process the files provided and display the results in the "Uploaded Files" section of the page as the processing is complete. If the uploaded figures meet our requirements (or NAAS is able to fix the files to meet our requirements), the figure will be marked as "fixed" above. If NAAS is unable to fix the files, a red "failed" label will appear above. When NAAS has confirmed that the figure files meet our requirements, please download the file via the download option, and include these NAAS processed figure files when submitting your revised manuscript.

**Reproducibility:**

To enhance the reproducibility of your results, we recommend that authors of applicable studies deposit laboratory protocols in protocols.io, where a protocol can be assigned its own identifier (DOI) such that it can be cited independently in the future. Additionally, PLOS ONE offers an option to publish peer-reviewed clinical study protocols. Read more information on sharing protocols at https://plos.org/protocols?utm_medium=editorial-email&utm_source=authorletters&utm_campaign=protocols

# Author comment: Author response to Decision Letter 1

**DOI:** 10.1371/journal.ppat.1014389.r002

13 May 2026

Attachment Submitted filename: PlosPathogena_Response-to-reviews_VR_edtd-5_Final.pdf

# Peer review: Decision Letter 1

**DOI:** 10.1371/journal.ppat.1014389.r003

**Authors:** Blossom Damania, Kinjal Majumder

19 Jun 2026

Dear Associate Professor Reddy,

We are pleased to inform you that your manuscript 'Structural requirements of blood factors binding to soluble hexon trimers with implications for adenovirus cell targeting and immune evasion.' has been provisionally accepted for publication in PLOS Pathogens.

Before your manuscript can be formally accepted you will need to complete some formatting changes, which you will receive in a follow up email. A member of our team will be in touch with a set of requests.

Please note that your manuscript will not be scheduled for publication until you have made the required changes, so a swift response is appreciated.

IMPORTANT: The editorial review process is now complete. PLOS will only permit corrections to spelling, formatting or significant scientific errors from this point onwards. Requests for major changes, or any which affect the scientific understanding of your work, will cause delays to the publication date of your manuscript.

Should you, your institution's press office or the journal office choose to press release your paper, you will automatically be opted out of early publication. We ask that you notify us now if you or your institution is planning to press release the article. All press must be co-ordinated with PLOS.

Thank you again for supporting Open Access publishing; we are looking forward to publishing your work in PLOS Pathogens.

Best regards,

Kinjal Majumder, PhD

Guest Editor

PLOS Pathogens

Blossom Damania

Section Editor

PLOS Pathogens

Sumita Bhaduri-McIntosh

Editor-in-Chief

PLOS Pathogens

orcid.org/0000-0003-2946-9497

Michael Malim

Editor-in-Chief

PLOS Pathogens

orcid.org/0000-0002-7699-2064

***********************************************************

Reviewer Comments (if any, and for reference):

Reviewer's Responses to Questions

**Part I - Summary**

Please use this section to discuss strengths/weaknesses of study, novelty/significance, general execution and scholarship.

Reviewer #1: In this revised manuscript from Ma et al., the authors have not adequately addressed the major concern that the study overall is very limited in scope and presents only incremental new information. Had the authors previously published work on interactions of purified hexon with blood factors and were now presenting whole virus structures, then perhaps there would be sufficient novelty. However, the opposite approach has merely provided incremental refinement to previously published structural data. In additional, all the findings using mixed blood factors are consistent with ample, previously published biophysical data.

Reviewer #2: The revised manuscript has been significantly improved. The major concern I previously had was that there wasn't sufficient data to justify a statement about the absolute difference in clotting factor binding between the different AdV types. In response to this concern, the authors have toned down this conclusion in the relevant sections of the paper and made appropriate textual changes to emphasize the lack of information needed to make such a conclusion.

Reviewer #3: The authors have adequately addressed my comments to the first version of the manuscript.

Reviewer #4: This manuscript provides substantial new high-resolution structural information about the binding of coagulation factors FX and FII to Ad5 and Ad6 hexon, along with comparison to Ad26, which has no ability to bind coagulation factors. This work follows on from the 2024 Mudrick et al. study from the same authors. The current work represents a significant advance because of the higher resolution and detailed elucidation of the reasons for the differences in coagulation factor binding, including the size of the opening of the hexon cavity, the size of the hydrophobic patch at the bottom of the cavity, specific features of the FX and FII Gla domains, and the flexibility of the hexon HVR5 domain. Overall, this study provides substantial new information that will allow targeted engineering of adenovirus hexon to manipulate or prevent binding of coagulation factors, with clear implications for using adenovirus vectors for gene therapy.

**********

**Part II – Major Issues: Key Experiments Required for Acceptance**

Please use this section to detail the key new experiments or modifications of existing experiments that should be absolutely required to validate study conclusions.

Generally, there should be no more than 3 such required experiments or major modifications for a "Major Revision" recommendation. If more than 3 experiments are necessary to validate the study conclusions, then you are encouraged to recommend "Reject".

Reviewer #1: 1. The study overall is very limited in scope and presents only incremental new information. Substantial novel information should be provided. To be specific, there are three purported major findings, 1) the identification of conserved and factor-specific interactions with HVR5 and HVR7, 2) distinct binding preferences for HAdV-C5 with FX and C6 with FII, and 3) the structural basis for a lack of HAdV-D26 binding to blood factors.

Finding 1 regarding conserved interactions has already been reported. Here is the relevant text from ref 31 for HAdV-C5 with FX: “In addition to some of the previously identified residues, 420-426 in HVR7 of Ad5-hexon, we also observed that E271 in HVR5 of three hexon subunits interact with multiple residues in the Gla domain. Significantly, F4 of the Gla-domain sits on top of three F458 residues from the hexon subunits and is involved in stacking interactions (Fig. 1g). Remarkably, the corresponding phenylalanine (F) residues in the species-C AdV hexons are conserved as well as in the Gla domains of coagulation factors, II, VII and X with exception of Factor-IX. The “greasy” interaction involving the aromatic (Phe) residues may allow the free rotation of the Gla domain in the hexon cavity. Figure 1g shows the Ad5-hexon residues that interact with the Gla domain of FX. Moreover, a critical HVR7 residue of Ad5:hexon, Thr425, has been shown to be important for FX binding is involved in the Ca2+ mediated interactions with Gla residues (Supplementary Table 2). In addition to allowing FX to bind to Ad5-hexons in multiple (3) modes, having a wider entrance to the hexon cavity (Supplementary Fig. 14) may result in higher affinities of association between FX and Ad5 (hexon) compared to other species-C Ads.”

Compared to the text from this manuscript: “Analysis of the refined model revealed two distinct binding regions on the HAdV-C5 hexon: one at the bottom of the hexon cavity and another at its entrance (Fig. 1c). At the bottom of the hexon cavity, residues I421 and F458 in the HVR7 loops from all three monomers form a hydrophobic “greasy patch” that engages residues F4 and L5 of the FX Gla domain through π-stacking and hydrophobic interactions (Fig. 1c–d). At the cavity entrance, residues T270 and E271 in HVR5 loop of one of the hexon subunits form additional contacts that help stabilize the bound FX Gla domain (Fig. 1c-d). In addition, a calcium-mediated interaction involving hexon residue T425, together with a salt bridge between FX-Gla residue H12 and hexon residue E271, further strengthens the interface (Fig. 1d and Supplementary Table 1). Our results confirm that HVR5 and HVR7 constitute the major binding regions for FX on the HAdV-C5 hexon. These findings are consistent with previous mutagenesis studies showing that substituting HVR5 and HVR7 of HAdV-C5 with the corresponding regions from HAdV-D26, which does not bind FX, markedly reduces factor binding [29].”

Ref31 also describes the interaction of HAdV-C6 with FX at 4.3 angstroms (compared to 3.3 angstroms in the current manuscript), of HAdV-C5 with FII at 4.13 angstroms (compared to 3.2 angstroms in the current manuscript), and of HAdV-C6 with FII at 3.7 angstroms (compared to 3.2 angstroms in the current manuscript). The authors acknowledge that the current structures are in almost complete agreement with prior structures of hexon/blood factor binding (RMSD ~0.51 angstroms for HAdV-C5/FX and 0.78 angstroms for HAdV-C5/FII) using whole virus rather than purified hexon.

Finding 2 has also already been reported. In ref 31: “Among the structures of Ad-FII complexes, the Ad6-FII complex yielded the highest resolution structure at 3.7 Å, consistent with the highest affinity of interactions observed between Ad6 and FII. As in the Ad5-FII structure, there are clear densities seen for both the Gla and KR1 domains, while the remaining KR2 and SP domains are disordered (Fig. 4). Superposition of Ad5 (hexon)-FII structure on to Ad6 (hexon)-FII structure suggests that FII binds deeply in the Ad6 hexon cavity compared to Ad5 hexon (Supplementary Fig. 10). Furthermore, we observe that twice as many residues in the FII-Gla domain interact with Ad6 hexon compared to Ad5 hexon (Supplementary Table 3) which is consistent with the higher affinity interaction between Ad6 hexon and FII (KD = 2.7 nM) compared to the interaction between Ad5 hexon and FII (KD = 39 nM). Again, we observe the conserved “greasy” interaction between F4 of the Gla domain and F469 residues from the Ad6 hexon subunits that allows the free movement of the Gla domain in the hexon cavity.”

In the current manuscript: “Together, these results indicate that factor selectivity is governed by different determinants in HAdV-C5 and HAdV-C6 hexons.”

If anything, the prior manuscript provided much more detail.

Finding 3 is based on modeling that is predicated on prior biophysical studies in refs 29 and 31. To perform the modeling, the authors utilized a HAdV-D26 hexon structure (PDB:7TAU) published in 2022. Although the superimposition of this structure on the latest HAdC-C5/FX structure is “new”, this analysis is an incremental advance.

2. It is not clear that there are factor-specific findings in the current study. Given that “[t]he high degree of sequence and structural conservation between the Gla domains of FX and FII, together with the nearly identical sets of hexon-contacting residues, further supports a shared binding mechanism”, it is not clear from the current study what the structural basis for the differing affinities of C5 and C6 for FII and FX is. If anything, more information was provided in ref 31. In this regard, the statement in lines 240-242 where the authors postulate that affinity vs availability explains the results of the mixed ligand experiment is a bit confusing, since it would appear that all of the occupancy findings are consistent with the previously determined relative affinities of purified viruses for FII and FX.

Reviewer #2: N/A

Reviewer #3: (No Response)

Reviewer #4: none

**********

**Part III – Minor Issues: Editorial and Data Presentation Modifications**

Please use this section for editorial suggestions as well as relatively minor modifications of existing data that would enhance clarity.

Reviewer #1: 1. The adenovirus naming convention (e.g., HAdV-D26) was applied as requested except in the new author summary (line 68). Please fix.

2. Line 74. To be accurate, the ICTV only regulates viral nomenclature to the level of species, while genotype/serotype designations are handled by the Human Adenovirus Working Group (http://hadvwg.gmu.edu/).

3. Line 109-110: “in relative abundance of 1:10 molar ratio reflecting their relative physiological abundance” is awkward.

4. The use of a consistent color scheme makes sense, but monomers in individual figures (e.g., Fig 1 c) could be distinguished by different shades of the cardinal colors to improve clarity

Reviewer #2: I appreciated the authors revision that states that not all the calcium binding sites can be discerned. And they also improved the Discussion by providing a bit more background on the mode of adenovirus association with receptors.

Reviewer #3: A couple of sentences in the main text seem a bit inconsistent with the information in Table S2, which states that ALL hexons in the Ad5-FX sample corresponded to a single, factor-containing class (lines 157-158: "One of the resulting classes" and 199-200: "Similar to the Ad5Hx-FX dataset"; line numbers as in the version with tracked changes).

Reviewer #4: none

**********

PLOS authors have the option to publish the peer review history of their article (what does this mean?). If published, this will include your full peer review and any attached files.

If you choose “no”, your identity will remain anonymous but your review may still be made public.

**Do you want your identity to be public for this peer review?**  For information about this choice, including consent withdrawal, please see our Privacy Policy.

Reviewer #1: No

Reviewer #2: No

Reviewer #3: No

Reviewer #4: No

# Editor report: Acceptance letter

**DOI:** 10.1371/journal.ppat.1014389.r004

**Authors:** Blossom Damania, Kinjal Majumder

Dear Associate Professor Reddy,

We are delighted to inform you that your manuscript, "Structural requirements of blood factors binding to soluble hexon trimers with implications for adenovirus cell targeting and immune evasion.," has been formally accepted for publication in PLOS Pathogens.

We have now passed your article onto the PLOS Production Department who will complete the rest of the pre-publication process. All authors will receive a confirmation email upon publication.

The corresponding author will soon be receiving a typeset proof for review, to ensure errors have not been introduced during production. Please review the PDF proof of your manuscript carefully, as this is the last chance to correct any scientific or type-setting errors. Please note that major changes, or those which affect the scientific understanding of the work, will likely cause delays to the publication date of your manuscript. Note: Proofs for Front Matter articles (Pearls, Reviews, Opinions, etc...) are generated on a different schedule and may not be made available as quickly.

Soon after your final files are uploaded, the early version of your manuscript, if you opted to have an early version of your article, will be published online. The date of the early version will be your article's publication date. The final article will be published to the same URL, and all versions of the paper will be accessible to readers.

For Research Articles, you will receive an invoice from PLOS for your publication fee after your manuscript has reached the completed accept phase. If you receive an email requesting payment before acceptance or for any other service, this may be a phishing scheme. Learn how to identify phishing emails and protect your accounts at https://explore.plos.org/phishing.

Thank you again for supporting open-access publishing; we are looking forward to publishing your work in PLOS Pathogens.

Best regards,

Sumita Bhaduri-McIntosh

Editor-in-Chief

PLOS Pathogens

orcid.org/0000-0003-2946-9497

Michael Malim

Editor-in-Chief

PLOS Pathogens

orcid.org/0000-0002-7699-2064
